# On Data-Processing and Majorization Inequalities for *f*-Divergences with Applications

**DOI:** 10.3390/e21101022

**Published:** 2019-10-21

**Authors:** Igal Sason

**Affiliations:** Department of Electrical Engineering, Technion—Israel Institute of Technology, Haifa 3200003, Israel; sason@ee.technion.ac.il; Tel.: +972-4-8294699

**Keywords:** contraction coefficient, data-processing inequalities, *f*-divergences, hypothesis testing, list decoding, majorization theory, Rényi information measures, Tsallis entropy, Tunstall trees

## Abstract

This paper is focused on the derivation of data-processing and majorization inequalities for *f*-divergences, and their applications in information theory and statistics. For the accessibility of the material, the main results are first introduced without proofs, followed by exemplifications of the theorems with further related analytical results, interpretations, and information-theoretic applications. One application refers to the performance analysis of list decoding with either fixed or variable list sizes; some earlier bounds on the list decoding error probability are reproduced in a unified way, and new bounds are obtained and exemplified numerically. Another application is related to a study of the quality of approximating a probability mass function, induced by the leaves of a Tunstall tree, by an equiprobable distribution. The compression rates of finite-length Tunstall codes are further analyzed for asserting their closeness to the Shannon entropy of a memoryless and stationary discrete source. Almost all the analysis is relegated to the appendices, which form the major part of this manuscript.

## 1. Introduction

Divergences are non-negative measures of dissimilarity between pairs of probability measures which are defined on the same measurable space. They play a key role in the development of information theory, probability theory, statistics, learning, signal processing, and other related fields. One important class of divergence measures is defined by means of convex functions *f*, and it is called the class of *f*-divergences. It unifies fundamental and independently-introduced concepts in several branches of mathematics such as the chi-squared test for the goodness of fit in statistics, the total variation distance in functional analysis, the relative entropy in information theory and statistics, and it is closely related to the Rényi divergence which generalizes the relative entropy. The class of *f*-divergences was introduced in the sixties by Ali and Silvey [1], Csiszár [2,3,4,5,6], and Morimoto [7]. This class satisfies pleasing features such as the data-processing inequality, convexity, continuity and duality properties, finding interesting applications in information theory and statistics (see, e.g., [4,6,8,9,10,11,12,13,14,15]).

This manuscript is a research paper which is focused on the derivation of data-processing and majorization inequalities for *f*-divergences, and a study of some of their potential applications in information theory and statistics. Preliminaries are next provided.

### 1.1. Preliminaries and Related Works

We provide here definitions and known results from the literature which serve as a background to the presentation in this paper. We first provide a definition for the family of *f*-divergences.

**Definition** **1** ([16], p. 4398)**.** *Let P and Q be probability measures, let μ be a dominating measure of P and Q (i.e., P,Q≪μ), and let $p:=\frac{dP}{d\mu }$ and $q:=\frac{dQ}{d\mu }$. The f-divergence from P to Q is given, independently of μ, by*

(1) $$\begin{array}{c} D_{f}\left(P∥Q\right):=\int q\;f\left(\frac{p}{q}\right)\;d\mu , \end{array}$$

*where*

(2) $$f\left(0\right):=\lim_{t\to 0^{+}}\;f\left(t\right),$$

(3) $$0f\left(\frac{0}{0}\right):=0,$$

(4) $$0f\left(\frac{a}{0}\right):=\lim_{t\to 0^{+}}\;tf\left(\frac{a}{t}\right)=a\lim_{u\to \infty }\frac{f(u)}{u},\;a>0.$$

**Definition** **2.** *Let $Q_{X}$ be a probability distribution which is defined on a set X, and that is not a point mass, and let $W_{Y|X}:X\to Y$ be a stochastic transformation. The contraction coefficient for f-divergences is defined as*

(5) $$\begin{array}{c} \mu_{f}\left(Q_{X},W_{Y|X}\right):=\underset{P_{X}:\;D_{f}\left(P_{X}∥Q_{X}\right)\in \left(0,\infty \right)}{\sup}\;\frac{D_{f}\left(P_{Y}∥Q_{Y}\right)}{D_{f}\left(P_{X}∥Q_{X}\right)}, \end{array}$$

*where, for all y∈Y,*

(6) $$P_{Y}\left(y\right)=\left(P_{X}W_{Y|X}\right)\;\left(y\right):=\int_{X}dP_{X}\left(x\right)\;W_{Y|X}\left(y|x\right),$$

(7) $$Q_{Y}\left(y\right)=\left(Q_{X}W_{Y|X}\right)\;\left(y\right):=\int_{X}dQ_{X}\left(x\right)\;W_{Y|X}\left(y|x\right).$$

*The notation in* (6) *and* (7)*, and also in* (20)*,* (21)*,* (42)*,* (43)*,* (44) *in the continuation of this paper, is consistent with the standard notation used in information theory (see, e.g., the first displayed equation after (3.2) in [17]).*

Contraction coefficients for *f*-divergences play a key role in strong data-processing inequalities (see [18,19,20], ([21], Chapter II), [22,23,24,25,26]). The following are essential definitions and results which are related to maximal correlation and strong data-processing inequalities.

**Definition** **3.** *The maximal correlation between two random variables X and Y is defined as*

(8) $$\begin{array}{c} \rho_{m}\left(X;Y\right):=\underset{f,g}{\sup}\;E\left[f\left(X\right)g\left(Y\right)\right], \end{array}$$

*where the supremum is taken over all real-valued functions f and g such that*

(9) $$\begin{array}{c} E\left[f\left(X\right)\right]=E\left[g\left(Y\right)\right]=0,\;E\left[f^{2}\left(X\right)\right]\leq 1,\;E\left[g^{2}\left(Y\right)\right]\leq 1. \end{array}$$

**Definition** **4.** *Pearson’s $\chi^{2}$-divergence [27] from P to Q is defined to be the f-divergence from P to Q (see Definition 1) with $f\left(t\right)={\left(t-1\right)}^{2}$ or $f\left(t\right)=t^{2}-1$ for all t>0,*

(10) $$\begin{array}{cc} \chi^{2}\left(P∥Q\right) & :=D_{f}\left(P∥Q\right) \end{array}$$

(11) $$\begin{array}{cc} & =\int \frac{{\left(p-q\right)}^{2}}{q}\;d\mu \end{array}$$

(12) $$\begin{array}{cc} & =\int \frac{p^{2}}{q}\;d\mu -1 \end{array}$$

*independently of the dominating measure μ (i.e., P,Q≪μ, e.g., μ=P+Q).*

*Neyman’s $\chi^{2}$-divergence [28] from P to Q is the Pearson’s $\chi^{2}$-divergence from Q to P, i.e., it is equal to*

(13) $$\begin{array}{c} \chi^{2}\left(Q∥P\right)=D_{g}\left(P∥Q\right) \end{array}$$

*with $g\left(t\right)=\frac{{\left(t-1\right)}^{2}}{t}$ or $g\left(t\right)=\frac{1}{t}-t$ for all t>0.*

**Proposition** **1** (([24], Theorem 3.2), [29])**.** *The contraction coefficient for the $\chi^{2}$-divergence satisfies*

(14) $$\begin{array}{c} \mu_{\chi^{2}}\left(Q_{X},W_{Y|X}\right)=\rho_{m}^{2}\left(X;Y\right) \end{array}$$

*with $X∼Q_{X}$ and $Y∼Q_{Y}$ (see* (7)*).*

**Proposition** **2** ([25], Theorem 2)**.** *Let f:(0,∞)→R be convex and twice continuously differentiable with f(1)=0 and $f^{\prime \prime }\left(1\right)>0$. Then, for any $Q_{X}$ that is not a point mass,*

(15) $$\begin{array}{c} \mu_{\chi^{2}}\left(Q_{X},W_{Y|X}\right)\leq \mu_{f}\left(Q_{X},W_{Y|X}\right), \end{array}$$

*i.e., the contraction coefficient for the $\chi^{2}$-divergence is the minimal contraction coefficient among all f-divergences with f satisfying the above conditions.*

**Remark** **1.** *A weaker version of* (15) *was presented in ([21], Proposition II.6.15) in the general alphabet setting, and the result in* (15) *was obtained in ([24], Theorem 3.3) for finite alphabets.*

The following result provides an upper bound on the contraction coefficient for a subclass of *f*-divergences in the finite alphabet setting.

**Proposition** **3** ([26], Theorem 8)**.** *Let f:[0,∞)→R be a continuous convex function which is three times differentiable at unity with f(1)=0 and $f^{\prime \prime }\left(1\right)>0$, and let it further satisfy the following conditions:*

*(a)*  (16) $$\begin{array}{c} \left(f\left(t\right)-f^{\prime }\left(1\right)\;\left(t-1\right)\right)\left(1-\frac{f^{\left(3\right)}\left(1\right)\left(t-1\right)}{3f^{\prime \prime }\left(1\right)}\right)\geq \frac{1}{2}f^{\prime \prime }\left(1\right){\left(t-1\right)}^{2},\;\forall \;t>0. \end{array}$$
*(b)*  The function g:(0,∞)→R, given by $g\left(t\right):=\frac{f(t)-f(0)}{t}$ for all t>0, is concave.

*Then, for a probability mass function $Q_{X}$ supported over a finite set X,*

(17) $$\begin{array}{c} \mu_{f}\left(Q_{X},W_{Y|X}\right)\leq \left(\frac{f^{\prime }\left(1\right)+f\left(0\right)}{f^{\prime \prime }\left(1\right)\;\min_{x\in X}\;Q_{X}\left(x\right)}\right)\mu_{\chi^{2}}\left(Q_{X},W_{Y|X}\right). \end{array}$$

For the presentation of our majorization inequalities for *f*-divergences and related entropy bounds (see Section 2.3), essential definitions and basic results are next provided (see, e.g., [30], ([31], Chapter 13) and ([32], Chapter 2)). Let *P* be a probability mass function defined on a finite set X, let $p_{\max}$ be the maximal mass of *P*, and let $G_{P}\left(k\right)$ be the sum of the *k* largest masses of *P* for k∈{1,…,|X|} (hence, it follows that $G_{P}\left(1\right)=p_{\max}$ and $G_{P}\left(|X|\right)=1$).

**Definition** **5.** *Consider discrete probability mass functions P and Q defined on a finite set X. It is said that P is majorized by Q (or Q majorizes P), and it is denoted by P≺Q, if $G_{P}\left(k\right)\leq G_{Q}\left(k\right)$ for all k∈{1,…,|X|} (recall that $G_{P}\left(|X|\right)=G_{Q}\left(|X|\right)=1$).*

A unit mass majorizes any other distribution; on the other hand, the equiprobable distribution on a finite set is majorized by any other distribution defined on the same set.

**Definition** **6.** *Let $P_{n}$ denote the set of all the probability mass functions that are defined on $A_{n}:=\left\{1,\ldots ,n\right\}$. A function $f:P_{n}\to R$ is said to be Schur-convex if for every $P,Q\in P_{n}$ such that P≺Q, we have f(P)≤f(Q). Likewise, f is said to be* Schur-concave *if −f is Schur-convex, i.e., $P,Q\in P_{n}$ and P≺Q imply that f(P)≥f(Q).*

Characterization of Schur-convex functions is provided, e.g., in ([30], Chapter 3). For example, there exist some connections between convexity and Schur-convexity (see, e.g., ([30], Section 3.C) and ([32], Chapter 2.3)). However, a Schur-convex function is not necessarily convex ([32], Example 2.3.15).

Finally, what is the connection between data processing and majorization, and why these types of inequalities are both considered in the same manuscript? This connection is provided in the following fundamental well-known result (see, e.g., ([32], Theorem 2.1.10), ([30], Theorem B.2) and ([31], Chapter 13)):

**Proposition** **4.** *Let P and Q be probability mass functions defined on a finite set A. Then, P≺Q if and only if there exists a doubly-stochastic transformation $W_{Y|X}:A\to A$ (i.e., $\sum_{x\in A}W_{Y|X}\left(y|x\right)=1$ for all y∈A, and $\sum_{y\in A}W_{Y|X}\left(y|x\right)=1$ for all x∈A with $W_{Y|X}\left(\cdot |\cdot \right)\geq 0$) such that $Q\to W_{Y|X}\to P$. In other words, P≺Q if and only if in their representation as column vectors, there exists a doubly-stochastic matrix W (i.e., a square matrix with non-negative entries such that the sum of each column or each row in W is equal to 1) such that P=WQ.*

### 1.2. Contributions

This paper is focused on the derivation of data-processing and majorization inequalities for *f*-divergences, and it applies these inequalities to information theory and statistics.

The starting point for obtaining strong data-processing inequalities in this paper relies on the derivation of lower and upper bounds on the difference $D_{f}\left(P_{X}∥Q_{X}\right)-D_{f}\left(P_{Y}∥Q_{Y}\right)$ where $\left(P_{X},Q_{X}\right)$ and $\left(P_{Y},Q_{Y}\right)$ denote, respectively, pairs of input and output probability distributions with a given stochastic transformation $W_{Y|X}$ (i.e., where $P_{X}\to W_{Y|X}\to P_{Y}$ and $Q_{X}\to W_{Y|X}\to Q_{Y}$). These bounds are expressed in terms of the respective difference in the Pearson’s or Neyman’s $\chi^{2}$-divergence, and they hold for all *f*-divergences (see Theorems 1 and 2). By a different approach, we derive an upper bound on the contraction coefficient for *f*-divergences of a certain type, which gives an alternative strong data-processing inequality for the considered type of *f*-divergences (see Theorems 3 and 4). In this framework, a parametric subclass of *f*-divergences is introduced, its interesting properties are studied (see Theorem 5), all the data-processing inequalities which are derived in this paper are applied to this subclass, and these inequalities are exemplified numerically to examine their tightness (see Section 3.1).

This paper also derives majorization inequalities for *f*-divergences where part of these inequalities rely on the earlier data-processing inequalities (see Theorem 6). A different approach, which relies on the concept of majorization, serves to derive tight bounds on the maximal value of an *f*-divergence from a probability mass function *P* to an equiprobable distribution; the maximization is carried over all *P* with a fixed finite support where the ratio of their maximal to minimal probability masses does not exceed a given value (see Theorem 7). These bounds lead to accurate asymptotic results which apply to general *f*-divergences, and they strengthen and generalize recent results of this type with respect to the relative entropy [33], and the Rényi divergence [34]. Furthermore, we explore in Theorem 7 the convergence rates to the asymptotic results. Data-processing and majorization inequalities also serve to strengthen the Schur-concavity property of the Tsallis entropy (see Theorem 8), showing by a comparison to earlier bounds in [35,36] that none of these bounds is superseded by the other. Further analytical results which are related to the specialization of our central result on majorization inequalities in Theorem 7, applied to several important sub-classes of *f*-divergences, are provided in Section 3.2 (including Theorem 9). A quantity which is involved in our majorization inequalities in Theorem 7 is interpreted by relying on a variational representation of *f*-divergences (see Theorem 10).

As an application of the data-processing inequalities for *f*-divergences, the setup of list decoding is further studied, reproducing in a unified way some known bounds on the list decoding error probability, and deriving new bounds for fixed and variable list sizes (see Theorems 11–13).

As an application of the majorization inequalities in this paper, we study properties of a measure which is used to quantify the quality of approximating probability mass functions, induced by the leaves of a Tunstall tree, by an equiprobable distribution (see Theorem 14). An application of majorization inequalities for the relative entropy is used to derive a sufficient condition, expressed in terms of the principal and secondary real branches of the Lambert *W* function [37], for asserting the proximity of compression rates of finite-length (lossless and variable-to-fixed) Tunstall codes to the Shannon entropy of a memoryless and stationary discrete source (see Theorem 15).

### 1.3. Paper Organization

The paper is structured as follows: Section 2 provides our main new results on data-processing and majorization inequalities for *f*-divergences and related entropy measures. Illustration of the theorems in Section 2, and further mathematical results which follow from these theorems are introduced in Section 3. Applications in information theory and statistics are considered in Section 4. Proofs of all theorems are relegated to the appendices, which form a major part of this paper.

## 2. Main Results on *f*-Divergences

This section provides strong data-processing inequalities for *f*-divergences (see Section 2.1), followed by a study of a new subclass of *f*-divergences (see Section 2.2) which later serves to exemplify our data-processing inequalities. The third part of this section (see Section 2.3) provides majorization inequalities for *f*-divergences, and for the Tsallis entropy, whose derivation relies in part on the new data-processing inequalities.

### 2.1. Data-Processing Inequalities for f-Divergences

Strong data-processing inequalities are provided in the following, bounding the difference $D_{f}\left(P_{X}∥Q_{X}\right)-D_{f}\left(P_{Y}∥Q_{Y}\right)$ and ratio $\frac{D_{f}\left(P_{Y}∥Q_{Y}\right)}{D_{f}\left(P_{X}∥Q_{X}\right)}$ where $\left(P_{X},Q_{X}\right)$ and $\left(P_{Y},Q_{Y}\right)$ denote, respectively, pairs of input and output probability distributions with a given stochastic transformation.

**Theorem** **1.** *Let X and Y be finite or countably infinite sets, let $P_{X}$ and $Q_{X}$ be probability mass functions that are supported on X, and let*

(18) $$\begin{array}{c} \xi_{1}:=\underset{x\in X}{\inf}\frac{P_{X}\left(x\right)}{Q_{X}\left(x\right)}\in \left[0,1\right], \end{array}$$

(19) $$\begin{array}{c} \xi_{2}:=\underset{x\in X}{\sup}\frac{P_{X}\left(x\right)}{Q_{X}\left(x\right)}\in \left[1,\infty \right]. \end{array}$$

*Let $W_{Y|X}:X\to Y$ be a stochastic transformation such that for every y∈Y, there exists x∈X with $W_{Y|X}\left(y|x\right)>0$, and let (see* (6) *and* (7)*)*

(20) $$\begin{array}{c} P_{Y}:=P_{X}W_{Y|X}, \end{array}$$

(21) $$\begin{array}{c} Q_{Y}:=Q_{X}W_{Y|X}. \end{array}$$

*Furthermore, let f:(0,∞)→R be a convex function with f(1)=0, and let the non-negative constant $c_{f}:=c_{f}\left(\xi_{1},\xi_{2}\right)$ satisfy*

(22) $$\begin{array}{c} f_{+}^{\prime }\left(v\right)-f_{+}^{\prime }\left(u\right)\geq 2c_{f}\;\left(v-u\right),\;\forall \;u,v\in I,\;u<v \end{array}$$

*where $f_{+}^{\prime }$ denotes the right-side derivative of f, and*

(23) $$\begin{array}{c} I:=I\left(\xi_{1},\xi_{2}\right)=\left[\xi_{1},\xi_{2}\right]\cap \left(0,\infty \right). \end{array}$$

*Then,*

*(a)*  (24) $$\begin{array}{cc} D_{f}\left(P_{X}∥Q_{X}\right)-D_{f}\left(P_{Y}∥Q_{Y}\right) & \geq c_{f}\left(\xi_{1},\xi_{2}\right)\left[\chi^{2}\left(P_{X}∥Q_{X}\right)-\chi^{2}\left(P_{Y}∥Q_{Y}\right)\right] \end{array}$$
  (25) $$\begin{array}{cc} & \geq 0, \end{array}$$
  *where equality holds in* (24) *if $D_{f}\left(\cdot ∥\cdot \right)$ is Pearson’s $\chi^{2}$-divergence with $c_{f}\equiv 1$.*
*(b)*  *If f is twice differentiable on I, then the largest possible coefficient in the right side of* (22) *is given by*
  (26) $$\begin{array}{c} c_{f}\left(\xi_{1},\xi_{2}\right)=\frac{1}{2}\;\underset{t\in I(\xi_{1},\xi_{2})}{\inf}f^{\prime \prime }\left(t\right). \end{array}$$
*(c)*  *Under the assumption in Item (b), the following dual inequality also holds:*
  (27) $$\begin{array}{cc} D_{f}\left(P_{X}∥Q_{X}\right)-D_{f}\left(P_{Y}∥Q_{Y}\right) & \geq c_{f^{*}}\;\left(\frac{1}{\xi_{2}},\frac{1}{\xi_{1}}\right)\;\left[\chi^{2}\left(Q_{X}∥P_{X}\right)-\chi^{2}\left(Q_{Y}∥P_{Y}\right)\right] \end{array}$$
  (28) $$\begin{array}{cc} & \geq 0, \end{array}$$
  *where $f^{*}:\left(0,\infty \right)\to R$ is the dual convex function which is given by*
  (29) $$\begin{array}{c} f^{*}\left(t\right):=t\;f\left(\frac{1}{t}\right),\;\forall \;t>0, \end{array}$$
  *and the coefficient in the right side of* (27) *satisfies*
  (30) $$\begin{array}{c} c_{f^{*}}\;\left(\frac{1}{\xi_{2}},\frac{1}{\xi_{1}}\right)=\frac{1}{2}\;\underset{t\in I(\xi_{1},\xi_{2})}{\inf}\left\{t^{3}\;f^{\prime \prime }\left(t\right)\right\} \end{array}$$
  *with the convention that $\frac{1}{\xi_{1}}=\infty$ if $\xi_{1}=0$. Equality holds in* (27) *if $D_{f}\left(\cdot ∥\cdot \right)$ is Neyman’s $\chi^{2}$-divergence (i.e., $D_{f}\left(P∥Q\right):=\chi^{2}\left(Q∥P\right)$ for all P and Q) with $c_{f^{*}}\equiv 1$.*
*(d)*  *Under the assumption in Item (b), if*
  (31) $$\begin{array}{c} e_{f}\left(\xi_{1},\xi_{2}\right):=\frac{1}{2}\;\underset{t\in I(\xi_{1},\xi_{2})}{\sup}f^{\prime \prime }\left(t\right)<\infty , \end{array}$$
  *then,*
  (32) $$\begin{array}{cc} D_{f}\left(P_{X}∥Q_{X}\right)-D_{f}\left(P_{Y}∥Q_{Y}\right)\; & \leq e_{f}\left(\xi_{1},\xi_{2}\right)\left[\chi^{2}\left(P_{X}∥Q_{X}\right)-\chi^{2}\left(P_{Y}∥Q_{Y}\right)\right]. \end{array}$$
  *Furthermore,*
  (33) $$\begin{array}{cc} D_{f}\left(P_{X}∥Q_{X}\right)-D_{f}\left(P_{Y}∥Q_{Y}\right)\; & \leq e_{f^{*}}\;\left(\frac{1}{\xi_{2}},\frac{1}{\xi_{1}}\right)\left[\chi^{2}\left(Q_{X}∥P_{X}\right)-\chi^{2}\left(Q_{Y}∥P_{Y}\right)\right] \end{array}$$
  *where the coefficient in the right side of* (33) *satisfies*
  (34) $$\begin{array}{c} e_{f^{*}}\;\left(\frac{1}{\xi_{2}},\frac{1}{\xi_{1}}\right)=\frac{1}{2}\;\underset{t\in I(\xi_{1},\xi_{2})}{\sup}\left\{t^{3}\;f^{\prime \prime }\left(t\right)\right\}, \end{array}$$
  *which is assumed to be finite. Equalities hold in* (32) *and* (33) *if $D_{f}\left(\cdot ∥\cdot \right)$ is Pearson’s or Neyman’s $\chi^{2}$-divergence with $e_{f}\equiv 1$ or $e_{f^{*}}\equiv 1$, respectively.*
*(e)*  *The lower and upper bounds in* (24)*,* (27)*,* (32) *and* (33) *are locally tight. More precisely, let $\left\{P_{X}^{\left(n\right)}\right\}$ be a sequence of probability mass functions defined on X and pointwise converging to $Q_{X}$ which is supported on X, and let $P_{Y}^{\left(n\right)}$ and $Q_{Y}$ be the probability mass functions defined on Y via* (20) *and* (21) *with inputs $P_{X}^{\left(n\right)}$ and $Q_{X}$, respectively. Suppose that*
  (35) $$\begin{array}{c} \lim_{n\to \infty }\underset{x\in X}{\inf}\frac{P_{X}^{\left(n\right)}\left(x\right)}{Q_{X}\left(x\right)}=1, \end{array}$$
  (36) $$\begin{array}{c} \lim_{n\to \infty }\underset{x\in X}{\sup}\frac{P_{X}^{\left(n\right)}\left(x\right)}{Q_{X}\left(x\right)}=1. \end{array}$$
*If f has a continuous second derivative at unity, then*
  (37) $$\begin{array}{c} \lim_{n\to \infty }\frac{D_{f}\left(P_{X}^{\left(n\right)}∥Q_{X}\right)-D_{f}\left(P_{Y}^{\left(n\right)}∥Q_{Y}\right)}{\chi^{2}\left(P_{X}^{\left(n\right)}∥Q_{X}\right)-\chi^{2}\left(P_{Y}^{\left(n\right)}∥Q_{Y}\right)}=\frac{1}{2}f^{\prime \prime }\left(1\right), \end{array}$$
  (38) $$\begin{array}{c} \lim_{n\to \infty }\frac{D_{f}\left(P_{X}^{\left(n\right)}∥Q_{X}\right)-D_{f}\left(P_{Y}^{\left(n\right)}∥Q_{Y}\right)}{\chi^{2}\left(Q_{X}∥P_{X}^{\left(n\right)}\right)-\chi^{2}\left(Q_{Y}∥P_{Y}^{\left(n\right)}\right)}=\frac{1}{2}f^{\prime \prime }\left(1\right), \end{array}$$
  *and these limits indicate the local tightness of the lower and upper bounds in Items (a)–(d).*

**Proof.**  See Appendix A.  □

An application of Theorem 1 gives the following result.

**Theorem** **2.** *Let X and Y be finite or countably infinite sets, let n∈N, and let $X^{n}:=\left(X_{1},\ldots ,X_{n}\right)$ and $Y^{n}:=\left(Y_{1},\ldots ,Y_{n}\right)$ be random vectors taking values on $X^{n}$ and $Y^{n}$, respectively. Let $P_{X^{n}}$ and $Q_{X^{n}}$ be the probability mass functions of discrete memoryless sources where, for all $\underline{x}\in X^{n}$,*

(39) $$\begin{array}{c} P_{X^{n}}\left(\underline{x}\right)=\prod_{i=1}^{n}P_{X_{i}}\left(x_{i}\right),\;Q_{X^{n}}\left(\underline{x}\right)=\prod_{i=1}^{n}Q_{X_{i}}\left(x_{i}\right), \end{array}$$

*with $P_{X_{i}}$ and $Q_{X_{i}}$ supported on X for all i∈{1,…,n}. Let each symbol $X_{i}$ be independently selected from one of the source outputs at time instant i with probabilities λ and 1−λ, respectively, and let it be transmitted over a discrete memoryless channel with transition probabilities*

(40) $$\begin{array}{c} W_{Y^{n}|X^{n}}\left(\underline{y}\;|\;\underline{x}\right)=\prod_{i=1}^{n}W_{Y_{i}|X_{i}}\left(y_{i}|x_{i}\right),\;\forall \;\underline{x}\in X^{n},\;\underline{y}\in Y^{n}. \end{array}$$

*Let $R_{X^{n}}^{\left(\lambda \right)}$ be the probability mass function of the symbols at the channel input, i.e.,*

(41) $$\begin{array}{c} R_{X^{n}}^{\left(\lambda \right)}\left(\underline{x}\right)=\prod_{i=1}^{n}\left(\lambda P_{X_{i}}\left(x_{i}\right)+\left(1-\lambda \right)Q_{X_{i}}\left(x_{i}\right)\right),\;\forall \;\underline{x}\in X^{n},\;\lambda \in \left[0,1\right], \end{array}$$

*let*

(42) $$\begin{array}{c} R_{Y^{n}}^{\left(\lambda \right)}:=R_{X^{n}}^{\left(\lambda \right)}\;W_{Y^{n}|X^{n}}, \end{array}$$

(43) $$\begin{array}{c} P_{Y^{n}}:=P_{X^{n}}W_{Y^{n}|X^{n}}, \end{array}$$

(44) $$\begin{array}{c} Q_{Y^{n}}:=Q_{X^{n}}W_{Y^{n}|X^{n}}, \end{array}$$

*and let f:(0,∞)→R be a convex and twice differentiable function with f(1)=0. Then,*

*(a)*  *For all λ∈[0,1],*
  $$\begin{array}{c} D_{f}\left(R_{X^{n}}^{\left(\lambda \right)}\;∥\;Q_{X^{n}}\right)-D_{f}\left(R_{Y^{n}}^{\left(\lambda \right)}\;∥\;Q_{Y^{n}}\right) \end{array}$$
  (45) $$\begin{array}{c} \geq c_{f}\left(\xi_{1}\left(n,\lambda \right),\;\xi_{2}\left(n,\lambda \right)\right)\left[\;\prod_{i=1}^{n}\left(1+\lambda^{2}\;\chi^{2}\left(P_{X_{i}}∥Q_{X_{i}}\right)\right)-\prod_{i=1}^{n}\left(1+\lambda^{2}\;\chi^{2}\left(P_{Y_{i}}∥Q_{Y_{i}}\right)\right)\right] \end{array}$$
  (46) $$\begin{array}{c} \geq c_{f}\left(\xi_{1}\left(n,\lambda \right),\;\xi_{2}\left(n,\lambda \right)\right)\;\lambda^{2}\;\sum_{i=1}^{n}\left[\chi^{2}\left(P_{X_{i}}∥Q_{X_{i}}\right)-\chi^{2}\left(P_{Y_{i}}∥Q_{Y_{i}}\right)\right]\geq 0, \end{array}$$
  *where $c_{f}\left(\cdot ,\cdot \right)$ in the right sides of* (45) *and* (46) *is given in* (26)*, and*
  (47) $$\begin{array}{c} \xi_{1}\left(n,\lambda \right):=\prod_{i=1}^{n}\left(1-\lambda +\lambda \;\underset{x\in X}{\inf}\frac{P_{X_{i}}\left(x\right)}{Q_{X_{i}}\left(x\right)}\right)\in \left[0,1\right], \end{array}$$
  (48) $$\begin{array}{c} \xi_{2}\left(n,\lambda \right):=\prod_{i=1}^{n}\left(1-\lambda +\lambda \;\underset{x\in X}{\sup}\frac{P_{X_{i}}\left(x\right)}{Q_{X_{i}}\left(x\right)}\right)\in \left[1,\infty \right]. \end{array}$$
*(b)*  *For all λ∈[0,1],*
  (49) $$\begin{array}{c} D_{f}\left(R_{X^{n}}^{\left(\lambda \right)}\;∥\;Q_{X^{n}}\right)-D_{f}\left(R_{Y^{n}}^{\left(\lambda \right)}\;∥\;Q_{Y^{n}}\right)\\ \leq e_{f}\left(\xi_{1}\left(n,\lambda \right),\;\xi_{2}\left(n,\lambda \right)\right)\left[\;\prod_{i=1}^{n}\left(1+\lambda^{2}\;\chi^{2}\left(P_{X_{i}}∥Q_{X_{i}}\right)\right)-\prod_{i=1}^{n}\left(1+\lambda^{2}\;\chi^{2}\left(P_{Y_{i}}∥Q_{Y_{i}}\right)\right)\right] \end{array}$$
  *where $e_{f}\left(\cdot ,\cdot \right)$, $\xi_{1}\left(\cdot ,\cdot \right)$ and $\xi_{2}\left(\cdot ,\cdot \right)$ in the right side of* (49) *are given in* (31)*,* (47) *and* (48)*, respectively.*
*(c)*  *If f has a continuous second derivative at unity, and $\underset{x\in X}{\sup}\frac{P_{X_{i}}\left(x\right)}{Q_{X_{i}}\left(x\right)}<\infty$ for all i∈{1,…,n}, then*
  (50) $$\begin{array}{c} \lim_{\lambda \to 0^{+}}\frac{D_{f}\left(R_{X^{n}}^{\left(\lambda \right)}\;∥\;Q_{X^{n}}\right)-D_{f}\left(R_{Y^{n}}^{\left(\lambda \right)}\;∥\;Q_{Y^{n}}\right)}{\lambda^{2}}\\ =\frac{1}{2}\;f^{\prime \prime }\left(1\right)\;\sum_{i=1}^{n}\left[\chi^{2}\left(P_{X_{i}}∥Q_{X_{i}}\right)-\chi^{2}\left(P_{Y_{i}}∥Q_{Y_{i}}\right)\right]. \end{array}$$
  *The lower bounds in the right sides of* (45) *and* (46)*, and the upper bound in the right side of* (49) *are tight as we let $\lambda \to 0^{+}$, yielding the limit in the right side of* (50)*.*

**Proof.**  See Appendix B.  □

**Remark** **2.** *Similar upper and lower bounds on $D_{f}\left(P_{X^{n}}\;∥\;R_{X^{n}}^{\left(\lambda \right)}\right)-D_{f}\left(P_{Y^{n}}\;∥\;R_{Y^{n}}^{\left(\lambda \right)}\right)$ can be obtained for all λ∈[0,1]. To that end, in* (45)*–*(49)*, one needs to replace f with $f^{*}$, switch between $P_{X_{i}}$ and $Q_{X_{i}}$ for all i, and replace λ with 1−λ.*

In continuation to ([26], Theorem 8) (see Proposition 3 in Section 1.1), we next provide an upper bound on the contraction coefficient for a subclass of *f*-divergences (this subclass is different from the one which is addressed in ([26], Theorem 8)). Although the first part of the next result is stated for finite or countably infinite alphabets, it is clear from its proof that it also holds in the general alphabet setting. Connections to the literature are provided in Remarks A1–A3.

**Theorem** **3.** *Let f:(0,∞)→R be a function which satisfies the following conditions:*

- *f is convex, differentiable at 1, f(1)=0, and $f\left(0\right):=\lim_{t\to 0^{+}}f\left(t\right)<\infty$;*
- *The function g:(0,∞)→R, defined for all t>0 by $g\left(t\right):=\frac{f(t)-f(0)}{t}$, is convex.*

*Let $P_{X}$ and $Q_{X}$ be non-identical probability mass functions which are defined on a finite or a countably infinite set X, and let*

(51) $$\begin{array}{c} \kappa \left(\xi_{1},\xi_{2}\right):=\underset{t\in \left(\xi_{1},1\right)\cup \left(1,\xi_{2}\right)}{\sup}\frac{f\left(t\right)+f^{\prime }\left(1\right)\;\left(1-t\right)}{{\left(t-1\right)}^{2}} \end{array}$$

*where $\xi_{1}\in \left[0,1\right)$ and $\xi_{2}\in \left(1,\infty \right]$ are given in* (18) *and* (19)*. Then, in the setting of* (20) *and* (21)*,*

(52) $$\frac{D_{f}\left(P_{Y}∥Q_{Y}\right)}{D_{f}\left(P_{X}∥Q_{X}\right)}\leq \frac{\kappa (\xi_{1},\xi_{2})}{f\left(0\right)+f^{\prime }\left(1\right)}\cdot \frac{\chi^{2}\left(P_{Y}∥Q_{Y}\right)}{\chi^{2}\left(P_{X}∥Q_{X}\right)}.$$

*Consequently, if $Q_{X}$ is finitely supported on X,*

(53) $$\mu_{f}\left(Q_{X},W_{Y|X}\right)\leq \frac{1}{f\left(0\right)+f^{\prime }\left(1\right)}\cdot \kappa \left(0,\frac{1}{\min_{x\in X}\;Q_{X}\left(x\right)}\right)\cdot \mu_{\chi^{2}}\left(Q_{X},W_{Y|X}\right).$$

**Proof.**  See Appendix C.1.  □

Similarly to the extension of Theorem 1 to Theorem 2, a similar extension of Theorem 3 leads to the following result.

**Theorem** **4.** *In the setting of* (39)*–*(44) *in Theorem 2, and under the assumptions on f in Theorem 3, the following holds for all λ∈(0,1] and n∈N:*

(54) $$\begin{array}{c} \frac{D_{f}\left(R_{Y^{n}}^{\left(\lambda \right)}\;∥\;Q_{Y^{n}}\right)}{D_{f}\left(R_{X^{n}}^{\left(\lambda \right)}\;∥\;Q_{X^{n}}\right)}\leq \frac{\kappa \left(\xi_{1}\left(n,\lambda \right),\;\xi_{2}\left(n,\lambda \right)\right)}{f\left(0\right)+f^{\prime }\left(1\right)}\;\frac{\overset{n}{\prod_{i=1}}\left(1+\lambda^{2}\;\chi^{2}(P_{Y_{i}}\;∥\;Q_{Y_{i}}\right))-1}{\overset{n}{\prod_{i=1}}\left(1+\lambda^{2}\;\chi^{2}(P_{X_{i}}\;∥\;Q_{X_{i}}\right))-1}, \end{array}$$

*with $\xi_{1}\left(n,\lambda \right)$ and $\xi_{2}\left(n,\lambda \right)$ and κ(·,·) defined in* (47)*,* (48) *and* (51)*, respectively.*

**Proof.**  See Appendix C.2.  □

### 2.2. A Subclass of *f*-Divergences

A subclass of *f*-divergences with interesting properties is introduced in Theorem 5. The data-processing inequalities in Theorems 2 and 4 are applied to these *f*-divergences in Section 3.

**Theorem** **5.** *Let $f_{\alpha }:\left[0,\infty \right)\to R$ be given by*

(55) $$\begin{array}{c} f_{\alpha }\left(t\right):={\left(\alpha +t\right)}^{2}\log\left(\alpha +t\right)-{\left(\alpha +1\right)}^{2}\log\left(\alpha +1\right),\;t\geq 0 \end{array}$$

*for all $\alpha \geq e^{-\frac{3}{2}}$. Then,*

*(a)*  *$D_{f_{\alpha }}\left(\cdot ∥\cdot \right)$ is an f-divergence which is monotonically increasing and concave in α, and its first three derivatives are related to the relative entropy and $\chi^{2}$-divergence as follows:*
  (56) $$\begin{array}{c} \frac{\partial }{\partial \alpha }\left\{D_{f_{\alpha }}\left(P∥Q\right)\right\}=2\left(\alpha +1\right)\;D\left(\frac{\alpha Q+P}{\alpha +1}\;∥\;Q\right), \end{array}$$
  (57) $$\begin{array}{c} \frac{\partial^{2}}{\partial \alpha^{2}}\left\{D_{f_{\alpha }}\left(P∥Q\right)\right\}=-2\;D\left(Q\;∥\;\frac{\alpha Q+P}{\alpha +1}\right), \end{array}$$
  (58) $$\begin{array}{c} \frac{\partial^{3}}{\partial \alpha^{3}}\left\{D_{f_{\alpha }}\left(P∥Q\right)\right\}=\frac{2\log e}{\alpha +1}\cdot \chi^{2}\left(Q\;∥\;\frac{\alpha Q+P}{\alpha +1}\right). \end{array}$$
*(b)*  *For every n∈N,*
  (59) $$\begin{array}{c} {\left(-1\right)}^{n-1}\;\frac{\partial^{n}}{\partial \alpha^{n}}\left\{D_{f_{\alpha }}\left(P∥Q\right)\right\}\geq 0, \end{array}$$
  *and, in addition to (56)–(58), for all n>3*
  (60) $$\begin{array}{c} \frac{\partial^{n}}{\partial \alpha^{n}}\left\{D_{f_{\alpha }}\left(P∥Q\right)\right\}=\frac{2{\left(-1\right)}^{n-1}\left(n-3\right)!\;\log e}{{\left(\alpha +1\right)}^{n-2}}\left[\exp\left(\left(n-2\right)\;D_{n-1}\left(Q\;∥\;\frac{\alpha Q+P}{\alpha +1}\right)\right)-1\right], \end{array}$$
  *where $D_{n-1}\left(\cdot ∥\cdot \right)$ in the right side of (60) denotes the Rényi divergence of order n−1.*
*(c)*  (61) $$\begin{array}{cc} D_{f_{\alpha }}\left(P∥Q\right) & \geq k\left(\alpha \right)\;\chi^{2}\left(P∥Q\right) \end{array}$$
  (62) $$\begin{array}{cc} & \geq k\left(\alpha \right)\;\left[\exp\left(D(P∥Q)\right)-1\right] \end{array}$$
  *where the function $k:[e^{-\frac{3}{2}},\infty )\to R$ is defined as*
  (63) $$\begin{array}{c} k\left(\alpha \right):=\log\left(\alpha +1\right)+\frac{3}{2}\log e-\frac{\log e}{3\alpha }, \end{array}$$
  *which is monotonically increasing in α, satisfying k(α)≥0.2075loge for all $\alpha \geq e^{-\frac{3}{2}}$, and it tends to infinity as we let α→∞. Consequently, unless P≡Q,*
  (64) $$\begin{array}{c} \lim_{\alpha \to \infty }D_{f_{\alpha }}\left(P∥Q\right)=+\infty . \end{array}$$
*(d)*  (65) $$\begin{array}{cc} D_{f_{\alpha }}\left(P∥Q\right) & \leq \left[\log\left(\alpha +1\right)+\frac{3}{2}\log e-\frac{\log e}{\alpha +1}\right]\;\chi^{2}\left(P∥Q\right)+\frac{\log e}{3(\alpha +1)}\left[\exp\left(2D_{3}\left(P∥Q\right)\right)-1\right]. \end{array}$$
*(e)*  *For every ε>0 and a pair of probability mass functions (P,Q) where $D_{3}\left(P∥Q\right)<\infty$, there exists $\alpha^{*}:=\alpha \left(P,Q,\varepsilon \right)$ such that for all $\alpha >\alpha^{*}$*
  (66) $$\begin{array}{c} \left|D_{f_{\alpha }}\left(P∥Q\right)-\left[\log\left(\alpha +1\right)+\frac{3}{2}\log e\right]\;\chi^{2}\left(P∥Q\right)\right|<\varepsilon . \end{array}$$
*(f)*  *If a sequence of probability measures $\left\{P_{n}\right\}$ converges to a probability measure Q such that*
  (67) $$\begin{array}{c} \lim_{n\to \infty }\mathrm{ess}\;\sup\;\frac{dP_{n}}{dQ}\;\left(Y\right)=1,\;Y∼Q, \end{array}$$
  *where $P_{n}\ll Q$ for all sufficiently large n, then*
  (68) $$\begin{array}{c} \lim_{n\to \infty }\frac{D_{f_{\alpha }}\left(P_{n}∥Q\right)}{\chi^{2}\left(P_{n}∥Q\right)}=\log\left(\alpha +1\right)+\frac{3}{2}\log e. \end{array}$$
*(g)*  *If $\alpha >\beta \geq e^{-\frac{3}{2}}$, then*
  (69) $$\begin{array}{cc} 0 & \leq \left(\alpha -\beta \right)\left(\alpha +\beta +2\right)\;D\left(\frac{\alpha Q+P}{\alpha +1}\;∥\;Q\right) \end{array}$$
  (70) $$\begin{array}{cc} & \leq D_{f_{\alpha }}\left(P∥Q\right)-D_{f_{\beta }}\left(P∥Q\right) \end{array}$$
  (71) $$\begin{array}{cc} & \leq \left(\alpha -\beta \right)\;\min\left\{\left(\alpha +\beta +2\right)\;D\left(\frac{\beta Q+P}{\beta +1}\;∥\;Q\right),\;2D\left(P∥Q\right)\right\}. \end{array}$$
*(h)*  *The function $f_{\alpha }:\left[0,\infty \right)\to R$, as given in* (55)*, satisfies the conditions in Theorems 3 and 4 for all $\alpha \geq e^{-\frac{3}{2}}$. Furthermore, the corresponding function in* (51) *is equal to*
  (72) $$\begin{array}{cc} \kappa_{\alpha }\left(\xi_{1},\xi_{2}\right) & :=\underset{t\in \left(\xi_{1},1\right)\cup \left(1,\xi_{2}\right)}{\sup}\frac{f_{\alpha }\left(t\right)+f_{\alpha }^{\prime }\left(1\right)\;\left(1-t\right)}{{\left(t-1\right)}^{2}} \end{array}$$
  (73) $$\begin{array}{cc} & =\frac{f_{\alpha }\left(\xi_{2}\right)+f_{\alpha }^{\prime }\left(1\right)\;\left(1-\xi_{2}\right)}{{\left(\xi_{2}-1\right)}^{2}} \end{array}$$
  *for all $\xi_{1}\in \left[0,1\right)$ and $\xi_{2}\in \left(1,\infty \right)$.*

**Proof.**  See Appendix D.  □

### 2.3. *f*-Divergence Inequalities via Majorization

Let $U_{n}$ denote an equiprobable probability mass function on {1,…,n} for an arbitrary n∈N, i.e., $U_{n}\left(i\right):=\frac{1}{n}$ for all i∈{1,…,n}. By majorization theory and Theorem 1, the next result strengthens the Schur-convexity property of the *f*-divergence $D_{f}\left(\cdot ∥U_{n}\right)$ (see ([38], Lemma 1)).

**Theorem** **6.** *Let P and Q be probability mass functions which are supported on {1,…,n}, and suppose that P≺Q. Let f:(0,∞)→R be twice differentiable and convex with f(1)=0, and let $q_{\max}$ and $q_{\min}$ be, respectively, the maximal and minimal positive masses of Q. Then,*

*(a)*  $$\begin{array}{cc} \; & ne_{f}\left(nq_{\min},nq_{\max}\right)\;\left({∥Q∥}_{2}^{2}-{∥P∥}_{2}^{2}\right) \end{array}$$
  (74) $$\begin{array}{cc} \; & \geq D_{f}\left(Q∥U_{n}\right)-D_{f}\left(P∥U_{n}\right) \end{array}$$
  (75) $$\begin{array}{cc} \; & \geq nc_{f}\left(nq_{\min},nq_{\max}\right)\;\left({∥Q∥}_{2}^{2}-{∥P∥}_{2}^{2}\right)\geq 0, \end{array}$$
  *where $c_{f}\left(\cdot ,\cdot \right)$ and $e_{f}\left(\cdot ,\cdot \right)$ are given in* (26) *and* (31)*, respectively, and ${∥\cdot ∥}_{2}$ denotes the Euclidean norm. Furthermore,* (74) *and* (75) *hold with equality if $D_{f}\left(\cdot ∥\cdot \right)=\chi^{2}\left(\cdot ∥\cdot \right)$.*
*(b)*  *If P≺Q and $\frac{q_{\max}}{q_{\min}}\leq \rho$ for an arbitrary ρ≥1, then*
  (76) $$\begin{array}{c} 0\leq {∥Q∥}_{2}^{2}-{∥P∥}_{2}^{2}\leq \frac{{\left(\rho -1\right)}^{2}}{4\rho n}. \end{array}$$

**Proof.**  See Appendix E.  □

**Remark** **3.** *If P is not supported on {1,…,n}, then* (74) *and* (75) *hold if f is also right continuous at zero.*

The next result provides upper and lower bounds on *f*-divergences from any probability mass function to an equiprobable distribution. It relies on majorization theory, and it follows in part from Theorem 6.

**Theorem** **7.** *Let $P_{n}$ denote the set of all the probability mass functions that are defined on $A_{n}:=\left\{1,\ldots ,n\right\}$. For ρ≥1, let $P_{n}\left(\rho \right)$ be the set of all $Q\in P_{n}$ which are supported on $A_{n}$ with $\frac{q_{\max}}{q_{\min}}\leq \rho$, and let f:(0,∞)→R be a convex function with f(1)=0. Then,*

*(a)*  *The set $P_{n}\left(\rho \right)$, for any ρ≥1, is a non-empty, convex and compact set.*
*(b)*  *For a given $Q\in P_{n}$, which is supported on $A_{n}$, the f-divergences $D_{f}\left(\cdot ∥Q\right)$ and $D_{f}\left(Q∥\cdot \right)$ attain their maximal values over the set $P_{n}\left(\rho \right)$.*
*(c)*  *For ρ≥1 and an integer n≥2, let*
  (77) $$\begin{array}{cc} \; & u_{f}\left(n,\rho \right):=\max_{Q\in P_{n}\left(\rho \right)}D_{f}\left(Q∥U_{n}\right), \end{array}$$
  (78) $$\begin{array}{cc} \; & v_{f}\left(n,\rho \right):=\max_{Q\in P_{n}\left(\rho \right)}D_{f}\left(U_{n}∥Q\right), \end{array}$$
  *let*
  (79) $$\begin{array}{c} \Gamma_{n}\left(\rho \right):=\left[\frac{1}{1+(n-1)\rho },\;\frac{1}{n}\right], \end{array}$$
  *and let the probability mass function $Q_{\beta }\in P_{n}\left(\rho \right)$ be defined on the set $A_{n}$ as follows:*
  (80) $$Q_{\beta }\left(j\right):=\{\begin{array}{cc} \rho \beta , & \;if\;j\in \{1,\ldots ,i_{\beta }\},\\ 1-\left(n+i_{\beta }\left(\rho -1\right)-1\right)\beta , & \;if\;j=i_{\beta }+1,\\ \beta , & \;if\;j\in \{i_{\beta }+2,\ldots ,n\} \end{array}$$
  *where*
  (81) $$\begin{array}{c} i_{\beta }:=\left\lfloor \frac{1-n\beta }{(\rho -1)\beta }\right\rfloor . \end{array}$$
*Then,*
  (82) $$\begin{array}{cc} \; & u_{f}\left(n,\rho \right)=\max_{\beta \in \Gamma_{n}\left(\rho \right)}D_{f}\left(Q_{\beta }∥U_{n}\right), \end{array}$$
  (83) $$\begin{array}{cc} \; & v_{f}\left(n,\rho \right)=\max_{\beta \in \Gamma_{n}\left(\rho \right)}D_{f}\left(U_{n}∥Q_{\beta }\right). \end{array}$$
*(d)*  *For ρ≥1 and an integer n≥2, let the non-negative function $g_{f}^{\left(\rho \right)}:\left[0,1\right]\to R_{+}$ be given by*
  (84) $$\begin{array}{c} g_{f}^{\left(\rho \right)}\left(x\right):=x\;f\left(\frac{\rho }{1+(\rho -1)x}\right)+\left(1-x\right)\;f\left(\frac{1}{1+(\rho -1)x}\right),\;x\in \left[0,1\right]. \end{array}$$
  *Then,*
  (85) $$\begin{array}{cc} \; & \max_{m\in \{0,\ldots ,n\}}\;g_{f}^{\left(\rho \right)}\left(\frac{m}{n}\right)\leq u_{f}\left(n,\rho \right)\leq \max_{x\in [0,1]}\;g_{f}^{\left(\rho \right)}\left(x\right), \end{array}$$
  (86) $$\begin{array}{cc} \; & \max_{m\in \{0,\ldots ,n\}}\;g_{f^{*}}^{\left(\rho \right)}\left(\frac{m}{n}\right)\leq v_{f}\left(n,\rho \right)\leq \max_{x\in [0,1]}\;g_{f^{*}}^{\left(\rho \right)}\left(x\right) \end{array}$$
  *with the convex function $f^{*}:\left(0,\infty \right)\to R$ in* (29)*.*
*(e)*  *The right-side inequalities in* (85) *and* (86) *are asymptotically tight (n→∞). More explicitly,*
  (87) $$\begin{array}{cc} \; & \lim_{n\to \infty }u_{f}\left(n,\rho \right)=\max_{x\in [0,1]}\left\{x\;f\left(\frac{\rho }{1+(\rho -1)x}\right)+\left(1-x\right)\;f\left(\frac{1}{1+(\rho -1)x}\right)\right\}, \end{array}$$
  (88) $$\begin{array}{cc} \; & \lim_{n\to \infty }v_{f}\left(n,\rho \right)=\max_{x\in [0,1]}\left\{\frac{\rho x}{1+(\rho -1)x}\;\;f\left(\frac{1+(\rho -1)x}{\rho }\right)+\frac{\left(1-x\right)\;f\left(1+(\rho -1)x\right)}{1+(\rho -1)x}\right\}. \end{array}$$
*(f)*  *If $g_{f}^{\left(\rho \right)}\left(\cdot \right)$ in* (84) *is differentiable on (0,1) and its derivative is upper bounded by $K_{f}\left(\rho \right)\geq 0$, then for every integer n≥2*
  (89) $$\begin{array}{c} 0\leq \lim_{n^{\prime }\to \infty }\left\{u_{f}\left(n^{\prime },\rho \right)\right\}-u_{f}\left(n,\rho \right)\leq \frac{K_{f}\left(\rho \right)}{n}. \end{array}$$
*(g)*  *Let $f\left(0\right):=\lim_{t\to 0}\;f\left(t\right)\in \left(-\infty ,+\infty \right]$, and let n≥2 be an integer. Then,*
  (90) $$\begin{array}{cc} \; & \lim_{\rho \to \infty }u_{f}\left(n,\rho \right)=\left(1-\frac{1}{n}\right)f\left(0\right)+\frac{f(n)}{n}. \end{array}$$
*Furthermore, if f(0)<∞, f is differentiable on (0,n), and $K_{n}:=\underset{t\in (0,n)}{\sup}\;\left|f^{\prime }\left(t\right)\right|<\infty$, then, for every ρ≥1,*
  (91) $$\begin{array}{c} 0\leq \lim_{\rho^{\prime }\to \infty }\left\{u_{f}\left(n,\rho^{\prime }\right)\right\}-u_{f}\left(n,\rho \right)\leq \frac{2K_{n}\;\left(n-1\right)}{n+\rho -1}. \end{array}$$
*(h)*  *For ρ≥1, let the function f be also twice differentiable, and let M and m be constants such that the following condition holds:*
  (92) $$\begin{array}{c} 0\leq m\leq f^{\prime \prime }\left(t\right)\leq M,\;\forall \;t\in \left[\frac{1}{\rho },\rho \right]. \end{array}$$
  *Then, for all $Q\in P_{n}\left(\rho \right)$,*
  (93) $$\begin{array}{cc} 0 & \leq \frac{1}{2}m\left({n∥Q∥}_{2}^{2}-1\right) \end{array}$$
  (94) $$\begin{array}{cc} \; & \leq D_{f}\left(Q∥U_{n}\right) \end{array}$$
  (95) $$\begin{array}{cc} \; & \leq \frac{1}{2}M\left({n∥Q∥}_{2}^{2}-1\right) \end{array}$$
  (96) $$\begin{array}{cc} \; & \leq \frac{M{\left(\rho -1\right)}^{2}}{8\rho } \end{array}$$
  *with equalities in* (94) *and* (95) *for the $\chi^{2}$ divergence (with M=m=2).*
*(i)*  *Let d>0. If $f^{\prime \prime }\left(t\right)\leq M_{f}\in \left(0,\infty \right)$ for all t>0, then $D_{f}\left(Q∥U_{n}\right)\leq d$ for all $Q\in P_{n}\left(\rho \right)$, if*
  (97) $$\begin{array}{c} \rho \leq 1+\frac{4d}{M_{f}}+\sqrt{\frac{8d}{M_{f}}+\frac{16d^{2}}{M_{f}^{2}}}. \end{array}$$

**Proof.**  See Appendix F.  □

Tsallis entropy was introduced in [39] as a generalization of the Shannon entropy (similarly to the Rényi entropy [40]), and it was applied to statistical physics in [39].

**Definition** **7** ([39])**.** *Let $P_{X}$ be a probability mass function defined on a discrete set X. The Tsallis entropy of order α∈(0,1)∪(1,∞) of X, denoted by $S_{\alpha }\left(X\right)$ or $S_{\alpha }\left(P_{X}\right)$, is defined as*

(98) $$\begin{array}{cc} S_{\alpha }\left(X\right) & =\frac{1}{1-\alpha }\left(\;\sum_{x\in X}P_{X}^{\alpha }\left(x\right)-1\right) \end{array}$$

(99) $$\begin{array}{cc} \; & =\frac{∥P_{X}{∥}_{\alpha }^{\alpha }-1}{1-\alpha }, \end{array}$$

*where ${∥P_{X}∥}_{\alpha }:={\left(\;\sum_{x\in X}P_{X}^{\alpha }\left(x\right)\right)}^{\frac{1}{\alpha }}$. The Tsallis entropy is continuously extended at orders* 0*,* 1*, and* ∞*; at order 1, it coincides with the Shannon entropy on base e (expressed in nats).*

Theorem 6 enables to strengthen the Schur-concavity property of the Tsallis entropy (see ([30], Theorem 13.F.3.a.)) as follows.

**Theorem** **8.** *Let P and Q be probability mass functions which are supported on a finite set, and let P≺Q. Then, for all α>0,*

*(a)*  (100) $$0\leq L\left(\alpha ,P,Q\right)\leq S_{\alpha }\left(P\right)-S_{\alpha }\left(Q\right)\leq U\left(\alpha ,P,Q\right),$$
  *where*
  (101) $$L\left(\alpha ,P,Q\right):=\{\begin{array}{cc} \frac{1}{2}\;\alpha q_{\max}^{\alpha -2}\;\left({∥Q∥}_{2}^{2}-{∥P∥}_{2}^{2}\right),\; & if\;\alpha \in (0,2],\\ \frac{1}{2}\;\alpha q_{\min}^{\alpha -2}\;\left({∥Q∥}_{2}^{2}-{∥P∥}_{2}^{2}\right),\; & if\;\alpha \in (2,\infty ), \end{array}$$
  (102) $$U\left(\alpha ,P,Q\right):=\{\begin{array}{cc} \frac{1}{2}\;\alpha q_{\min}^{\alpha -2}\;\left({∥Q∥}_{2}^{2}-{∥P∥}_{2}^{2}\right),\; & if\;\alpha \in (0,2],\\ \frac{1}{2}\;\alpha q_{\max}^{\alpha -2}\;\left({∥Q∥}_{2}^{2}-{∥P∥}_{2}^{2}\right),\; & if\;\alpha \in (2,\infty ), \end{array}$$
  *and the bounds in* (101) *and* (102) *are attained at α=2.*
*(b)*  (103) $$\underset{P≺Q,\;P\neq Q}{\inf}\frac{S_{\alpha }\left(P\right)-S_{\alpha }\left(Q\right)}{L(\alpha ,P,Q)}=\underset{P≺Q,\;P\neq Q}{\sup}\frac{S_{\alpha }\left(P\right)-S_{\alpha }\left(Q\right)}{U(\alpha ,P,Q)}=1,$$
  *where the infimum and supremum in* (103) *can be restricted to probability mass functions P and Q which are supported on a binary alphabet.*

**Proof.**  See Appendix G.  □

**Remark** **4.** *The lower bound in ([36], Theorem 1) also strengthens the Schur-concavity property of the Tsallis entropy. It can be verified that none of the lower bounds in ([36], Theorem 1) and Theorem 8 supersedes the other. For example, let α>0, and let $P_{\varepsilon }$ and $Q_{\varepsilon }$ be probability mass functions supported on A:={0,1} with $P_{\varepsilon }\left(0\right)=\frac{1}{2}+\varepsilon$ and $Q_{\varepsilon }\left(0\right)=\frac{1}{2}+\beta \varepsilon$ where β>1 and $0<\varepsilon <\frac{1}{2\beta }$. This yields $P_{\varepsilon }≺Q_{\varepsilon }$. From* (A233) *(see Appendix G),*

(104) $$\begin{array}{cc} \; & \lim_{\varepsilon \to 0^{+}}\frac{S_{\alpha }\left(P_{\varepsilon }\right)-S_{\alpha }\left(Q_{\varepsilon }\right)}{L(\alpha ,P_{\varepsilon },Q_{\varepsilon })}=1. \end{array}$$

*If α=1, then $S_{1}\left(P_{\varepsilon }\right)-S_{1}\left(Q_{\varepsilon }\right)=\frac{1}{\log e}\left(H\left(P_{\varepsilon }\right)-H\left(Q_{\varepsilon }\right)\right)$, and the continuous extension of the lower bound in ([36], Theorem 1) at α=1 is specialized to the earlier result by the same authors in ([35], Theorem 3); it states that if P≺Q, then H(P)−H(Q)≥D(Q∥P). In contrast to* (104)*, it can be verified that*

(105) $$\begin{array}{cc} \; & \lim_{\varepsilon \to 0^{+}}\frac{S_{1}\left(P_{\varepsilon }\right)-S_{1}\left(Q_{\varepsilon }\right)}{\frac{1}{\log e}\;D\left(Q_{\varepsilon }∥P_{\varepsilon }\right)}=\frac{\beta +1}{\beta -1}>1,\;\forall \;\beta >1, \end{array}$$

*which can be made arbitrarily large by selecting β to be sufficiently close to 1 (from above). This provides a case where the lower bound in Theorem 8 outperforms the one in ([35], Theorem 3).*

**Remark** **5.** *Due to the one-to-one correspondence between Tsallis and Rényi entropies of the same positive order, similar to the transition from ([36], Theorem 1) to ([36], Theorem 2), also Theorem 8 enables to strengthen the Schur-concavity property of the Rényi entropy. For information-theoretic implications of the Schur-concavity of the Rényi entropy, the reader is referred to, e.g., [34], ([41], Theorem 3) and ([42], Theorem 11).*

## 3. Illustration of the Main Results and Implications

### 3.1. Illustration of Theorems 2 and 4

We apply here the data-processing inequalities in Theorems 2 and 4 to the new class of *f*-divergences introduced in Theorem 5.

In the setup of Theorems 2 and 4, consider communication over a time-varying binary-symmetric channel (BSC). Consequently, let X=Y={0,1}, and let

(106) $$\begin{array}{cc} \; & P_{X_{i}}\left(1\right)=p_{i},\;Q_{X_{i}}\left(1\right)=q_{i}, \end{array}$$

with $p_{i}\in \left(0,1\right)$ and $q_{i}\in \left(0,1\right)$ for every i∈{1,…,n}. Let the transition probabilities $P_{Y_{i}|X_{i}}\left(\cdot |\cdot \right)$ correspond to $\mathrm{BSC}(\delta_{i})$ (i.e., a BSC with a crossover probability $\delta_{i}$), i.e.,

(107) $$P_{Y_{i}|X_{i}}\left(y|x\right)=\{\begin{array}{cc} 1-\delta_{i} & \mathrm{if}\;x=y,\\ \delta_{i} & \mathrm{if}\;x\neq y. \end{array}$$

For all λ∈[0,1] and $\underline{x}\in X^{n}$, the probability mass function at the channel input is given by

(108) $$\begin{array}{cc} \; & R_{X^{n}}^{\left(\lambda \right)}\left(\underline{x}\right)=\prod_{i=1}^{n}R_{X_{i}}^{\left(\lambda \right)}\left(x_{i}\right), \end{array}$$

with

(109) $$\begin{array}{c} R_{X_{i}}^{\left(\lambda \right)}\left(x\right)=\lambda P_{X_{i}}\left(x\right)+\left(1-\lambda \right)Q_{X_{i}}\left(x\right),\;x\in \left\{0,1\right\}, \end{array}$$

where the probability mass function in (109) refers to a Bernoulli distribution with parameter $\lambda p_{i}+\left(1-\lambda \right)q_{i}$. At the output of the time-varying BSC (see (42)–(44) and (107)), for all $\underline{y}\in Y^{n}$,

(110) $$\begin{array}{cc} \; & R_{Y^{n}}^{\left(\lambda \right)}\left(\underline{y}\right)=\prod_{i=1}^{n}R_{Y_{i}}^{\left(\lambda \right)}\left(y_{i}\right),\;P_{Y^{n}}\left(\underline{y}\right)=\prod_{i=1}^{n}P_{Y_{i}}\left(y_{i}\right),\;Q_{Y^{n}}\left(\underline{y}\right)=\prod_{i=1}^{n}Q_{Y_{i}}\left(y_{i}\right), \end{array}$$

where

(111) $$\begin{array}{cc} \; & R_{Y_{i}}^{\left(\lambda \right)}\left(1\right)=\left(\lambda p_{i}+\left(1-\lambda \right)q_{i}\right)*\delta_{i}, \end{array}$$

(112) $$\begin{array}{cc} \; & P_{Y_{i}}\left(1\right)=p_{i}*\delta_{i}, \end{array}$$

(113) $$\begin{array}{cc} \; & Q_{Y_{i}}\left(1\right)=q_{i}*\delta_{i}, \end{array}$$

with

(114) $$\begin{array}{c} a*b:=a(1-b)+(1-a)b,\;0\leq a,b\leq 1. \end{array}$$

The $\chi^{2}$-divergence from Bernoulli(p) to Bernoulli(q) is given by

(115) $$\begin{array}{c} \chi^{2}\left(\mathrm{Bernoulli}(p)\;∥\;\mathrm{Bernoulli}(q)\right)=\frac{{\left(p-q\right)}^{2}}{q(1-q)}, \end{array}$$

and since the probability mass functions $P_{X_{i}}$, $Q_{X_{i}}$, $P_{Y_{i}}$ and $Q_{Y_{i}}$ correspond to Bernoulli distributions with parameters $p_{i}$, $q_{i}$, $p_{i}*\delta_{i}$ and $q_{i}*\delta_{i}$, respectively, Theorem 2 gives that

$$\begin{array}{cc} \; & \;c_{f_{\alpha }}\left(\xi_{1}\left(n,\lambda \right),\;\xi_{2}\left(n,\lambda \right)\right)\;\left[\;\prod_{i=1}^{n}\left(1+\frac{\lambda^{2}{\left(p_{i}-q_{i}\right)}^{2}}{q_{i}\left(1-q_{i}\right)}\right)-\prod_{i=1}^{n}\left(1+\frac{\lambda^{2}{\left(p_{i}*\delta_{i}-q_{i}*\delta_{i}\right)}^{2}}{\left(q_{i}*\delta_{i}\right)\left(1-q_{i}*\delta_{i}\right)}\right)\right] \end{array}$$

(116) $$\begin{array}{cc} \; & \;\leq D_{f_{\alpha }}\left(R_{X^{n}}^{\left(\lambda \right)}\;∥\;Q_{X^{n}}\right)-D_{f_{\alpha }}\left(R_{Y^{n}}^{\left(\lambda \right)}\;∥\;Q_{Y^{n}}\right) \end{array}$$

(117) $$\begin{array}{cc} \; & \;\leq e_{f_{\alpha }}\left(\xi_{1}\left(n,\lambda \right),\;\xi_{2}\left(n,\lambda \right)\right)\left[\;\prod_{i=1}^{n}\left(1+\frac{\lambda^{2}{\left(p_{i}-q_{i}\right)}^{2}}{q_{i}\left(1-q_{i}\right)}\right)-\prod_{i=1}^{n}\left(1+\frac{\lambda^{2}{\left(p_{i}*\delta_{i}-q_{i}*\delta_{i}\right)}^{2}}{\left(q_{i}*\delta_{i}\right)\left(1-q_{i}*\delta_{i}\right)}\right)\right] \end{array}$$

for all λ∈[0,1] and n∈N. From (26), (31) and (55), we get that for all $\xi_{1}<1<\xi_{2}$,

(118) $$\begin{array}{cc} c_{f_{\alpha }}\left(\xi_{1},\xi_{2}\right) & =\frac{1}{2}\;\underset{t\in [\xi_{1},\xi_{2}]}{\inf}f_{\alpha }^{\prime \prime }\left(t\right) \end{array}$$

(119) $$\begin{array}{cc} \; & =\log\left(\alpha +\xi_{1}\right)+\frac{3}{2}\;\log e, \end{array}$$

(120) $$\begin{array}{cc} e_{f_{\alpha }}\left(\xi_{1},\xi_{2}\right) & =\frac{1}{2}\;\underset{t\in [\xi_{1},\xi_{2}]}{\sup}f_{\alpha }^{\prime \prime }\left(t\right) \end{array}$$

(121) $$\begin{array}{cc} \; & =\log\left(\alpha +\xi_{2}\right)+\frac{3}{2}\;\log e, \end{array}$$

and, from (47), (48) and (106), for all λ∈(0,1],

(122) $$\begin{array}{cc} \; & \xi_{1}\left(n,\lambda \right):=\prod_{i=1}^{n}\left(1-\lambda +\lambda \;\min\left\{\frac{p_{i}}{q_{i}},\frac{1-p_{i}}{1-q_{i}}\right\}\right)\in \left[0,1\right), \end{array}$$

(123) $$\begin{array}{cc} \; & \xi_{2}\left(n,\lambda \right):=\prod_{i=1}^{n}\left(1-\lambda +\lambda \;\max\left\{\frac{p_{i}}{q_{i}},\frac{1-p_{i}}{1-q_{i}}\right\}\right)\in \left(1,\infty \right), \end{array}$$

provided that $p_{i}\neq q_{i}$ for some i∈{1,…,n} (otherwise, both *f*-divergences in the right side of (116) are equal to zero since $P_{X_{i}}\equiv Q_{X_{i}}$ and therefore $R_{X_{i}}^{\left(\lambda \right)}\equiv Q_{X_{i}}$ for all *i* and λ∈[0,1]). Furthermore, from Item (c) of Theorem 2, for every n∈N and $\alpha \geq e^{-\frac{3}{2}}$,

(124) $$\begin{array}{c} \lim_{\lambda \to 0^{+}}\frac{D_{f_{\alpha }}\left(R_{X^{n}}^{\left(\lambda \right)}\;∥\;Q_{X^{n}}\right)-D_{f_{\alpha }}\left(R_{Y^{n}}^{\left(\lambda \right)}\;∥\;Q_{Y^{n}}\right)}{\lambda^{2}}\\ =\left(\log\left(\alpha +1\right)+\frac{3}{2}\;\log e\right)\sum_{i=1}^{n}\left\{\frac{{\left(p_{i}-q_{i}\right)}^{2}}{q_{i}\left(1-q_{i}\right)}-\frac{{\left(p_{i}*\delta_{i}-q_{i}*\delta_{i}\right)}^{2}}{\left(q_{i}*\delta_{i}\right)\left(1-q_{i}*\delta_{i}\right)}\right\}, \end{array}$$

and the lower and upper bounds in the left side of (116) and the right side of (117), respectively, are tight as we let λ→0, and they both coincide with the limit in the right side of (124).

**Figure 1:**
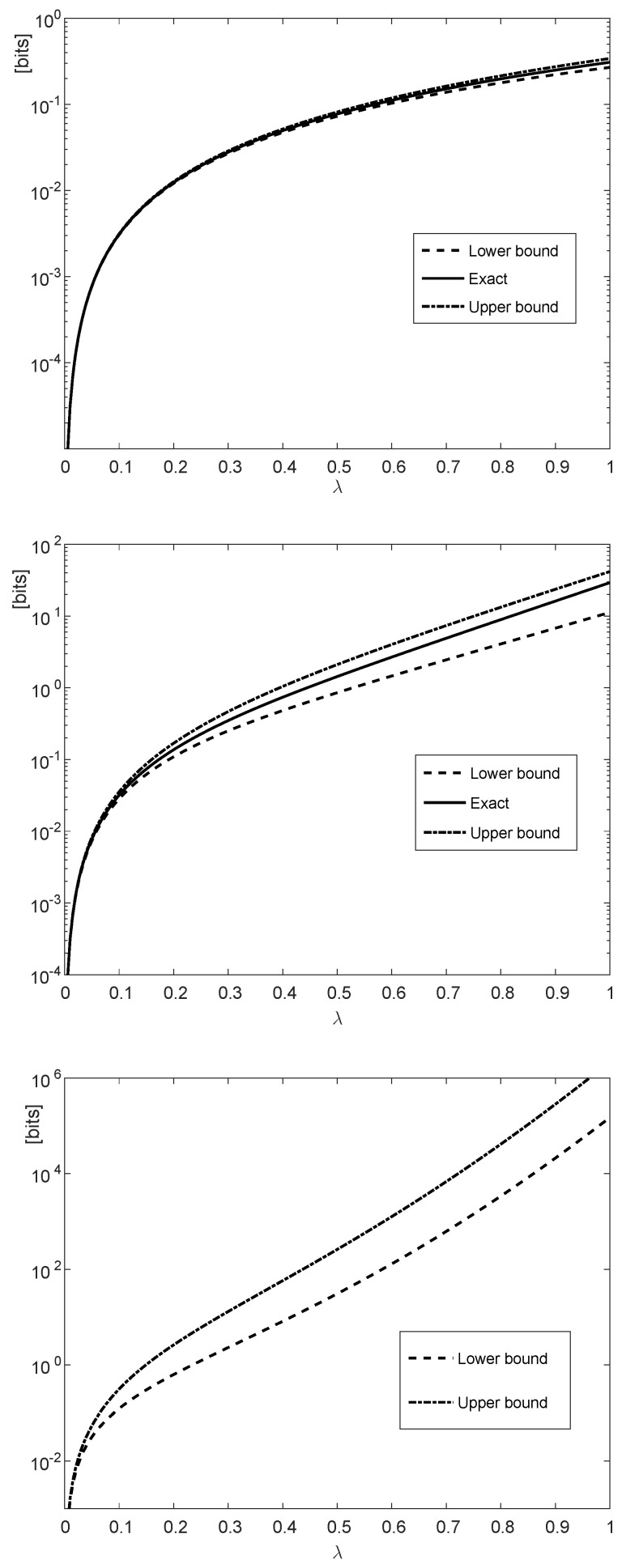
The bounds in Theorem 2 applied to $D_{f_{\alpha }}\left(R_{X^{n}}^{\left(\lambda \right)}\;∥\;Q_{X^{n}}\right)-D_{f_{\alpha }}\left(R_{Y^{n}}^{\left(\lambda \right)}\;∥\;Q_{Y^{n}}\right)$ (vertical axis) versus λ∈[0,1] (horizontal axis). The $f_{\alpha }$-divergence refers to Theorem 5. The probability mass functions $P_{X^{n}}$ and $Q_{X^{n}}$ correspond, respectively, to discrete memoryless sources emitting *n* i.i.d. Bernoulli(p) and Bernoulli(q) symbols; the symbols are transmitted over BSC(δ) with $\left(\alpha ,p,q,\delta \right)=\left(1,\frac{1}{4},\frac{1}{2},0.110\right)$. The bounds in the upper and middle plots are compared to the exact values, being computationally feasible for n=1 and n=10, respectively. The upper, middle and lower plots correspond, respectively, to n=1, n=10, and n=50.

Figure 1 illustrates the upper and lower bounds in (116) and (117) with α=1, $p_{i}\equiv \frac{1}{4}$, $q_{i}\equiv \frac{1}{2}$ and $\delta_{i}\equiv 0.110$ for all *i*, and n∈{1,10,50}. In the special case where $\left\{\delta_{i}\right\}$ are fixed for all *i*, the communication channel is a time-invariant BSC whose capacity is equal to $\frac{1}{2}$ bit per channel use.

By referring to the upper and middle plots of Figure 1, if n=1 or n=10, then the exact values of the differences of the $f_{\alpha }$-divergences in the right side of (116) are calculated numerically, being compared to the lower and upper bounds in the left side of (116) and the right side of (117) respectively. Since the $f_{\alpha }$-divergence does not tensorize, the computation of the exact value of each of the two $f_{\alpha }$-divergences in the right side of (116) involves a pre-computation of $2^{n}$ probabilities for each of the probability mass functions $P_{X^{n}}$, $Q_{X^{n}}$, $P_{Y^{n}}$ and $Q_{Y^{n}}$; this computation is prohibitively complex unless *n* is small enough.

We now apply the bound in Theorem 4. In view of (51), (54), (55) and (73), for all λ∈(0,1] and $\alpha \geq e^{-\frac{3}{2}}$,

$$\begin{array}{cc} \; & \frac{D_{f_{\alpha }}\left(R_{Y^{n}}^{\left(\lambda \right)}\;∥\;Q_{Y^{n}}\right)}{D_{f_{\alpha }}\left(R_{X^{n}}^{\left(\lambda \right)}\;∥\;Q_{X^{n}}\right)} \end{array}$$

(125) $$\begin{array}{cc} \; & \leq \frac{\kappa_{\alpha }\left(\xi_{1}\left(n,\lambda \right),\;\xi_{2}\left(n,\lambda \right)\right)}{f_{\alpha }\left(0\right)+f_{\alpha }^{\prime }\left(1\right)}\;\frac{\prod_{i=1}^{n}\left(1+\lambda^{2}\;\chi^{2}(P_{Y_{i}}\;∥\;Q_{Y_{i}}\right))-1}{\prod_{i=1}^{n}\left(1+\lambda^{2}\;\chi^{2}(P_{X_{i}}\;∥\;Q_{X_{i}}\right))-1} \end{array}$$

(126) $$\begin{array}{cc} \; & =\frac{f_{\alpha }\left(\xi_{2}\left(n,\lambda \right)\right)+f_{\alpha }^{\prime }\left(1\right)\;\left(1-\xi_{2}\left(n,\lambda \right)\right)}{{\left(\xi_{2}\left(n,\lambda \right)-1\right)}^{2}\;\left(f_{\alpha }\left(0\right)+f_{\alpha }^{\prime }\left(1\right)\right)}\cdot \frac{\prod_{i=1}^{n}\left(1+\frac{\lambda^{2}{\left(p_{i}*\delta_{i}-q_{i}*\delta_{i}\right)}^{2}}{\left(q_{i}*\delta_{i}\right)\left(1-q_{i}*\delta_{i}\right)}\right)-1}{\prod_{i=1}^{n}\left(1+\frac{\lambda^{2}{\left(p_{i}-q_{i}\right)}^{2}}{q_{i}\left(1-q_{i}\right)}\right)-1}, \end{array}$$

where $\xi_{1}\left(n,\lambda \right)\in \left[0,1\right)$ and $\xi_{2}\left(n,\lambda \right)\in \left(1,\infty \right)$ are given in (122) and (123), respectively, and for t≥0,

(127) $$\begin{array}{c} f_{\alpha }\left(t\right)+f_{\alpha }^{\prime }\left(1\right)\left(1-t\right)\\ ={\left(\alpha +t\right)}^{2}\log\left(\alpha +t\right)-{\left(\alpha +1\right)}^{2}\log\left(\alpha +1\right)+\left[2(\alpha +1)\log(\alpha +1)+(\alpha +1)\log e\right]\left(1-t\right). \end{array}$$

**Figure 2:**
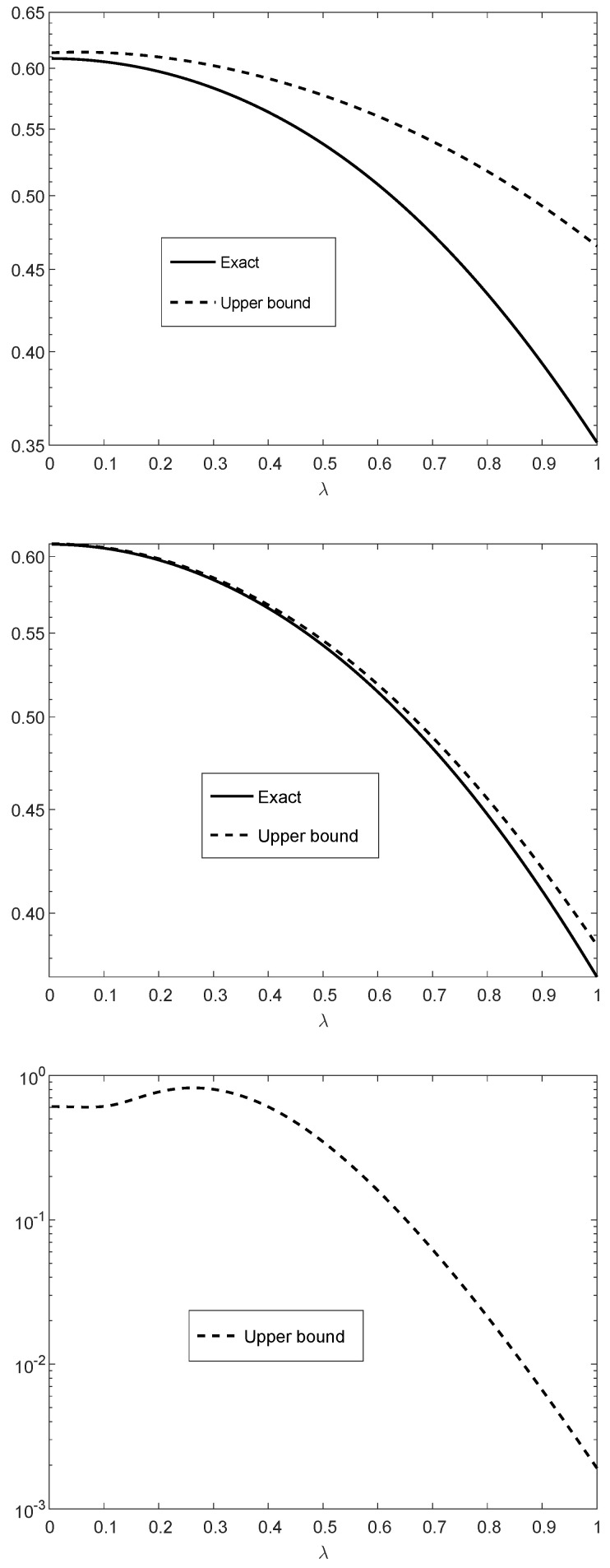
The upper bound in Theorem 4 applied to $\frac{D_{f_{\alpha }}\left(R_{Y^{n}}^{\left(\lambda \right)}\;∥\;Q_{Y^{n}}\right)}{D_{f_{\alpha }}\left(R_{X^{n}}^{\left(\lambda \right)}\;∥\;Q_{X^{n}}\right)}$ (see (125)–(127)) in the vertical axis versus λ∈[0,1] in the horizontal axis. The $f_{\alpha }$-divergence refers to Theorem 5. The probability mass functions $P_{X_{i}}$ and $Q_{X_{i}}$ are Bernoulli(p) and Bernoulli(q), respectively, for all i∈{1,…,n} with *n* uses of BSC(δ), and parameters $\left(p,q,\delta \right)=\left(\frac{1}{4},\frac{1}{2},0.110\right)$. The upper and middle plots correspond to n=10 with α=10 and α=100, respectively; the middle and lower plots correspond to α=100 with n=10 and n=100, respectively. The bounds in the upper and middle plots are compared to the exact values, being computationally feasible for n=10.

Figure 2 illustrates the upper bound on $\frac{D_{f_{\alpha }}\left(R_{Y^{n}}^{\left(\lambda \right)}\;∥\;Q_{Y^{n}}\right)}{D_{f_{\alpha }}\left(R_{X^{n}}^{\left(\lambda \right)}\;∥\;Q_{X^{n}}\right)}$ (see (125)–(127)) as a function of λ∈(0,1]. It refers to the case where $p_{i}\equiv \frac{1}{4}$, $q_{i}\equiv \frac{1}{2}$, and $\delta_{i}\equiv 0.110$ for all *i* (similarly to Figure 1). The upper and middle plots correspond to n=10 with α=10 and α=100, respectively; the middle and lower plots correspond to α=100 with n=10 and n=100, respectively. The bounds in the upper and middle plots are compared to their exact values since their numerical computations are feasible for n=10. It is observed from the numerical comparisons for n=10 (see the upper and middle plots in Figure 2) that the upper bounds are informative, especially for large values of α where the $f_{\alpha }$-divergence becomes closer to a scaled version of the $\chi^{2}$-divergence (see Item (e) in Theorem 5).

### 3.2. Illustration of Theorems 3 and 5

Following the application of the data-processing inequalities in Theorems 2 and 4 to a class of *f*-divergences (see Section 3.1), some interesting properties of this class are introduced in Theorem 5.

For $\alpha \geq e^{-\frac{3}{2}}$, let $d_{f_{\alpha }}:{\left(0,1\right)}^{2}\to \left[0,\infty \right)$ be the binary $f_{\alpha }$-divergence (see (55)), defined as

(128) $$\begin{array}{cc} d_{f_{\alpha }}\left(p∥q\right) & :=D_{f_{\alpha }}\left(\;\mathrm{Bernoulli}(p)\;∥\;\mathrm{Bernoulli}(q)\;\right)\\ & \;=q{\left(\alpha +\frac{p}{q}\right)}^{2}\log\left(\alpha +\frac{p}{q}\right)+\left(1-q\right){\left(\alpha +\frac{1-p}{1-q}\right)}^{2}\log\left(\alpha +\frac{1-p}{1-q}\right) \end{array}$$

(129) $$\begin{array}{cc} \; & \;-{\left(\alpha +1\right)}^{2}\log\left(\alpha +1\right),\;\forall \;\left(p,q\right)\in {\left(0,1\right)}^{2}. \end{array}$$

**Figure 3:**
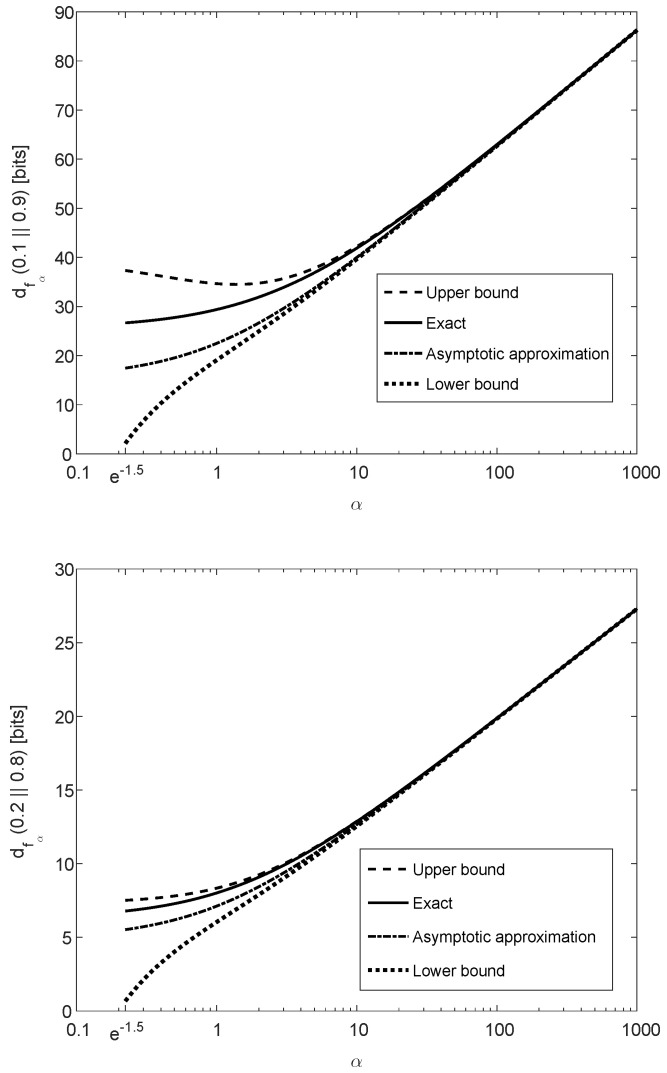
Plots of $d_{f_{\alpha }}\left(p∥q\right)$, its upper and lower bounds in (61) and (65), respectively, and its asymptotic approximation in (66) for large values of α. The plots are shown as a function of $\alpha \in \left[e^{-\frac{3}{2}},1000\right]$. The upper and lower plots refer, respectively, to (p,q)=(0.1,0.9) and (p,q)=(0.2,0.8).

Theorem 5 is illustrated in Figure 3, showing that $d_{f_{\alpha }}\left(p∥q\right)$ is monotonically increasing as a function of $\alpha \geq e^{-\frac{3}{2}}$ (note that the concavity in α is not reflected from these plots because the horizontal axis of α is in logarithmic scaling). The binary divergence $d_{f_{\alpha }}\left(p∥q\right)$ is also compared in Figure 3 with its lower and upper bounds in (61) and (65), respectively, illustrating that these bounds are both asymptotically tight for large values of α. The asymptotic approximation of $d_{f_{\alpha }}\left(p∥q\right)$ for large α, expressed as a function of α and $\chi^{2}\left(p∥q\right)$ (see (66)), is also depicted in Figure 3. The upper and lower plots in Figure 3 refer, respectively, to (p,q)=(0.1,0.9) and (0.2,0.8); a comparison of these plots show a better match between the exact value of the binary divergence, its upper and lower bounds, and its asymptotic approximation when the values of *p* and *q* are getting closer.

In view of the results in (66) and (68), it is interesting to note that the asymptotic value of $D_{f_{\alpha }}\left(P∥Q\right)$ for large values of α is also the exact scaling of this *f*-divergence for *any finite* value of $\alpha \geq e^{-\frac{3}{2}}$ when the probability mass functions *P* and *Q* are close enough to each other.

We next consider the ratio of the contraction coefficients $\frac{\mu_{f_{\alpha }}\left(Q_{X},W_{Y|X}\right)}{\mu_{\chi^{2}}\left(Q_{X},W_{Y|X}\right)}$ where $Q_{X}$ is finitely supported on X and it is not a point mass (i.e., |X|≥2), and $W_{Y|X}$ is arbitrary. For all $\alpha \geq e^{-\frac{3}{2}}$,

(130) $$\begin{array}{c} 1\leq \frac{\mu_{f_{\alpha }}\left(Q_{X},W_{Y|X}\right)}{\mu_{\chi^{2}}\left(Q_{X},W_{Y|X}\right)}\leq \frac{f_{\alpha }\left(\xi \right)+f_{\alpha }^{\prime }\left(1\right)\left(1-\xi \right)}{{\left(\xi -1\right)}^{2}\left(f_{\alpha }\left(0\right)+f_{\alpha }^{\prime }\left(1\right)\right)}, \end{array}$$

where $f_{\alpha }:\left(0,\infty \right)\to R$ is given in (55), and

(131) $$\begin{array}{c} \xi :=\frac{1}{\min_{x\in X}\;Q_{X}\left(x\right)}\in \left[|X|,\infty \right). \end{array}$$

The left-side inequality in (130) is due to ([25], Theorem 2) (see Proposition 2), and the right-side inequality in (130) holds due to (53) and (73).

**Figure 4:**
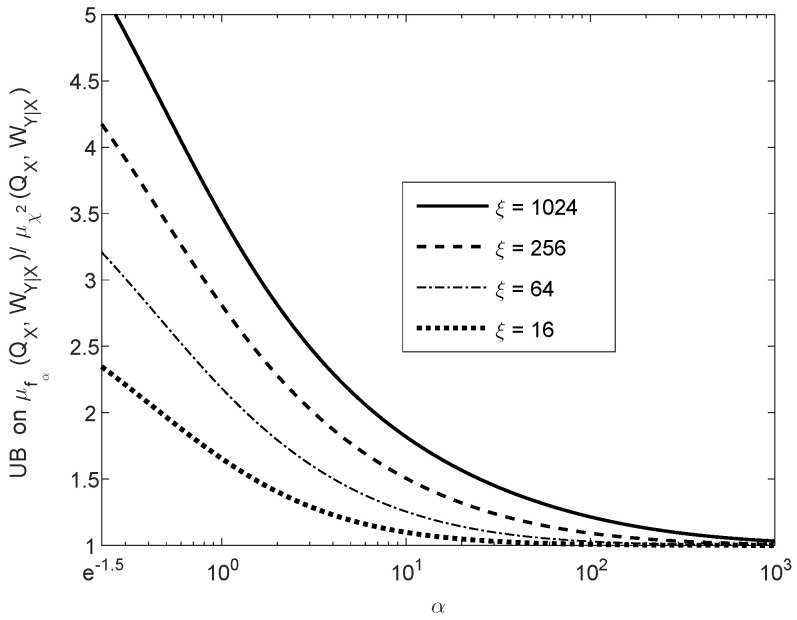
Curves of the upper bound on the ratio of contraction coefficients $\frac{\mu_{f_{\alpha }}\left(Q_{X},W_{Y|X}\right)}{\mu_{\chi^{2}}\left(Q_{X},W_{Y|X}\right)}$ (see the right-side inequality of (130)) as a function of the parameter $\alpha \geq e^{-\frac{3}{2}}$. The curves correspond to different values of ξ in (131).

Figure 4 shows the upper bound on the ratio of contraction coefficients $\frac{\mu_{f_{\alpha }}\left(Q_{X},W_{Y|X}\right)}{\mu_{\chi^{2}}\left(Q_{X},W_{Y|X}\right)}$, as it is given in the right-side inequality of (130), as a function of the parameter $\alpha \geq e^{-\frac{3}{2}}$. The curves in Figure 4 correspond to different values of ξ∈[|X|,∞), as it is given in (131); these upper bounds are monotonically decreasing in α, and they asymptotically tend to 1 as we let α→∞. Hence, in view of the left-side inequality in (130), the upper bound on the ratio of the contraction coefficients (in the right-side inequality) is asymptotically tight in α. The fact that the ratio of the contraction coefficients in the middle of (130) tends asymptotically to 1, as α gets large, is not directly implied by Item (e) of Theorem 5. The latter implies that, for fixed probability mass functions *P* and *Q* and for sufficiently large α,

(132) $$\begin{array}{c} D_{f_{\alpha }}\left(P∥Q\right)\approx \left[\log\left(\alpha +1\right)+\frac{3}{2}\log e\right]\;\chi^{2}\left(P∥Q\right); \end{array}$$

however, there is no guarantee that for fixed *Q* and sufficiently large α, the approximation in (132) holds for all *P*. By the upper bound in the right side of (130), it follows however that $\mu_{f_{\alpha }}\left(Q_{X},W_{Y|X}\right)$ tends asymptotically (as we let α→∞) to the contraction coefficient of the $\chi^{2}$ divergence.

### 3.3. Illustration of Theorem 7 and Further Results

Theorem 7 provides upper and lower bounds on an *f*-divergence, $D_{f}\left(Q∥U_{n}\right)$, from any probability mass function *Q* supported on a finite set of cardinality *n* to an equiprobable distribution over this set. We apply in the following, the exact formula for

(133) $$\begin{array}{c} d_{f}\left(\rho \right):=\lim_{n\to \infty }\;\max_{Q\in P_{n}\left(\rho \right)}D_{f}\left(Q∥U_{n}\right),\;\rho \geq 1 \end{array}$$

to several important *f*-divergences. From (87),

(134) $$\begin{array}{c} d_{f}\left(\rho \right)=\max_{x\in [0,1]}\left\{xf\left(\frac{\rho }{1+(\rho -1)x}\right)+\left(1-x\right)f\left(\frac{1}{1+(\rho -1)x}\right)\right\},\;\rho \geq 1. \end{array}$$

Since *f* is a convex function on (0,∞) with f(1)=0, Jensen’s inequality implies that the function which is subject to maximization in the right-side of (134) is non-negative over the interval [0,1]. It is equal to zero at the endpoints of the interval [0,1], so the maximum over this interval is attained at an interior point. Note also that, in view of Items (d) and (e) of Theorem 7, the exact asymptotic expression in (134) satisfies

(135) $$\begin{array}{c} \max_{Q\in P_{n}\left(\rho \right)}D_{f}\left(Q∥U_{n}\right)\leq d_{f}\left(\rho \right),\;\forall \;n\in \left\{2,3,\ldots \right\},\;\rho \geq 1. \end{array}$$

#### 3.3.1. Total Variation Distance

This distance is an *f*-divergence with f(t):=|t−1| for t>0. Substituting *f* into (134) gives

(136) $$\begin{array}{c} d_{f}\left(\rho \right)=\max_{x\in [0,1]}\left\{\frac{2(\rho -1)x(1-x)}{1+(\rho -1)x}\right\}. \end{array}$$

By setting to zero the derivative of the function which is subject to maximization in the right side of (136), it can be verified that the maximizer over this interval is equal to $x=\frac{1}{1+\sqrt{\rho }}$, which implies that

(137) $$\begin{array}{c} d_{f}\left(\rho \right)=\frac{2(\sqrt{\rho }-1)}{\sqrt{\rho }+1},\;\forall \;\rho \geq 1. \end{array}$$

#### 3.3.2. Alpha Divergences

The class of Alpha divergences forms a parametric subclass of the *f*-divergences, which includes in particular the relative entropy, $\chi^{2}$-divergence, and the squared-Hellinger distance. For α∈R, let

(138) $$\begin{array}{c} D_{A}^{\left(\alpha \right)}\left(P∥Q\right):=D_{u_{\alpha }}\left(P∥Q\right), \end{array}$$

where $u_{\alpha }:\left(0,\infty \right)\to R$ is a non-negative and convex function with $u_{\alpha }\left(1\right)=0$, which is defined for t>0 as follows (see ([8], Chapter 2), followed by studies in, e.g., [10,16,43,44,45]):

(139) $$u_{\alpha }\left(t\right):=\{\begin{array}{cc} \frac{t^{\alpha }-\alpha \left(t-1\right)-1}{\alpha (\alpha -1)}, & \alpha \in (-\infty ,0)\cup (0,1)\cup (1,\infty ),\\ t\log_{e}t+1-t, & \alpha =1,\\ -\log_{e}t, & \alpha =0. \end{array}$$

The functions $u_{0}$ and $u_{1}$ are defined in the right side of (139) by a continuous extension of $u_{\alpha }$ at α=0 and α=1, respectively. The following relations hold (see, e.g., ([44], (10)–(13))):

(140) $$\begin{array}{c} D_{A}^{\left(1\right)}\left(P∥Q\right)=\frac{1}{\log e}\;D\left(P∥Q\right), \end{array}$$

(141) $$\begin{array}{c} D_{A}^{\left(0\right)}\left(P∥Q\right)=\frac{1}{\log e}\;D\left(Q∥P\right), \end{array}$$

(142) $$\begin{array}{c} D_{A}^{\left(2\right)}\left(P∥Q\right)=\frac{1}{2}\;\chi^{2}\left(P∥Q\right), \end{array}$$

(143) $$\begin{array}{c} D_{A}^{\left(-1\right)}\left(P∥Q\right)=\frac{1}{2}\;\chi^{2}\left(Q∥P\right), \end{array}$$

(144) $$\begin{array}{c} D_{A}^{\left(\frac{1}{2}\right)}\left(P∥Q\right)=4H^{2}\left(P∥Q\right). \end{array}$$

Substituting $f:=u_{\alpha }$ (see (139)) into the right side of (134) gives that

(145) $$\begin{array}{cc} \Delta (\alpha ,\rho ) & :=d_{u_{\alpha }}\left(\rho \right) \end{array}$$

(146) $$\begin{array}{cc} \; & \;=\lim_{n\to \infty }\max_{Q\in P_{n}\left(\rho \right)}D_{A}^{\left(\alpha \right)}\left(Q∥U_{n}\right) \end{array}$$

(147) $$\begin{array}{cc} \; & \;=\max_{x\in [0,1]}\left\{\frac{1+(\rho^{\alpha }-1)x}{{\left(1+(\rho -1)x\right)}^{\alpha }}-1\right\}. \end{array}$$

Setting to zero the derivative of the function which is subject to maximization in the right side of (147) gives

(148) $$\begin{array}{c} x=x^{*}:=\frac{1+\alpha \left(\rho -1\right)-\rho^{\alpha }}{\left(1-\alpha \right)\left(\rho -1\right)\left(\rho^{\alpha }-1\right)}, \end{array}$$

where it can be verified that $x^{*}\in \left(0,1\right)$ for all α∈(−∞,0)∪(0,1)∪(1,∞) and ρ>1. Substituting (148) into the right side of (147) gives that, for all such α and ρ,

(149) $$\begin{array}{c} \Delta \left(\alpha ,\rho \right)=\frac{1}{\alpha (\alpha -1)}\left[\frac{{\left(1-\alpha \right)}^{\alpha -1}{\left(\rho^{\alpha }-1\right)}^{\alpha }{\left(\rho -\rho^{\alpha }\right)}^{1-\alpha }}{\left(\rho -1\right)\alpha^{\alpha }}-1\right]. \end{array}$$

By a continuous extension of Δ(α,ρ) in (149) at α=1 and α=0, it follows that for all ρ>1

(150) $$\begin{array}{c} \Delta \left(1,\rho \right)=\Delta \left(0,\rho \right)=\frac{\rho \log\rho }{\rho -1}-\log\left(\frac{e\rho \log_{e}\rho }{\rho -1}\right). \end{array}$$

Consequently, for all ρ>1,

(151) $$\begin{array}{cc} \lim_{n\to \infty }\max_{Q\in P_{n}\left(\rho \right)}D\left(Q∥U_{n}\right)\; & =\log e\;\lim_{n\to \infty }\max_{Q\in P_{n}\left(\rho \right)}D_{A}^{\left(1\right)}\left(Q∥U_{n}\right) \end{array}$$

(152) $$\begin{array}{cc} \; & =\Delta (1,\rho )\;\log e \end{array}$$

(153) $$\begin{array}{cc} \; & =\frac{\rho \log\rho }{\rho -1}-\log\left(\frac{e\rho \log_{e}\rho }{\rho -1}\right), \end{array}$$

where (151) holds due to (140); (152) is due to (146), and (153) holds due to (150). This sharpens the result in ([33], Theorem 2) for the relative entropy from the equiprobable distribution, $D\left(Q∥U_{n}\right)=\log n-H\left(Q\right)$, by showing that the bound in ([33], (7)) is asymptotically tight as we let n→∞. The result in ([33], Theorem 2) can be further tightened for finite *n* by applying the result in Theorem 7 (d) with $f\left(t\right):=u_{1}\left(t\right)\;\log e=t\log t+\left(1-t\right)\log e$ for all t>0 (although, unlike the asymptotic result in (149), the refined bound for a finite *n* does not lend itself to a closed-form expression as a function of *n*; see also ([34], Remark 3), which provides such a refinement of the bound on $D(Q∥U_{n})$ for finite *n* in a different approach).

From (141), (146) and (150), it follows similarly to (153) that for all ρ>1

(154) $$\begin{array}{cc} \lim_{n\to \infty }\max_{Q\in P_{n}\left(\rho \right)}D\left(U_{n}∥Q\right)\; & =\Delta (0,\rho )\;\log e \end{array}$$

(155) $$\begin{array}{cc} \; & =\frac{\rho \log\rho }{\rho -1}-\log\left(\frac{e\rho \log_{e}\rho }{\rho -1}\right). \end{array}$$

It should be noted that in view of the one-to-one correspondence between the Rényi divergence and the Alpha divergence of the same order α where, for α≠1,

(156) $$\begin{array}{c} D_{\alpha }\left(P∥Q\right)=\frac{1}{\alpha -1}\;\log\left(1+\alpha \left(\alpha -1\right)D_{A}^{\left(\alpha \right)}\left(P∥Q\right)\right), \end{array}$$

the asymptotic result in (149) can be obtained from ([34], Lemma 4) and vice versa; however, in [34], the focus is on the Rényi divergence from the equiprobable distribution, whereas the result in (149) is obtained by specializing the asymptotic expression in (134) for a general *f*-divergence. Note also that the result in ([34], Lemma 4) is restricted to α>0, whereas the result in (149) and (150) covers all values of α∈R.

In view of (146), (149), (153), (155), and the special cases of the Alpha divergences in (140)–(144), it follows that for all ρ>1 and for every integer n≥2

(157) $$\begin{array}{cc} \max_{Q\in P_{n}\left(\rho \right)}D\left(Q∥U_{n}\right)\; & \leq \Delta \left(1,\rho \right)\;\log e=\frac{\rho \log\rho }{\rho -1}-\log\left(\frac{e\rho \log_{e}\rho }{\rho -1}\right), \end{array}$$

(158) $$\begin{array}{cc} \max_{Q\in P_{n}\left(\rho \right)}D\left(U_{n}∥Q\right)\; & \leq \Delta \left(0,\rho \right)\;\log e=\frac{\rho \log\rho }{\rho -1}-\log\left(\frac{e\rho \log_{e}\rho }{\rho -1}\right), \end{array}$$

(159) $$\begin{array}{cc} \max_{Q\in P_{n}\left(\rho \right)}\chi^{2}\left(Q∥U_{n}\right) & \leq 2\Delta \left(2,\rho \right)=\frac{{\left(\rho -1\right)}^{2}}{4\rho }, \end{array}$$

(160) $$\begin{array}{cc} \max_{Q\in P_{n}\left(\rho \right)}\chi^{2}\left(U_{n}∥Q\right) & \leq 2\Delta \left(-1,\rho \right)=\frac{{\left(\rho -1\right)}^{2}}{4\rho }, \end{array}$$

(161) $$\begin{array}{cc} \max_{Q\in P_{n}\left(\rho \right)}H^{2}\left(Q∥U_{n}\right)\; & \leq \frac{1}{4}\;\Delta \left(\frac{1}{2},\rho \right)=\frac{{\left(\sqrt[{4}]{\rho }-1\right)}^{2}}{\sqrt{\rho }+1}, \end{array}$$

and the upper bounds on the right sides of (157)–(161) are asymptotically tight in the limit where *n* tends to infinity.

The next result characterizes the function Δ:(0,∞)×(1,∞)→R as it is given in (149) and (150).

**Theorem** **9.** *The function* Δ *satisfies the following properties:*

*(a)*  For every ρ>1, Δ(α,ρ) is a convex function of α over the real line, and it is symmetric around $\alpha =\frac{1}{2}$ with a global minimum at $\alpha =\frac{1}{2}$.
*(b)*  *The following inequalities hold:*
  (162) $$\begin{array}{cc} \; & \alpha \;\Delta (\alpha ,\rho )\leq \beta \;\Delta (\beta ,\rho ),\;0<\alpha \leq \beta <\infty , \end{array}$$
  (163) $$\begin{array}{cc} \; & (1-\beta )\;\Delta (\beta ,\rho )\leq (1-\alpha )\;\Delta (\alpha ,\rho ),\;-\infty <\alpha \leq \beta <1. \end{array}$$
*(c)*  For every α∈R, Δ(α,ρ) is monotonically increasing and continuous in ρ∈(1,∞), and $\lim_{\rho \to 1^{+}}\Delta \left(\alpha ,\rho \right)=0$.

**Proof.**  See Appendix H.1.  □

**Remark** **6.** *The symmetry of Δ(α,ρ) around $\alpha =\frac{1}{2}$ (see Theorem 9 (a)) is not implied by the following symmetry property of the Alpha divergence around $\alpha =\frac{1}{2}$ (see, e.g., ([8], p. 36)):*

(164) $$\begin{array}{c} D_{A}^{\left(\frac{1}{2}+\alpha \right)}\left(P∥Q\right)=D_{A}^{\left(\frac{1}{2}-\alpha \right)}\left(Q∥P\right). \end{array}$$

Relying on Theorem 9, the following corollary gives a similar result to (146) where the order of *Q* and $U_{n}$ in $D_{A}^{\left(\alpha \right)}\left(\cdot ∥\cdot \right)$ is switched.

**Corollary** **1.** *For all α∈R and ρ>1,*

(165) $$\begin{array}{c} \lim_{n\to \infty }\max_{Q\in P_{n}\left(\rho \right)}D_{A}^{\left(\alpha \right)}\left(U_{n}∥Q\right)=\Delta \left(\alpha ,\rho \right). \end{array}$$

**Proof.**  See Appendix H.2.  □

We next further exemplify Theorem 7 for the relative entropy. Let f(t):=tlogt+(1−t)loge for t>0. Then, $f^{\prime \prime }\left(t\right)=\frac{\log e}{t}$, so the bounds on the second derivative of *f* over the interval $[\frac{1}{\rho },\rho ]$ are given by M=ρloge and $m=\frac{\log e}{\rho }$. Theorem 7 (h) gives the following bounds:

(166) $$\begin{array}{c} \frac{\left({n∥Q∥}_{2}^{2}-1\right)\log e}{2\rho }\leq D\left(Q∥U_{n}\right)\leq \frac{\rho \;\left({n∥Q∥}_{2}^{2}-1\right)\log e}{2}. \end{array}$$

From ([33], Theorem 2) (and (157)),

(167) $$\begin{array}{c} D\left(Q∥U_{n}\right)\leq \frac{\rho \log\rho }{\rho -1}-\log\left(\frac{e\rho \log_{e}\rho }{\rho -1}\right). \end{array}$$

Furthermore, (96) gives that

(168) $$\begin{array}{c} D\left(Q∥U_{n}\right)\leq \frac{1}{8}{\left(\rho -1\right)}^{2}\log e, \end{array}$$

which, for ρ>1, is a looser bound in comparison to (167). It can be verified, however, that the dominant term in the Taylor series expansion (around ρ=1) of the right side of (167) coincides with the right side of (168), so the bounds scale similarly for small values of ρ≥1.

Suppose that we wish to assert that, for every integer n≥2 and for all probability mass functions $Q\in P_{n}\left(\rho \right)$, the condition

(169) $$\begin{array}{c} D(Q∥U_{n})\leq d\log e \end{array}$$

holds with a fixed d>0. Due to the left side inequality in (89), this condition is equivalent to the requirement that

(170) $$\begin{array}{c} \lim_{n\to \infty }\max_{Q\in P_{n}\left(\rho \right)}D\left(Q∥U_{n}\right)\leq d\log e. \end{array}$$

Due to the asymptotic tightness of the upper bound in the right side of (157) (as we let n→∞), requiring that this upper bound is not larger than dloge is necessary and sufficient for the satisfiability of (169) for all *n* and $Q\in P_{n}\left(\rho \right)$. This leads to the analytical solution $\rho \leq \rho_{\max}^{\left(1\right)}\left(d\right)$ with (see Appendix I)

(171) $$\begin{array}{c} \rho_{\max}^{\left(1\right)}\left(d\right):=\frac{W_{-1}\left(-e^{-d-1}\right)}{W_{0}\left(-e^{-d-1}\right)}, \end{array}$$

where $W_{0}$ and $W_{-1}$ denote, respectively, the principal and secondary real branches of the Lambert *W* function [37]. Requiring the stronger condition where the right side of (168) is not larger than dloge leads to the sufficient solution $\rho \leq \rho_{\max}^{\left(2\right)}$ with the simple expression

(172) $$\begin{array}{c} \rho_{\max}^{\left(2\right)}\left(d\right):=1+\sqrt{8d}. \end{array}$$

**Figure 5:**
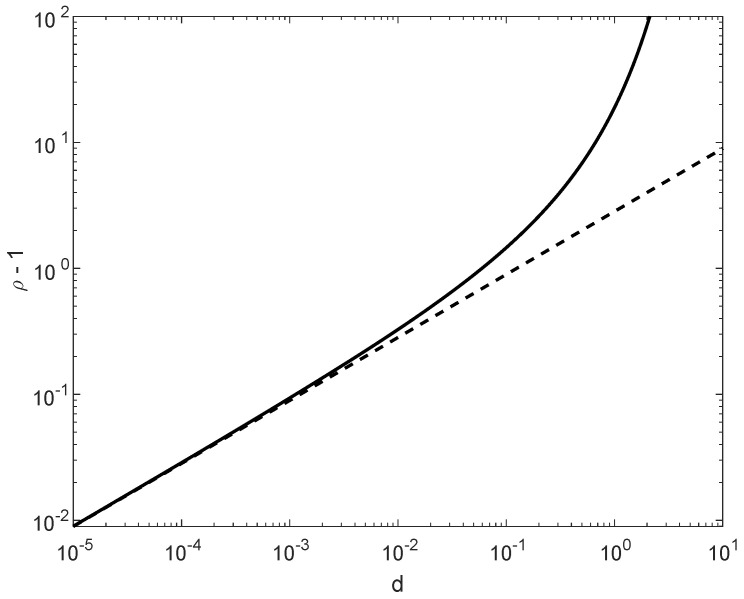
A comparison of the maximal values of ρ (minus 1) according to (171) and (172), asserting the satisfiability of the condition $D(Q∥U_{n})\leq d\log e$, with an arbitrary d>0, for all integers n≥2 and probability mass functions *Q* supported on {1,…,n} with $\frac{q_{\max}}{q_{\min}}\leq \rho$. The solid line refers to the necessary and sufficient condition which gives (171), and the dashed line refers to a stronger condition which gives (172).

In comparison to $\rho_{\max}^{\left(1\right)}$ in (171), $\rho_{\max}^{\left(2\right)}$ in (172) is more insightful; these values nearly coincide for small values of d>0, providing in that case the same range of possible values of ρ for asserting the satisfiability of condition (169). As it is shown in Figure 5, for d≤0.01, the difference between the maximal values of ρ in (171) and (172) is marginal, though in general $\rho_{\max}^{\left(1\right)}\left(d\right)>\rho_{\max}^{\left(2\right)}\left(d\right)$ for all d>0.

#### 3.3.3. The Subclass of *f*-Divergences in Theorem 5

This example refers to the subclass of *f*-divergences in Theorem 5. For these $f_{\alpha }$-divergences, with $\alpha \geq e^{-\frac{3}{2}}$, substituting $f:=f_{\alpha }$ from (55) into the right side of (134) gives that for all ρ≥1

(172) $$\begin{array}{cc} \Phi (\alpha ,\rho ) & :=d_{f_{\alpha }}\left(\rho \right) \end{array}$$

(173) $$\begin{array}{cc} \; & \;=\lim_{n\to \infty }\max_{Q\in P_{n}\left(\rho \right)}D_{f_{\alpha }}\left(Q∥U_{n}\right)\\ \; & \;=\max_{x\in [0,1]}\left\{x{\left(\alpha +\frac{\rho }{1+(\rho -1)x}\right)}^{2}\log\left(\alpha +\frac{\rho }{1+(\rho -1)x}\right)-{\left(\alpha +1\right)}^{2}\;\log\left(\alpha +1\right)\right. \end{array}$$

(175) $$\begin{array}{cc} \; & \;\left.+\;\left(1-x\right){\left(\alpha +\frac{1}{1+(\rho -1)x}\right)}^{2}\log\left(\alpha +\frac{1}{1+(\rho -1)x}\right)\right\}. \end{array}$$

The exact asymptotic expression in the right side of (175) is subject to numerical maximization.

We next provide two alternative closed-form upper bounds, based on Theorems 5 and 7, and study their tightness. The two upper bounds, for all $\alpha \geq e^{-\frac{3}{2}}$ and ρ≥1, are given by (see Appendix J)

(176) $$\begin{array}{cc} \Phi (\alpha ,\rho ) & \leq \left[\log\left(\alpha +1\right)+\frac{3}{2}\log e-\frac{\log e}{\alpha +1}\right]\frac{{\left(\rho -1\right)}^{2}}{4\rho }\\ & \;+\frac{\log e}{81(\alpha +1)}{\left(\frac{\left(\rho -1)(2\rho +1)(\rho +2\right)}{\rho (\rho +1)}\right)}^{2}, \end{array}$$

and

(177) $$\begin{array}{c} \Phi \left(\alpha ,\rho \right)\leq \left[\log\left(\alpha +\rho \right)+\frac{3}{2}\log e\right]\;\frac{{\left(\rho -1\right)}^{2}}{4\rho }. \end{array}$$

Suppose that we wish to assert that, for every integer n≥2 and for all probability mass functions $Q\in P_{n}\left(\rho \right)$, the condition

(178) $$\begin{array}{c} D_{f_{\alpha }}\left(Q∥U_{n}\right)\leq d\log e \end{array}$$

holds with a fixed d>0 and $\alpha \geq e^{-\frac{3}{2}}$. Due to (173)–(174) and the left side inequality in (89), the satisfiability of the latter condition is equivalent to the requirement that

(179) $$\begin{array}{c} \Phi (\alpha ,\rho )\leq d\log e. \end{array}$$

In order to obtain a sufficient condition for ρ to satisfy (179), expressed as an explicit function of α and *d*, the upper bound in the right side of (176) is slightly loosened to

(180) $$\begin{array}{c} \Phi \left(\alpha ,\rho \right)\leq a{\left(\rho -1\right)}^{2}+b\min\left\{\rho -1,{\left(\rho -1\right)}^{2}\right\}, \end{array}$$

where

(181) $$\begin{array}{cc} \; & a:=\frac{4\log e}{81(\alpha +1)}, \end{array}$$

(182) $$\begin{array}{cc} \; & b:=\frac{1}{4}\log\left(\alpha +1\right)+\frac{3}{8}\log e, \end{array}$$

for all ρ≥1 and $\alpha \geq e^{-\frac{3}{2}}$. The upper bounds in the right sides of (176), (177) and (180) are derived in Appendix J.

In comparison to (179), the stronger requirement that the right side of (180) is less than or equal to dloge gives the sufficient condition

(183) $$\begin{array}{c} \rho \leq \rho_{\max}\left(\alpha ,d\right):=\max\left\{\rho_{1}\left(\alpha ,d\right),\rho_{2}\left(\alpha ,d\right)\right\}, \end{array}$$

with

(184) $$\begin{array}{cc} \; & \rho_{1}\left(\alpha ,d\right):=1+\frac{\sqrt{b^{2}+4ad\log e}-b}{2a}, \end{array}$$

(185) $$\begin{array}{cc} \; & \rho_{2}\left(\alpha ,d\right):=1+\sqrt{\frac{d\log e}{a+b}}. \end{array}$$

**Figure 6:**
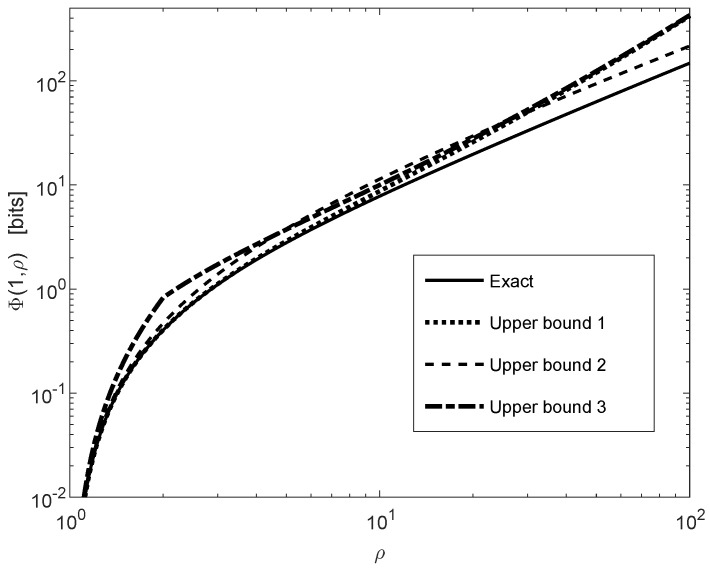
A comparison of the exact expression of Φ(α,ρ) in (175), with α=1, and its three upper bounds in the right sides of (176), (177) and (180) (called ’Upper bound 1’ (dotted line), ’Upper bound 2’ (thin dashed line), and ’Upper bound 3’ (thick dashed line), respectively).

Figure 6 compares the exact expression in (175) with its upper bounds in (176), (177) and (180). These bounds show good match with the exact value, and none of the bounds in (176) and (177) is superseded by the other; the bound in (180) is looser than (176), and it is derived for obtaining the closed-form solution in (183)–(185). The bound in (176) is tighter than the bound in (177) for small values of ρ≥1, whereas the latter bound outperforms the first one for sufficiently large values of ρ. It has been observed numerically that the tightness of the bounds is improved by increasing the value of α, and the range of parameters of ρ over which the bound in (176) outperforms the second bound in (177) is enlarged when α is increased. It is also shown in Figure 6 that the bound in (176) and its loosened version in (180) almost coincide for sufficiently small values of ρ (i.e., for ρ is close to 1), and also for sufficiently large values of ρ.

### 3.4. An Interpretation of $u_{f}\left(\cdot ,\cdot \right)$ in Theorem 7

We provide here an interpretation of $u_{f}\left(n,\rho \right)$ in (77), for ρ>1 and an integer n≥2; note that $u_{f}\left(n,1\right)\equiv 0$ since $P_{n}\left(1\right)=\left\{U_{n}\right\}$. Before doing so, recall that (82) introduces an identity which significantly simplifies the numerical calculation of $u_{f}\left(n,\rho \right)$, and (85) gives (asymptotically tight) upper and lower bounds.

The following result relies on the variational representation of *f*-divergences.

**Theorem** **10.** *Let f:(0,∞)→R be convex with f(1)=0, and let $\bar{f}:R\to R\cup \left\{\infty \right\}$ be the convex conjugate function of f (a.k.a. the Fenchel-Legendre transform of f), i.e.,*

(186) $$\begin{array}{c} \bar{f}\left(x\right):=\underset{t>0}{\sup}\left\{tx-f(t)\right\},\;x\in R. \end{array}$$

*Let ρ>1, and define $A_{n}:=\left\{1,\ldots ,n\right\}$ for an integer n≥2. Then, the following holds:*

*(a)*  *For every $P\in P_{n}\left(\rho \right)$, a random variable X∼P, and a function $g:A_{n}\to R$,*
  (187) $$\begin{array}{c} E\left[g\left(X\right)\right]\leq u_{f}\left(n,\rho \right)+\frac{1}{n}\sum_{i=1}^{n}\bar{f}\left(g(i)\right). \end{array}$$
*(b)*  *There exists $P\in P_{n}\left(\rho \right)$ such that, for every ε>0, there is a function $g_{\varepsilon }:A_{n}\to R$ which satisfies*
  (188) $$\begin{array}{c} E\left[g_{\varepsilon }\left(X\right)\right]\geq u_{f}\left(n,\rho \right)+\frac{1}{n}\sum_{i=1}^{n}\bar{f}\left(g_{\varepsilon }\left(i\right)\right)-\varepsilon , \end{array}$$
  *with X∼P.*

**Proof.**  See Appendix K.  □

**Remark** **7.** *The proof suggests a constructive way to obtain, for an arbitrary ε>0, a function $g_{\varepsilon }$ which satisfies* (188)*.*

## 4. Applications in Information Theory and Statistics

### 4.1. Bounds on the List Decoding Error Probability with *f*-Divergences

The minimum probability of error of a random variable *X* given *Y*, denoted by $\varepsilon_{X|Y}$, can be achieved by a deterministic function (*maximum-a-posteriori* decision rule) $L^{*}:Y\to X$ (see [42]):

(189) $$\begin{array}{cc} \varepsilon_{X|Y}\; & =\min_{L:Y\to X}P\left[X\neq L\left(Y\right)\right] \end{array}$$

(190) $$\begin{array}{cc} \; & =P[X\neq L^{*}\left(Y\right)] \end{array}$$

(191) $$\begin{array}{cc} \; & =1-E\left[\max_{x\in X}P_{X|Y}\left(x|Y\right)\right]. \end{array}$$

Fano’s inequality [46] gives an upper bound on the conditional entropy H(X|Y) as a function of $\varepsilon_{X|Y}$ (or, otherwise, providing a lower bound on $\varepsilon_{X|Y}$ as a function of H(X|Y)) when *X* takes a finite number of possible values.

The list decoding setting, in which the hypothesis tester is allowed to output a subset of given cardinality, and an error occurs if the true hypothesis is not in the list, has great interest in information theory. A generalization of Fano’s inequality to list decoding, in conjunction with the blowing-up lemma ([17], Lemma 1.5.4), leads to strong converse results in multi-user information theory. This approach was initiated in ([47], Section 5) (see also ([48], Section 3.6)). The main idea of the successful combination of these two tools is that, given a code, it is possible to blow-up the decoding sets in a way that the probability of decoding error can be as small as desired for sufficiently large blocklengths; since the blown-up decoding sets are no longer disjoint, the resulting setup is a list decoder with sub-exponential list size (as a function of the block length).

In statistics, Fano’s-type lower bounds on Bayes and minimax risks, expressed in terms of *f*-divergences, are derived in [49,50].

In this section, we further study the setup of list decoding, and derive bounds on the average list decoding error probability. We first consider the special case where the list size is fixed (see Section 4.1.1), and then move to the more general case of a list size which depends on the channel observation (see Section 4.1.2).

#### 4.1.1. Fixed-Size List Decoding

A generalization of Fano’s inequality for fixed-size list decoding is given in ([42], (139)), expressed as a function of the conditional Shannon entropy (strengthening ([51], Lemma 1)). A further generalization in this setup, which is expressed as a function of the Arimoto-Rényi conditional entropy with an arbitrary positive order (see Definition 9), is provided in ([42], Theorem 8).

The next result provides a generalized Fano’s inequality for fixed-size list decoding, expressed in terms of an arbitrary *f*-divergence. Some earlier results in the literature are reproduced from the next result, followed by its strengthening as an application of Theorem 1.

**Theorem** **11.** *Let $P_{XY}$ be a probability measure defined on X×Y with |X|=M. Consider a decision rule L:Y→XL, where XL stands for the set of subsets of X with cardinality L, and L<M is fixed. Denote the list decoding error probability by $P_{L}:=P\left[X\notin L(Y)\right]$. Let $U_{M}$ denote an equiprobable probability mass function on X. Then, for every convex function f:(0,∞)→R with f(1)=0,*

(192) $$\begin{array}{c} E\left[D_{f}\left(P_{X|Y}\left(\cdot |Y\right)\;∥\;U_{M}\right)\right]\geq \frac{L}{M}\;f\left(\frac{M\;(1-P_{L})}{L}\right)+\left(1-\frac{L}{M}\right)\;f\left(\frac{MP_{L}}{M-L}\right). \end{array}$$

**Proof.**  See Appendix L.  □

**Remark** **8.** *The case where L=1 (i.e., a decoder with a single output) gives ([50], (5)).*

As consequences of Theorem 11, we first reproduce some earlier results as special cases.

**Corollary** **2** ([42] (139))**.** *Under the assumptions in Theorem 11,*

(193) $$\begin{array}{c} H\left(X|Y\right)\leq \log M-d\left(P_{L}\;∥\;1-\frac{L}{M}\right) \end{array}$$

*where d(·∥·):[0,1]×[0,1]→[0,+∞] denotes the binary relative entropy, defined as the continuous extension of $D\left(\left[p,1-p\right]∥\left[q,1-q\right]\right):=p\log\frac{p}{q}+\left(1-p\right)\log\frac{1-p}{1-q}$ for p,q∈(0,1).*

**Proof.**  The choice f(t):=tlogt+(1−t)loge, for t>0, (note that $f\left(t\right)=u_{1}\left(t\right)\log e$ with $u_{1}\left(\cdot \right)$ defined in (139)) gives

(194) $$\begin{array}{cc} E\left[D_{f}\left(P_{X|Y}\left(\cdot |Y\right)\;∥\;U_{M}\right)\right]\; & =\int_{Y}dP_{Y}\left(y\right)\;D\left(P_{X|Y}\left(\cdot |y\right)\;∥U_{M}\right) \end{array}$$

(195) $$\begin{array}{cc} \; & =\int_{Y}dP_{Y}\left(y\right)\;\left[\log M-H(X|Y=y)\right] \end{array}$$

(196) $$\begin{array}{cc} \; & =\log M-H(X|Y), \end{array}$$

and

(197) $$\begin{array}{c} \frac{L}{M}\;f\left(\frac{M\;(1-P_{L})}{L}\right)+\left(1-\frac{L}{M}\right)\;f\left(\frac{MP_{L}}{M-L}\right)=d\left(P_{L}\;∥\;1-\frac{L}{M}\right). \end{array}$$

Substituting (194)–(197) into (192) gives (193).  □

Theorem 11 enables to reproduce a result in [42] which generalizes Corollary 2. It relies on Rényi information measures, and we first provide definitions for a self-contained presentation.

**Definition** **8** ([40])**.** *Let $P_{X}$ be a probability mass function defined on a discrete set X. The Rényi entropy of order α∈(0,1)∪(1,∞) of X, denoted by $H_{\alpha }\left(X\right)$ or $H_{\alpha }\left(P_{X}\right)$, is defined as*

(198) $$\begin{array}{cc} H_{\alpha }\left(X\right) & :=\frac{1}{1-\alpha }\;\log\sum_{x\in X}P_{X}^{\alpha }\left(x\right) \end{array}$$

(199) $$\begin{array}{cc} \; & \;=\frac{\alpha }{1-\alpha }\;\log{∥P_{X}∥}_{\alpha }. \end{array}$$

*The Rényi entropy is continuously extended at orders* 0*,* 1*, and* ∞*; at order 1, it coincides with the Shannon entropy H(X).*

**Definition** **9** ([52])**.** *Let $P_{XY}$ be defined on X×Y, where X is a discrete random variable. The Arimoto-Rényi conditional entropy of order α∈[0,∞] of X given Y is defined as follows:*

- *If α∈(0,1)∪(1,∞), then*
  (200) $$\begin{array}{cc} H_{\alpha }\left(X|Y\right) & =\frac{\alpha }{1-\alpha }\;\log\;E\left[{\left(\;\sum_{x\in X}P_{X|Y}^{\alpha }\left(x|Y\right)\right)}^{\frac{1}{\alpha }}\right] \end{array}$$
  (201) $$\begin{array}{cc} \; & =\frac{\alpha }{1-\alpha }\;\log E\left[∥P_{X|Y}{\left(\cdot |Y\right)∥}_{\alpha }\right] \end{array}$$
  (202) $$\begin{array}{cc} \; & =\frac{\alpha }{1-\alpha }\;\log\;\int_{Y}dP_{Y}\left(y\right)\;\exp\left(\frac{1-\alpha }{\alpha }\;H_{\alpha }\left(X|Y=y\right)\right). \end{array}$$
- *The Arimoto-Rényi conditional entropy is continuously extended at orders* 0*,* 1*, and* ∞; *at order 1, it coincides with the conditional Shannon entropy H(X|Y).*

**Definition** **10** ([42])**.** *For all α∈(0,1)∪(1,∞), the binary Rényi divergence of order α, denoted by $d_{\alpha }\left(p∥q\right)$ for $\left(p,q\right)\in {\left[0,1\right]}^{2}$, is defined as $D_{\alpha }\left(\left[p,1-p\right]\;∥\;\left[q,1-q\right]\right)$. It is the continuous extension to ${\left[0,1\right]}^{2}$ of*

(203) $$\begin{array}{c} d_{\alpha }\left(p∥q\right)=\frac{1}{\alpha -1}\;\log\left(p^{\alpha }q^{1-\alpha }+{\left(1-p\right)}^{\alpha }{\left(1-q\right)}^{1-\alpha }\right). \end{array}$$

*For α=1,*

(204) $$\begin{array}{c} d_{1}\left(p∥q\right):=\lim_{\alpha \to 1}d_{\alpha }\left(p∥q\right)=d\left(p∥q\right). \end{array}$$

The following result, generalizing Corollary 2, is shown to be a consequence of Theorem 11. It has been originally derived in ([42], Theorem 8) in a different way. The alternative derivation of this inequality relies on Theorem 11, applied to the family of Alpha-divergences (see (138)) as a subclass of the *f*-divergences.

**Corollary** **3** ([42] Theorem 8)**.** *Under the assumptions in Theorem 11, for α∈(0,1)∪(1,∞),*

(205) $$\begin{array}{cc} H_{\alpha }\left(X|Y\right) & \leq \log M-d_{\alpha }\left(P_{L}\;∥\;1-\frac{L}{M}\right) \end{array}$$

(206) $$\begin{array}{cc} \; & =\frac{1}{1-\alpha }\;\log\left(L^{1-\alpha }\;{\left(1-P_{L}\right)}^{\alpha }+{\left(M-L\right)}^{1-\alpha }\;P_{L}^{\alpha }\right), \end{array}$$

*with equality in* (205) *if and only if*

(207) $$P_{X|Y}\left(x|y\right)=\{\begin{array}{cc} \frac{P_{L}}{M-L}, & x\notin L(y),\\ \frac{1-P_{L}}{L}, & x\in L(y). \end{array}$$

**Proof.**  See Appendix M.  □

Another application of Theorem 11 with the selection $f\left(t\right):={\left|t-1\right|}^{s}$, for t∈[0,∞) and a parameter s≥1, gives the following result.

**Corollary** **4.** *Under the assumptions in Theorem 11, for all s≥1,*

(208) $$\begin{array}{c} P_{L}\geq 1-\frac{L}{M}-{\left(L^{1-s}+{\left(M-L\right)}^{1-s}\right)}^{-\frac{1}{s}}{\left(E\left[\;\sum_{x\in X}\;{\left|P_{X|Y}\left(x|Y\right)-\frac{1}{M}\right|}^{s}\right]\right)}^{\frac{1}{s}}, \end{array}$$

*where* (208) *holds with equality if X and Y are independent with X being equiprobable. For s=1 and s=2,* (208) *respectively gives that*

(209) $$\begin{array}{cc} \; & P_{L}\geq 1-\frac{L}{M}-\frac{1}{2}\;E\left[\;\sum_{x\in X}\;\left|P_{X|Y}\left(x|Y\right)-\frac{1}{M}\right|\right], \end{array}$$

(210) $$\begin{array}{cc} \; & P_{L}\geq 1-\frac{L}{M}-\sqrt{\frac{L}{M}\left(1-\frac{L}{M}\right)\left(M\;E[P_{X|Y}\left(X|Y\right)]-1\right)}. \end{array}$$

The following refinement of the generalized Fano’s inequality in Theorem 11 relies on the version of the strong data-processing inequality in Theorem 1.

**Theorem** **12.** *Under the assumptions in Theorem 11, let the convex function f:(0,∞)→R be twice differentiable, and assume that there exists a constant $m_{f}>0$ such that*

(211) $$\begin{array}{c} f^{\prime \prime }\left(t\right)\geq m_{f},\;\forall \;t\in I\left(\xi_{1}^{*},\xi_{2}^{*}\right), \end{array}$$

*where*

(212) $$\begin{array}{cc} \; & \xi_{1}^{*}:=M\underset{(x,y)\in X\times Y}{\inf}P_{X|Y}\left(x|y\right), \end{array}$$

(213) $$\begin{array}{cc} \; & \xi_{2}^{*}:=M\underset{(x,y)\in X\times Y}{\sup}P_{X|Y}\left(x|y\right), \end{array}$$

*and the interval I(·,·) is defined in* (23)*. Let $u^{+}:=\max\left\{u,0\right\}$ for u∈R. Then,*

*(a)*  (214) $$\begin{array}{cc} E\left[D_{f}\left(P_{X|Y}\left(\cdot |Y\right)\;∥\;U_{M}\right)\right]\; & \geq \frac{L}{M}\;f\left(\frac{M\;(1-P_{L})}{L}\right)+\left(1-\frac{L}{M}\right)\;f\left(\frac{MP_{L}}{M-L}\right)\\ \; & \;+\frac{1}{2}m_{f}\;M{\left(E\left[P_{X|Y}\left(X|Y\right)\right]-\frac{1-P_{L}}{L}-\frac{P_{L}}{M-L}\right)}^{+}. \end{array}$$
*(b)*  *If the list decoder selects the L most probable elements from X, given the value of Y∈Y, then* (214) *is strengthened to*
  (215) $$\begin{array}{cc} E\left[D_{f}\left(P_{X|Y}\left(\cdot |Y\right)\;∥\;U_{M}\right)\right]\; & \geq \frac{L}{M}\;f\left(\frac{M\;(1-P_{L})}{L}\right)+\left(1-\frac{L}{M}\right)\;f\left(\frac{MP_{L}}{M-L}\right)\\ \; & \;+\frac{1}{2}m_{f}\;M\left(E\left[P_{X|Y}\left(X|Y\right)\right]-\frac{1-P_{L}}{L}\right), \end{array}$$
  *where the last term in the right side of (215) is necessarily non-negative.*

**Proof.**  See Appendix N.  □

An application of Theorem 12 gives the following tightened version of Corollary 2.

**Corollary** **5.** *Under the assumptions in Theorem 11, the following holds:*

*(a)*  *Inequality* (193) *is strengthened to*
  (216) $$\begin{array}{cc} H(X|Y)\leq & \log M-d\left(P_{L}\;∥\;1-\frac{L}{M}\right)-\frac{\log e}{2}\;\frac{{\left(E\left[P_{X|Y}\left(X|Y\right)\right]-\frac{1-P_{L}}{L}-\frac{P_{L}}{M-L}\right)}^{+}}{\underset{(x,y)\in X\times Y}{\sup}P_{X|Y}\left(x|y\right)}. \end{array}$$
*(b)*  *If the list decoder selects the L most probable elements from X, given the value of Y∈Y, then* (216) *is strengthened to*
  (217) $$\begin{array}{c} H\left(X|Y\right)\leq \log M-d\left(P_{L}\;∥\;1-\frac{L}{M}\right)-\frac{\log e}{2}\cdot \frac{{\left(E\left[P_{X|Y}\left(X|Y\right)\right]-\frac{1-P_{L}}{L}\right)}^{+}}{\underset{(x,y)\in X\times Y}{\sup}P_{X|Y}\left(x|y\right)}. \end{array}$$

**Proof.**  The choice f(t):=tlogt+(1−t)loge, for t>0, gives (see (23) and (211)–(213))

(218) $$\begin{array}{cc} m_{f}\;M & =M\underset{t\in I(\xi_{1}^{*},\xi_{2}^{*})}{\inf}f^{\prime \prime }t)\\ \; & =\frac{M\log e}{\xi_{2}^{*}}\\ \; & =\frac{\log e}{\underset{(x,y)\in X\times Y}{\sup}P_{X|Y}\left(x|y\right)}. \end{array}$$

Substituting (194)–(197) and (218) into (214) and (215) give, respectively, (216) and (217).  □

**Remark** **9.** *Similarly to the bounds on $P_{L}$ in* (193) *and* (205)*, which tensorize when $P_{X|Y}$ is replaced by a product probability measure $P_{X^{n}|Y^{n}}\left(\underline{x}|\underline{y}\right)=\overset{n}{\prod_{i=1}}P_{X_{i}|Y_{i}}\left(x_{i}|y_{i}\right)$, this is also the case with the new bounds in* (216) *and* (217)*.*

**Remark** **10.** *The ceil operation in the right side of* (217) *is redundant with $P_{L}$ denoting the list decoding error probability (see* (A335)*–*(A341)*). However, for obtaining a lower bound on $P_{L}$ with* (217)*, the ceil operation assures that the bound is at least as good as the lower bound which relies on the generalized Fano’s inequality in* (193)*.*

**Example** **1.** *Let X and Y be discrete random variables taking values in X={0,1,…,8} and Y={0,1}, respectively, and let $P_{XY}$ be the joint probability mass function, given by*

(219) $$\begin{array}{c} {\left[P_{XY}\left(x,y\right)\right]}_{(x,y)\in X\times Y}=\frac{1}{512}{\left(\begin{array}{ccccccccc} 128 & 64 & 32 & 16 & 8 & 4 & 2 & 1 & 1\\ 2 & 2 & 2 & 2 & 8 & 16 & 32 & 64 & 128 \end{array}\right)}^{T}. \end{array}$$

*Let the list decoder select the L most probable elements from X, given the value of Y∈Y. Table 1 compares the list decoding error probability $P_{L}$ with the lower bound which relies on the generalized Fano’s inequality in* (193)*, its tightened version in* (217)*, and the closed-form lower bound in* (210) *for fixed list sizes of L=1,…,4. For L=3 and L=4,* (217) *improves the lower bound in* (193) *(see Table 1). If L=4, then the generalized Fano’s lower bound in* (193) *and also* (210) *are useless, whereas* (217) *gives a non-trivial lower bound. It is shown here that none of the new lower bounds in* (210) *and* (217) *is superseded by the other.*

#### 4.1.2. Variable-Size List Decoding

In the more general setting of list decoding where the size of the list may depend on the channel observation, Fano’s inequality has been generalized as follows.

**Proposition** **5** (([48], Appendix 3.E) and [53])**.** *Let $P_{XY}$ be a probability measure defined on X×Y with |X|=M. Consider a decision rule $L:Y\to 2^{X}$, and let the (average) list decoding error probability be given by $P_{L}:=P\left[X\notin L(Y)\right]$ with |L(y)|≥1 for all y∈Y. Then,*

(220) $$\begin{array}{c} H\left(X|Y\right)\leq h\left(P_{L}\right)+E[\log|L\left(Y\right)\left|\right]+P_{L}\log M, \end{array}$$

*where h:[0,1]→[0,log2] denotes the binary entropy function. If |L(Y)|≤N almost surely, then also*

(221) $$\begin{array}{c} H\left(X|Y\right)\leq h\left(P_{L}\right)+\left(1-P_{L}\right)\log N+P_{L}\log M. \end{array}$$

By relying on the data-processing inequality for *f*-divergences, we derive in the following an alternative explicit lower bound on the average list decoding error probability $P_{L}$. The derivation relies on the $E_{\gamma }$ divergence (see, e.g., [54]), which forms a subclass of the *f*-divergences.

**Theorem** **13.** *Under the assumptions in* (220)*, for every γ≥1,*

(222) $$\begin{array}{c} P_{L}\geq \frac{1+\gamma }{2}-\frac{\gamma E[|L(Y)|]}{M}-\frac{1}{2}\;E\left[\;\sum_{x\in X}\;\left|P_{X|Y}\left(x|Y\right)-\frac{\gamma }{M}\right|\right]. \end{array}$$

*Let γ≥1, and let $\left|L\left(y\right)\right|\leq \frac{M}{\gamma }$ for all y∈Y. Then,* (222) *holds with equality if, for every y∈Y, the list decoder selects the |L(y)| most probable elements in X given Y=y; if $x_{\ell }\left(y\right)$ denotes the ℓ-th most probable element in X given Y=y, where ties in probabilities are resolved arbitrarily, then* (222) *holds with equality if*

(223) $$P_{X|Y}\left(x_{\ell }\left(y\right)\;|y\right)=\{\begin{array}{cc} \alpha (y), & \forall \;\ell \in \left\{1,\ldots ,|L(y)|\right\},\\ \frac{1-\alpha (y)\;|L(y)|}{M-|L(y)|}, & \forall \;\ell \in \{|L(y)|+1,\ldots ,M\}, \end{array}$$

*with α:Y→[0,1] being an arbitrary function which satisfies*

(224) $$\begin{array}{c} \frac{\gamma }{M}\leq \alpha \left(y\right)\leq \frac{1}{\left|L(y)\right|},\;\forall \;y\in Y. \end{array}$$

**Proof.**  See Appendix O.  □

**Remark** **11.** *By setting γ=1 and |L(Y)|=L (i.e., a decoding list of fixed size L),* (222) *is specialized to* (209)*.*

**Example** **2.** *Let X and Y be discrete random variables taking their values in X={0,1,2,3,4} and Y={0,1}, respectively, and let $P_{XY}$ be their joint probability mass function, which is given by*

(225) $$\left\{ \begin{array}{cc} P_{XY}\left(0,0\right)=P_{XY}\left(1,0\right)=P_{XY}\left(2,0\right)=\frac{1}{8}, & P_{XY}\left(3,0\right)=P_{XY}\left(4,0\right)=\frac{1}{16},\\ P_{XY}\left(0,1\right)=P_{XY}\left(1,1\right)=P_{XY}\left(2,1\right)=\frac{1}{24}, & P_{XY}\left(3,1\right)=P_{XY}\left(4,1\right)=\frac{3}{16}. \end{array}\right.$$

*Let L(0):={0,1,2} and L(1):={3,4} be the lists in X, given the value of Y∈Y. We get $P_{Y}\left(0\right)=P_{Y}\left(1\right)=\frac{1}{2}$, so the conditional probability mass function of X given Y satisfies $P_{X|Y}\left(x|y\right)=2P_{XY}\left(x,y\right)$ for all (x,y)∈X×Y. It can be verified that, if $\gamma =\frac{5}{4}$, then $\max\left\{|L\left(0\right)|,|L\left(1\right)|\right\}=3\leq \frac{M}{\gamma }$, and also* (223) *and* (224) *are satisfied (here, M:=|X|=5, $\alpha \left(0\right)=\frac{1}{4}=\frac{\gamma }{M}$ and $\alpha \left(1\right)=\frac{3}{8}\in \left[\frac{1}{4},\frac{1}{2}\right]$). By Theorem 13, it follows that* (222) *holds in this case with equality, and the list decoding error probability is equal to $P_{L}=1-E\left[\alpha (Y)\;|L(Y)|\right]=\frac{1}{4}$ (i.e., it coincides with the lower bound in the right side of* (222) *with $\gamma =\frac{5}{4}$). On the other hand, the generalized Fano’s inequality in* (220) *gives that $P_{L}\geq 0.1206$ (the left side of* (220) *is $H\left(X|Y\right)=\frac{5}{2}\;\log2-\frac{1}{4}\;\log3=2.1038$ bits); moreover, by letting $N:=\max_{y\in Y}\;\left|L\left(y\right)\right|=3$,* (221) *gives the looser lower bound $P_{L}\geq 0.0939$. This exemplifies a case where the lower bound in Theorem 13 is tight, whereas the generalized Fano’s inequalities in* (220) *and* (221) *are looser.*

### 4.2. A Measure for the Approximation of Equiprobable Distributions by Tunstall Trees

The best possible approximation of equiprobable distributions, which one can get by using tree codes has been considered in [38]. The optimal solution is obtained by using Tunstall codes, which are variable-to-fixed lossless compression codes (see ([55], Section 11.2.3), [56]). The main idea behind Tunstall codes is parsing the source sequence into variable-length segments of roughly the same probability, and then coding all these segments with codewords of fixed length. This task is done by assigning the leaves of a Tunstall tree, which correspond to segments of source symbols with a variable length (according to the depth of the leaves in the tree), to codewords of fixed length. The following result links Tunstall trees with majorization theory.

**Proposition** **6** ([38] Theorem 1)**.** *Let $P_{\ell }$ be the probability measure generated on the leaves by a Tunstall tree T, and let $Q_{\ell }$ be the probability measure generated by an arbitrary tree S with the same number of leaves as of T. Then, $P_{\ell }≺Q_{\ell }$.*

From Proposition 6, and the Schur-convexity of an *f*-divergence $D_{f}\left(\cdot ∥U_{n}\right)$ (see ([38], Lemma 1)), it follows that (see ([38], Corollary 1))

(226) $$\begin{array}{c} D_{f}\left(P_{\ell }∥U_{n}\right)\leq D_{f}\left(Q_{\ell }∥U_{n}\right), \end{array}$$

where *n* designates the joint number of leaves of the trees T and S.

Before we proceed, it is worth noting that the strong data-processing inequality in Theorem 6 implies that if *f* is also twice differentiable, then (226) can be strengthened to

(227) $$\begin{array}{c} D_{f}\left(P_{\ell }∥U_{n}\right)+nc_{f}\left(nq_{\min},nq_{\max}\right)\left(∥Q_{\ell }{∥}_{2}^{2}-{∥P_{\ell }∥}_{2}^{2}\right)\leq D_{f}\left(Q_{\ell }∥U_{n}\right), \end{array}$$

where $q_{\max}$ and $q_{\min}$ denote, respectively, the maximal and minimal positive masses of $Q_{\ell }$ on the *n* leaves of a tree S, and $c_{f}\left(\cdot ,\cdot \right)$ is given in (26).

We next consider a measure which quantifies the quality of the approximation of the probability mass function $P_{\ell }$, induced by the leaves of a Tunstall tree, by an equiprobable distribution $U_{n}$ over a set whose cardinality (*n*) is equal to the number of leaves in the tree. To this end, consider the setup of Bayesian binary hypothesis testing where a random variable *X* has one of the two probability distributions

(228) $$\left\{ \begin{array}{cc} H_{0}: & X∼P_{\ell },\\ H_{1}: & X∼U_{n}, \end{array}\right.$$

with a-priori probabilities $P[H_{0}]=\omega$, and $P[H_{1}]=1-\omega$ for an arbitrary ω∈(0,1). The measure being considered here is equal to the difference between the minimum a-priori and minimum a-posteriori error probabilities of the Bayesian binary hypothesis testing model in (228), which is close to zero if the two distributions are sufficiently close.

The difference between the minimum a-priori and minimum a-posteriori error probabilities of a general Bayesian binary hypothesis testing model with the two arbitrary alternative hypotheses $H_{0}:\;X∼P$ and $H_{1}:\;X∼Q$ with a-priori probabilities ω and 1−ω, respectively, is defined to be the order-ω DeGroot statistical information $I_{\omega }\left(P,Q\right)$ [57] (see also ([16], Definition 3)). It can be expressed as an *f*-divergence:

(229) $$\begin{array}{c} I_{\omega }\left(P,Q\right)=D_{\phi_{\omega }}\left(P∥Q\right), \end{array}$$

where $\phi_{\omega }:\left[0,\infty \right)\to R$ is the convex function with $\phi_{\omega }\left(1\right)=0$, given by (see ([16], (73)))

(230) $$\begin{array}{c} \phi_{\omega }\left(t\right):=\min\left\{\omega ,1-\omega \right\}-\min\left\{\omega ,1-\omega t\right\},\;t\geq 0. \end{array}$$

The measure considered here for quantifying the closeness of $P_{\ell }$ to the equiprobable distribution $U_{n}$ is therefore given by

(231) $$\begin{array}{c} d_{\omega ,n}\left(P_{\ell }\right):=D_{\phi_{\omega }}\left(P_{\ell }∥U_{n}\right),\;\forall \;\omega \in \left(0,1\right), \end{array}$$

which is bounded in the interval $\left[0,\min\{\omega ,1-\omega \}\right]$.

The next result partially relies on Theorem 7.

**Theorem** **14.** *The measure in* (231) *satisfies the following properties:*

*(a)*  It is the minimum of $D_{\phi_{\omega }}\left(P∥U_{n}\right)$ with respect to all probability measures $P\in P_{n}$ that are induced by an arbitrary tree with n leaves.
*(b)*  (232) $$\begin{array}{cc} d_{\omega ,n}\left(P_{\ell }\right) & \leq \max_{\beta \in \Gamma_{n}\left(\rho \right)}D_{\phi_{\omega }}\left(Q_{\beta }∥U_{n}\right), \end{array}$$
  *with the function $\phi_{\omega }\left(\cdot \right)$ in* (230)*, the interval $\Gamma_{n}\left(\rho \right)$ in* (79)*, the probability mass function $Q_{\beta }$ in* (80)*, and $\rho :=\frac{1}{p_{\min}}$ is the reciprocal of the minimal probability of the source symbols.*
*(c)*  *The following bound holds for every n∈N, which is the asymptotic limit of the right side of* (232) *as we let n→∞:*
  (233) $$\begin{array}{c} d_{\omega ,n}\left(P_{\ell }\right)\leq \max_{x\in [0,1]}\left\{x\;\phi_{\omega }\left(\frac{\rho }{1+(\rho -1)x}\right)+\left(1-x\right)\;\phi_{\omega }\left(\frac{1}{1+(\rho -1)x}\right)\right\}. \end{array}$$
*(d)*  *If f:(0,∞)→R is convex and twice differentiable, continuous at zero and f(1)=0, then*
  (234) $$\begin{array}{c} D_{f}\left(P_{\ell }∥U_{n}\right)=\int_{0}^{1}\frac{d_{\omega ,n}\left(P_{\ell }\right)}{\omega^{3}}\;f^{\prime \prime }\left(\frac{1-\omega }{\omega }\right)\;d\omega . \end{array}$$

**Proof.**  See Appendix P.1.  □

**Remark** **12.** *The integral representation in* (234) *provides another justification for quantifying the closeness of $P_{\ell }$ to an equiprobable distribution by the measure in* (231)*.*

**Figure 7:**
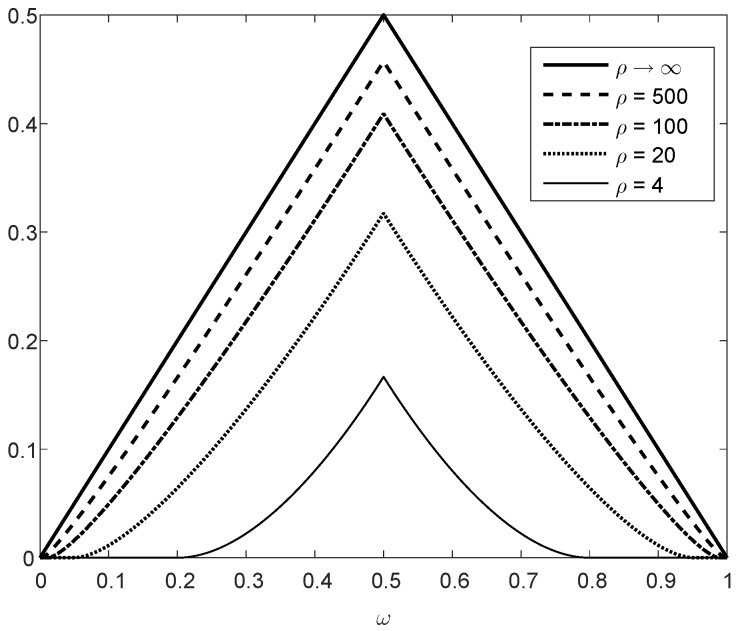
Curves of the upper bound on the measure $d_{\omega ,n}\left(P_{\ell }\right)$ in (233), valid for all n∈N, as a function of ω∈[0,1] for different values of $\rho :=\frac{1}{p_{\min}}$.

Figure 7 refers to the upper bound on the closeness-to-equiprobable measure $d_{\omega ,n}\left(P_{\ell }\right)$ in (233) for Tunstall trees with *n* leaves. The bound holds for all n∈N, and it is shown as a function of ω∈[0,1] for several values of ρ∈[1,∞]. In the limit where ρ→∞, the upper bound is equal to min{ω,1−ω} since the minimum a-posteriori error probability of the Bayesian binary hypothesis testing model in (228) tends to zero. On the other hand, if ρ=1, then the right side of (233) is identically equal to zero (since $\phi_{\omega }\left(1\right)=0$).

Theorem 14 gives an upper bound on the measure in (231), for the closeness of the probability mass function generated on the leaves by a Tunstall tree to the equiprobable distribution, where this bound is expressed as a function of the minimal probability mass of the source. The following result, which relies on ([33], Theorem 4) and our earlier analysis related to Theorem 7, provides a sufficient condition on the minimal probability mass for asserting the closeness of the compression rate to the Shannon entropy of a stationary and memoryless discrete source.

**Theorem** **15.** *Let P be a probability mass function of a stationary and memoryless discrete source, and let the emitted source symbols be from an alphabet of size D≥2. Let C be a Tunstall code which is used for source compression; let m and X denote, respectively, the fixed length and the alphabet of the codewords of C (where |X|≥2), referring to a Tunstall tree of n leaves with $n\leq {\left|X\right|}^{m}<n+\left(D-1\right)$. Let $p_{\min}$ be the minimal probability mass of the source symbols, and let*

(235) $$d=d\left(m,\varepsilon \right):=\{\begin{array}{cc} \frac{m\varepsilon \;\log_{e}\left|X\right|}{1+\varepsilon }+\log_{e}\left(1-\frac{D-1}{{\left|X\right|}^{m}}\right), & if\;D>2,\\ \frac{m\varepsilon \;\log_{e}\left|X\right|}{1+\varepsilon }, & if\;D=2, \end{array}$$

*with an arbitrary ε>0 such that d>0. If*

(236) $$\begin{array}{c} p_{\min}\geq \frac{W_{0}\left(-e^{-d-1}\right)}{W_{-1}\left(-e^{-d-1}\right)}, \end{array}$$

*where $W_{0}$ and $W_{-1}$ denote, respectively, the principal and secondary real branches of the Lambert W function [37], then the compression rate of the Tunstall code is larger than the Shannon entropy of the source by a factor which is at most 1+ε.*

**Proof.**  See Appendix P.2.  □

**Remark** **13.** *The condition in* (236) *can be replaced by the stronger requirement that*

(237) $$\begin{array}{c} p_{\min}\geq \frac{1}{1+\sqrt{8d}}. \end{array}$$

**Figure 8:**
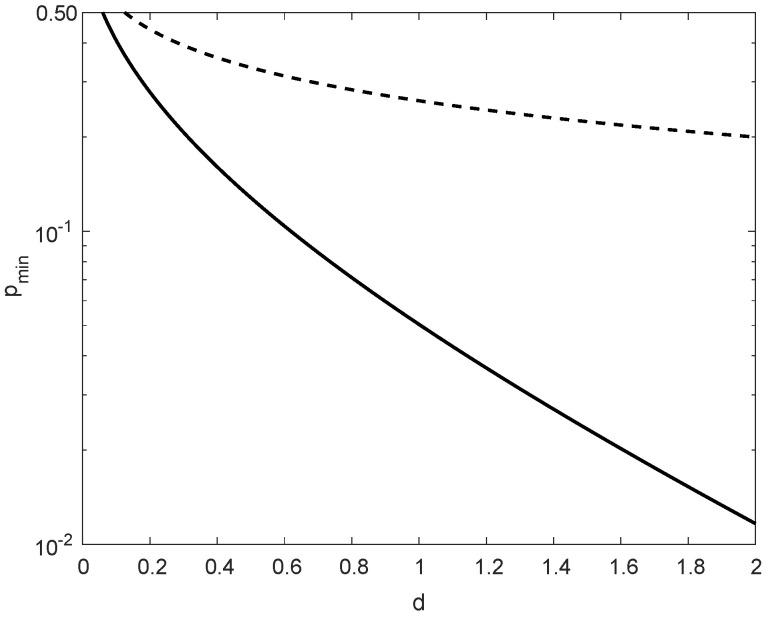
Curves for the smallest values of $p_{\min}$, in the setup of Theorem 15, according to the condition in (236) (solid line) and the more restrictive condition in (237) (dashed line) for binary Tunstall codes which are used to compress memoryless and stationary binary sources.

*Unless d is a small fraction of unity, there is a significant difference between the condition in* (236) *and the more restrictive condition in* (237) *(see Figure 8).*

**Example** **3.** *Consider a memoryless and stationary binary source, and a binary Tunstall code with codewords of length m=10 referring to a Tunstall tree with $n=2^{m}=1024$ leaves. Letting ε=0.1 in Theorem 15, it follows that if the minimal probability mass of the source satisfies $p_{\min}\geq 0.0978$ (see* (235)*, and Figure 8 with $d=\frac{m\varepsilon \log_{e}2}{1+\varepsilon }=0.6301$), then the compression rate of the Tunstall code is at most 10% larger than the Shannon entropy of the source.*

## Figures and Tables

**Table 1:** The lower bounds on $P_{L}$ in (193), (210) and (217), and its exact value for fixed list size *L* (see Example 1).

*L*	Exact $P_{L}$	(193)	(217)	(210)
1	0.500	0.353	0.353	0.444
2	0.250	0.178	0.178	0.190
3	0.125	0.065	0.072	$5.34\times 10^{-5}$
4	0.063	0	0.016	0

## Appendix A. Proof of Theorem 1

We start by proving Item (a). By our assumptions on $Q_{X}$ and $W_{Y|X}$,

(A1) $$\begin{array}{cc} \; & P_{X}\left(x\right),Q_{X}\left(x\right)>0,\;\;\forall \;x\in X, \end{array}$$

(A2) $$\begin{array}{cc} \; & \sum_{x\in X}W_{Y|X}\left(y|x\right)>0,\;\forall \;y\in Y, \end{array}$$

(A3) $$\begin{array}{cc} \; & \sum_{y\in Y}W_{Y|X}\left(y|x\right)=1,\;\;\forall \;x\in X, \end{array}$$

(A4) $$\begin{array}{cc} \; & W_{Y|X}\left(y|x\right)\geq 0,\;\;\forall \;\left(x,y\right)\in X\times Y. \end{array}$$

From (20), (21), (A1), (A2) and (A4), it follows that

(A5) $$\begin{array}{cc} \; & P_{Y}\left(y\right)=\sum_{x\in X}P_{X}\left(x\right)W_{Y|X}\left(y|x\right)>0,\;\;\forall \;y\in Y, \end{array}$$

(A6) $$\begin{array}{cc} \; & Q_{Y}\left(y\right)=\sum_{x\in X}Q_{X}\left(x\right)W_{Y|X}\left(y|x\right)>0,\;\forall \;y\in Y, \end{array}$$

which imply that, for all y∈Y,

(A7) $$\begin{array}{c} \underset{x\in X}{\inf}\frac{P_{X}\left(x\right)}{Q_{X}\left(x\right)}\leq \frac{P_{Y}\left(y\right)}{Q_{Y}\left(y\right)}\leq \underset{x\in X}{\sup}\frac{P_{X}\left(x\right)}{Q_{X}\left(x\right)}. \end{array}$$

Since by assumption $P_{X}$ and $Q_{X}$ are supported on X, and $P_{Y}$ and $Q_{Y}$ are supported on Y (see (A5) and (A6)), it follows that the left side inequality in (A7) is strict if the infimum in the left side is equal to 0, and the right side inequality in (A7) is strict if the supremum in the right side is equal to ∞. Hence, due to (18), (19) and (23),

(A8) $$\begin{array}{c} \frac{P_{X}\left(x\right)}{Q_{X}\left(x\right)},\frac{P_{Y}\left(y\right)}{Q_{Y}\left(y\right)}\in I\left(\xi_{1},\xi_{2}\right),\;\forall \;\left(x,y\right)\in X\times Y. \end{array}$$

Since by assumption f:(0,∞)→R is convex, it follows that its right derivative $f_{+}^{\prime }\left(\cdot \right)$ exists, and it is monotonically non-decreasing and finite on (0,∞) (see, e.g., ([58], Theorem 1.2) or ([59], Theorem 24.1)). A straightforward generalization of ([60], Theorem 1.1) (see ([60], Remark 1)) gives

(A9) $$\begin{array}{cc} \; & D_{f}\left(P_{X}∥Q_{X}\right)-D_{f}\left(P_{Y}∥Q_{Y}\right)=\sum_{(x,y)\in X\times Y}\left\{Q_{X}\left(x\right)\;W_{Y|X}\left(y|x\right)\;\Delta \left(\frac{P_{X}\left(x\right)}{Q_{X}\left(x\right)},\frac{P_{Y}\left(y\right)}{Q_{Y}\left(y\right)}\right)\right\} \end{array}$$

where

(A10) $$\begin{array}{c} \Delta \left(u,v\right):=f\left(u\right)-f\left(v\right)-f_{+}^{\prime }\left(v\right)\left(u-v\right),\;u,v>0. \end{array}$$

In comparison to ([60], Theorem 1.1), the requirement that *f* is differentiable on (0,∞) is relaxed here, and the derivative of *f* is replaced by its right-side derivative. Note that if *f* is differentiable, then $\Delta \left(\frac{P_{X}\left(x\right)}{Q_{X}\left(x\right)},\frac{P_{Y}\left(y\right)}{Q_{Y}\left(y\right)}\right)$ with Δ(·,·) as defined in (A10) is Bregman’s divergence [61]. The following equality, expressed in terms of Lebesgue-Stieltjes integrals, holds by ([16], Theorem 1):

$$\begin{array}{cc} \; & \Delta \left(\frac{P_{X}\left(x\right)}{Q_{X}\left(x\right)},\frac{P_{Y}\left(y\right)}{Q_{Y}\left(y\right)}\right) \end{array}$$

(A11) $$\begin{array}{c} =\{\begin{array}{cc} \int 1\left\{s\in \left(\frac{P_{Y}\left(y\right)}{Q_{Y}\left(y\right)},\frac{P_{X}\left(x\right)}{Q_{X}\left(x\right)}\right]\right\}\;\left(\frac{P_{X}\left(x\right)}{Q_{X}\left(x\right)}-s\right)\;df_{+}^{\prime }\left(s\right), & \mathrm{if}\;\frac{P_{X}\left(x\right)}{Q_{X}\left(x\right)}\geq \frac{P_{Y}\left(y\right)}{Q_{Y}\left(y\right)},\\ \int 1\left\{s\in \left(\frac{P_{X}\left(x\right)}{Q_{X}\left(x\right)},\frac{P_{Y}\left(y\right)}{Q_{Y}\left(y\right)}\right]\right\}\;\left(s-\frac{P_{X}\left(x\right)}{Q_{X}\left(x\right)}\right)\;df_{+}^{\prime }\left(s\right), & \mathrm{if}\;\frac{P_{X}\left(x\right)}{Q_{X}\left(x\right)}<\frac{P_{Y}\left(y\right)}{Q_{Y}\left(y\right)}. \end{array} \end{array}$$

From (18), (19), (22), (A8) and (A11), if $\frac{P_{X}\left(x\right)}{Q_{X}\left(x\right)}\geq \frac{P_{Y}\left(y\right)}{Q_{Y}\left(y\right)}$, then

(A12) $$\begin{array}{cc} \Delta \left(\frac{P_{X}\left(x\right)}{Q_{X}\left(x\right)},\frac{P_{Y}\left(y\right)}{Q_{Y}\left(y\right)}\right) & \geq 2c_{f}\left(\xi_{1},\xi_{2}\right)\int_{\frac{P_{Y}\left(y\right)}{Q_{Y}\left(y\right)}}^{\frac{P_{X}\left(x\right)}{Q_{X}\left(x\right)}}\left(\frac{P_{X}\left(x\right)}{Q_{X}\left(x\right)}-s\right)\;ds\\ & =c_{f}\left(\xi_{1},\xi_{2}\right){\left(\frac{P_{X}\left(x\right)}{Q_{X}\left(x\right)}-\frac{P_{Y}\left(y\right)}{Q_{Y}\left(y\right)}\right)}^{2}, \end{array}$$

and similarly, if $\frac{P_{X}\left(x\right)}{Q_{X}\left(x\right)}<\frac{P_{Y}\left(y\right)}{Q_{Y}\left(y\right)}$, then

(A13) $$\begin{array}{cc} \Delta \left(\frac{P_{X}\left(x\right)}{Q_{X}\left(x\right)},\frac{P_{Y}\left(y\right)}{Q_{Y}\left(y\right)}\right) & \geq 2c_{f}\left(\xi_{1},\xi_{2}\right)\int_{\frac{P_{X}\left(x\right)}{Q_{X}\left(x\right)}}^{\frac{P_{Y}\left(y\right)}{Q_{Y}\left(y\right)}}\left(s-\frac{P_{X}\left(x\right)}{Q_{X}\left(x\right)}\right)\;ds\\ & =c_{f}\left(\xi_{1},\xi_{2}\right){\left(\frac{P_{X}\left(x\right)}{Q_{X}\left(x\right)}-\frac{P_{Y}\left(y\right)}{Q_{Y}\left(y\right)}\right)}^{2}. \end{array}$$

By combining (A9), (A12) and (A13), it follows that

(A14) $$\begin{array}{c} D_{f}\left(P_{X}∥Q_{X}\right)-D_{f}\left(P_{Y}∥Q_{Y}\right)\geq c_{f}\left(\xi_{1},\xi_{2}\right)\sum_{(x,y)\in X\times Y}\left\{Q_{X}\left(x\right)\;W_{Y|X}\left(y|x\right)\;{\left(\frac{P_{X}\left(x\right)}{Q_{X}\left(x\right)}-\frac{P_{Y}\left(y\right)}{Q_{Y}\left(y\right)}\right)}^{2}\right\}, \end{array}$$

and an evaluation of the sum in the right side of (A14) gives (see (20), (21) and (A3))

(A15) $$\begin{array}{c} \sum_{(x,y)\in X\times Y}\left\{Q_{X}\left(x\right)\;W_{Y|X}\left(y|x\right)\;{\left(\frac{P_{X}\left(x\right)}{Q_{X}\left(x\right)}-\frac{P_{Y}\left(y\right)}{Q_{Y}\left(y\right)}\right)}^{2}\right\}\\ =\sum_{x\in X}\left\{\frac{P_{X}^{2}\left(x\right)}{Q_{X}\left(x\right)}\;\underset{=1}{\underset{︸}{\sum_{y\in Y}W_{Y|X}\left(y|x\right)}}\right\}-2\sum_{y\in Y}\left\{\frac{P_{Y}\left(y\right)}{Q_{Y}\left(y\right)}\;\underset{=P_{Y}\left(y\right)}{\underset{︸}{\sum_{x\in X}P_{X}\left(x\right)W_{Y|X}\left(y|x\right)}}\right\}\\ \;+\sum_{y\in Y}\left\{\frac{P_{Y}^{2}\left(y\right)}{Q_{Y}^{2}\left(y\right)}\;\underset{=Q_{Y}\left(y\right)}{\underset{︸}{\sum_{x\in X}Q_{X}\left(x\right)W_{Y|X}\left(y|x\right)}}\right\} \end{array}$$

(A16) $$\begin{array}{c} =\sum_{x\in X}\frac{P_{X}^{2}\left(x\right)}{Q_{X}\left(x\right)}-\sum_{y\in Y}\frac{P_{Y}^{2}\left(y\right)}{Q_{Y}\left(y\right)} \end{array}$$

(A17) $$\begin{array}{c} =\sum_{x\in X}\frac{{\left(P_{X}\left(x\right)-Q_{X}\left(x\right)\right)}^{2}}{Q_{X}\left(x\right)}-\sum_{y\in Y}\frac{{\left(P_{Y}\left(y\right)-Q_{Y}\left(y\right)\right)}^{2}}{Q_{Y}\left(y\right)} \end{array}$$

(A18) $$\begin{array}{c} =\chi^{2}\left(P_{X}∥Q_{X}\right)-\chi^{2}\left(P_{Y}∥Q_{Y}\right). \end{array}$$

Combining (A14)–(A18) gives (24); (25) is due to the data-processing inequality for *f*-divergences (applied to the $\chi^{2}$-divergence), and the non-negativity of $c_{f}\left(\xi_{1},\xi_{2}\right)$ in (22).

The $\chi^{2}$-divergence is an *f*-divergence with $f\left(t\right)={\left(t-1\right)}^{2}$ for t≥0. The condition in (22) allows to set here $c_{f}\left(\xi_{1},\xi_{2}\right)\equiv 1$, implying that (24) holds in this case with equality.

We next prove Item (b). Let *f* be twice differentiable on $I:=I(\xi_{1},\xi_{2})$ (see (23)), and let (u,v)∈I×I with v>u. Dividing both sides of (22) by v−u, and letting $v\to u^{+}$, yields $c_{f}\left(\xi_{1},\xi_{2}\right)\leq \frac{1}{2}\;f^{\prime \prime }\left(u\right)$. Since this holds for all u∈I, it follows that $c_{f}\left(\xi_{1},\xi_{2}\right)\leq \frac{1}{2}\;\underset{t\in I}{\inf}f^{\prime \prime }\left(t\right)$. We next show that $c_{f}\left(\xi_{1},\xi_{2}\right)$ in (26) fulfills the condition in (22), and therefore it is the largest possible value of $c_{f}$ to satisfy (22). By the mean value theorem of Lagrange, for all (u,v)∈I×I with v>u, there exists an intermediate value ξ∈(u,v) such that $f^{\prime }\left(v\right)-f^{\prime }\left(u\right)=f^{\prime \prime }\left(\xi \right)\;\left(v-u\right)$; hence, $f^{\prime }\left(v\right)-f^{\prime }\left(u\right)\geq 2c_{f}\left(\xi_{1},\xi_{2}\right)\;\left(v-u\right)$, so the condition in (22) is indeed fulfilled with $c_{f}:=c_{f}\left(\xi_{1},\xi_{2}\right)$ as given in (26).

We next prove Item (c). Let $f^{*}:\left(0,\infty \right)\to R$ be the dual convex function which is given by $f^{*}\left(t\right):=tf(\frac{1}{t})$ for all t>0 with $f^{*}\left(1\right)=f\left(1\right)=0$. Since $P_{X}$, $P_{Y}$, $Q_{X}$ and $Q_{Y}$ are supported on X (see (A5) and (A6)), we have

(A19) $$\begin{array}{cc} \; & D_{f}\left(P_{X}∥Q_{X}\right)=D_{f^{*}}\left(Q_{X}∥P_{X}\right), \end{array}$$

(A20) $$\begin{array}{cc} \; & D_{f}\left(P_{Y}∥Q_{Y}\right)=D_{f^{*}}\left(Q_{Y}∥P_{Y}\right), \end{array}$$

(A21) $$\begin{array}{cc} \; & \xi_{1}^{*}:=\underset{x\in X}{\inf}\frac{Q_{X}\left(x\right)}{P_{X}\left(x\right)}={\left(\underset{x\in X}{\sup}\frac{P_{X}\left(x\right)}{Q_{X}\left(x\right)}\right)}^{-1}=\frac{1}{\xi_{2}}, \end{array}$$

(A22) $$\begin{array}{cc} \; & \xi_{2}^{*}:=\underset{x\in X}{\sup}\frac{Q_{X}\left(x\right)}{P_{X}\left(x\right)}={\left(\underset{x\in X}{\inf}\frac{P_{X}\left(x\right)}{Q_{X}\left(x\right)}\right)}^{-1}=\frac{1}{\xi_{1}}. \end{array}$$

Consequently, it follows that

(A23) $$\begin{array}{cc} D_{f}\left(P_{X}∥Q_{X}\right)-D_{f}\left(P_{Y}∥Q_{Y}\right)\; & =D_{f^{*}}\left(Q_{X}∥P_{X}\right)-D_{f^{*}}\left(Q_{Y}∥P_{Y}\right) \end{array}$$

(A24) $$\begin{array}{cc} \; & \geq c_{f^{*}}\left(\xi_{1}^{*},\xi_{2}^{*}\right)\left[\chi^{2}\left(Q_{X}∥P_{X}\right)-\chi^{2}\left(Q_{Y}∥P_{Y}\right)\right] \end{array}$$

(A25) $$\begin{array}{cc} \; & =c_{f^{*}}\left(\frac{1}{\xi_{2}},\frac{1}{\xi_{1}}\right)\left[\chi^{2}\left(Q_{X}∥P_{X}\right)-\chi^{2}\left(Q_{Y}∥P_{Y}\right)\right] \end{array}$$

where (A23) holds due to (A19) and (A20); (A24) follows from (24) with *f*, $P_{X}$ and $Q_{X}$ replaced by $f^{*}$, $Q_{X}$ and $P_{X}$, respectively, which then implies that $\xi_{1}$ and $\xi_{2}$ in (18) and (19) are, respectively, replaced by $\xi_{1}^{*}$ and $\xi_{2}^{*}$ in (A21) and (A22); finally, (A25) holds due to (A21) and (A22). Since by assumption *f* is twice differentiable on (0,∞), so is $f^{*}$, and

(A26) $$\begin{array}{c} {\left(f^{*}\right)}^{\prime \prime }\left(t\right)=\frac{1}{t^{3}}\;f\left(\frac{1}{t}\right),\;t>0. \end{array}$$

Hence,

(A27) $$\begin{array}{cc} c_{f^{*}}\left(\frac{1}{\xi_{2}},\frac{1}{\xi_{1}}\right)\; & =\frac{1}{2}\underset{u\in I\left(\frac{1}{\xi_{2}},\frac{1}{\xi_{1}}\right)}{\inf}{\left(f^{*}\right)}^{\prime \prime }\left(u\right) \end{array}$$

(A28) $$\begin{array}{cc} \; & =\frac{1}{2}\underset{u\in I\left(\frac{1}{\xi_{2}},\frac{1}{\xi_{1}}\right)}{\inf}\left\{{\left(\frac{1}{u}\right)}^{3}\;f\left(\frac{1}{u}\right)\right\} \end{array}$$

(A29) $$\begin{array}{cc} \; & =\frac{1}{2}\underset{t\in I(\xi_{1},\xi_{2})}{\inf}\left\{t^{3}f\left(t\right)\right\} \end{array}$$

where (A27) follows from (24) with *f*, $\xi_{1}$ and $\xi_{2}$ replaced by $f^{*}$, $\frac{1}{\xi_{2}}$ and $\frac{1}{\xi_{1}}$, respectively; (A28) holds due to (A26), and (A29) holds by substituting $t=:\frac{1}{u}$. This proves (27) and (30), where (28) is due to the data-processing inequality for *f*-divergences, and the non-negativity of $c_{f^{*}}\left(\cdot ,\cdot \right)$.

Similarly to the condition for equality in (24), equality in (27) is satisfied if $f^{*}\left(t\right)={\left(t-1\right)}^{2}$ for all t>0, or equivalently $f\left(t\right)=tf^{*}(\frac{1}{t})=\frac{{\left(t-1\right)}^{2}}{t}$ for all t>0. This *f*-divergence is Neyman’s $\chi^{2}$-divergence where $D_{f}\left(P∥Q\right):=\chi^{2}\left(Q∥P\right)$ for all *P* and *Q* with $c_{f^{*}}\equiv 1$ (due to (30), and since $t^{3}f^{\prime \prime }\left(t\right)=2$ for all t>0).

The proof of Item (d) follows that same lines as the proof of Items (a)–(c) by replacing the condition in (22) with a complementary condition of the form

(A30) $$\begin{array}{c} f_{+}^{\prime }\left(v\right)-f_{+}^{\prime }\left(u\right)\leq 2e_{f}\left(\xi_{1},\xi_{2}\right)\;\left(v-u\right),\;\forall \;u,v\in I\left(\xi_{1},\xi_{2}\right),\;u<v. \end{array}$$

We finally prove Item (e) by showing that the lower and upper bounds in (24), (27), (32) and (33) are locally tight. More precisely, let $\left\{P_{X}^{\left(n\right)}\right\}$ be a sequence of probability mass functions defined on X and pointwise converging to $Q_{X}$ which is supported on X, let $P_{Y}^{\left(n\right)}$ and $Q_{Y}$ be the probability mass functions defined on Y via (20) and (21) with inputs $P_{X}^{\left(n\right)}$ and $Q_{X}$, respectively, and let $\left\{\xi_{1,n}\right\}$ and $\left\{\xi_{2,n}\right\}$ be defined, respectively, by (18) and (19) with $P_{X}$ being replaced by $P_{X}^{\left(n\right)}$. By the assumptions in (35) and (36),

(A31) $$\begin{array}{cc} \; & \lim_{n\to \infty }\xi_{1,n}=\lim_{n\to \infty }\underset{x\in X}{\inf}\frac{P_{X}^{\left(n\right)}\left(x\right)}{Q_{X}\left(x\right)}=1, \end{array}$$

(A32) $$\begin{array}{cc} \; & \lim_{n\to \infty }\xi_{2,n}=\lim_{n\to \infty }\underset{x\in X}{\sup}\frac{P_{X}^{\left(n\right)}\left(x\right)}{Q_{X}\left(x\right)}=1. \end{array}$$

Consequently, if *f* has a continuous second derivative at unity, then (24), (26), (31), (32), (A31) and (A32) imply that

(A33) $$\begin{array}{c} \lim_{n\to \infty }\frac{D_{f}\left(P_{X}^{\left(n\right)}∥Q_{X}\right)-D_{f}\left(P_{Y}^{\left(n\right)}∥Q_{Y}\right)}{\chi^{2}\left(P_{X}^{\left(n\right)}∥Q_{X}\right)-\chi^{2}\left(P_{Y}^{\left(n\right)}∥Q_{Y}\right)}\\ =\lim_{n\to \infty }c_{f}\left(\xi_{1,n},\xi_{2,n}\right)=\lim_{n\to \infty }e_{f}\left(\xi_{1,n},\xi_{2,n}\right)=\frac{1}{2}f^{\prime \prime }\left(1\right), \end{array}$$

and similarly, from (27), (30), (33), (34), (A31) and (A32),

(A34) $$\begin{array}{c} \lim_{n\to \infty }\frac{D_{f}\left(P_{X}^{\left(n\right)}∥Q_{X}\right)-D_{f}\left(P_{Y}^{\left(n\right)}∥Q_{Y}\right)}{\chi^{2}\left(Q_{X}∥P_{X}^{\left(n\right)}\right)-\chi^{2}\left(Q_{Y}∥P_{Y}^{\left(n\right)}\right)}\\ =\lim_{n\to \infty }c_{f^{*}}\left(\frac{1}{\xi_{2,n}},\frac{1}{\xi_{1,n}}\right)=\lim_{n\to \infty }e_{f^{*}}\left(\frac{1}{\xi_{2,n}},\frac{1}{\xi_{1,n}}\right)=\frac{1}{2}f^{\prime \prime }\left(1\right), \end{array}$$

which, respectively, prove (37) and (38).

## Appendix B. Proof of Theorem 2

We start by proving Item (a). By the assumption that $P_{X_{i}}$ and $Q_{X_{i}}$ are supported on X for all i∈{1,…,n}, it follows from (39) that the probability mass functions $P_{X^{n}}$ and $Q_{X^{n}}$ are supported on $X^{n}$. Consequently, from (41), also $R_{X^{n}}^{\left(\lambda \right)}$ is supported on $X^{n}$ for all λ∈[0,1]. Due to the product forms of $Q_{X^{n}}$ and $R_{X^{n}}^{\left(\lambda \right)}$ in (39) and (41), respectively, we get from (47) that

(A35) $$\begin{array}{cc} \xi_{1}\left(n,\lambda \right) & =\prod_{i=1}^{n}\left(1-\lambda +\lambda \;\underset{x\in X}{\inf}\frac{P_{X_{i}}\left(x\right)}{Q_{X_{i}}\left(x\right)}\right)\\ & =\prod_{i=1}^{n}\left(\underset{x\in X}{\inf}\frac{\lambda P_{X_{i}}\left(x\right)+\left(1-\lambda \right)Q_{X_{i}}\left(x\right)}{Q_{X_{i}}\left(x\right)}\right)\\ & =\underset{\underline{x}\in X^{n}}{\inf}\left\{\frac{\underset{i=1}{\prod^{n}}\left(\lambda P_{X_{i}}\left(x_{i}\right)+\left(1-\lambda \right)Q_{X_{i}}\left(x_{i}\right)\right)}{\underset{i=1}{\prod^{n}}Q_{X_{i}}\left(x_{i}\right)}\right\}\\ & =\underset{\underline{x}\in X^{n}}{\inf}\frac{R_{X^{n}}^{\left(\lambda \right)}\left(\underline{x}\right)}{Q_{X^{n}}\left(\underline{x}\right)}\in \left(0,1\right], \end{array}$$

and likewise, from (48),

(A36) $$\begin{array}{cc} \xi_{2}\left(n,\lambda \right)\; & =\underset{\underline{x}\in X^{n}}{\sup}\frac{R_{X^{n}}^{\left(\lambda \right)}\left(\underline{x}\right)}{Q_{X^{n}}\left(\underline{x}\right)}\in \left[1,\infty \right) \end{array}$$

for all λ∈[0,1]. In view of (24), (26), (A35) and (A36), replacing $\left(P_{X},\;P_{Y},\;Q_{X},\;Q_{Y},\;\xi_{1},\;\xi_{2}\right)$ in (24) and (26) with $(R_{X^{n}}^{\left(\lambda \right)},\;R_{Y^{n}}^{\left(\lambda \right)},\;Q_{X^{n}},\;Q_{Y^{n}},\;\xi_{1}\left(n,\lambda \right),\;\xi_{2}\left(n,\lambda \right)),$ we obtain that, for all λ∈[0,1],

(A37) $$\begin{array}{c} D_{f}\left(R_{X^{n}}^{\left(\lambda \right)}\;∥\;Q_{X^{n}}\right)-D_{f}\left(R_{Y^{n}}^{\left(\lambda \right)}\;∥\;Q_{Y^{n}}\right)\\ \geq c_{f}\left(\xi_{1}\left(n,\lambda \right),\xi_{2}\left(n,\lambda \right)\right)\left[\chi^{2}\left(R_{X^{n}}^{\left(\lambda \right)}\;∥\;Q_{X^{n}}\right)-\chi^{2}\left(R_{Y^{n}}^{\left(\lambda \right)}\;∥\;Q_{Y^{n}}\right)\right]. \end{array}$$

Due to the setting in (39)–(44), for all $\underline{y}\in Y^{n}$ and λ∈[0,1],

(A38) $$\begin{array}{cc} R_{Y^{n}}^{\left(\lambda \right)}\left(\underline{y}\right) & =\sum_{\underline{x}\in X^{n}}R_{X^{n}}^{\left(\lambda \right)}\left(\underline{x}\right)\;W_{Y^{n}|X^{n}}\left(\underline{y}|\underline{x}\right)\\ & =\sum_{\underline{x}\in X^{n}}\left\{\prod_{i=1}^{n}\left(\lambda P_{X_{i}}\left(x_{i}\right)+\left(1-\lambda \right)Q_{X_{i}}\left(x_{i}\right)\right)\;\prod_{i=1}^{n}W_{Y_{i}|X_{i}}\left(y_{i}|x_{i}\right)\right\}\\ & =\prod_{i=1}^{n}\left\{\sum_{x_{i}\in X}\left\{\left(\lambda P_{X_{i}}\left(x_{i}\right)+\left(1-\lambda \right)Q_{X_{i}}\left(x_{i}\right)\right)\;W_{Y_{i}|X_{i}}\left(y_{i}|x_{i}\right)\right\}\right\}\\ & =\prod_{i=1}^{n}\left\{\lambda \sum_{x\in X}P_{X_{i}}\left(x\right)W_{Y_{i}|X_{i}}\left(y_{i}|x\right)+\left(1-\lambda \right)\sum_{x\in X}Q_{X_{i}}\left(x\right)W_{Y_{i}|X_{i}}\left(y_{i}|x\right)\right\}\\ & =\prod_{i=1}^{n}\left(\lambda P_{Y_{i}}\left(y_{i}\right)+\left(1-\lambda \right)Q_{Y_{i}}\left(y_{i}\right)\right)\\ & =\prod_{i=1}^{n}R_{Y_{i}}^{\left(\lambda \right)}\left(y_{i}\right) \end{array}$$

with

(A39) $$\begin{array}{c} R_{Y_{i}}^{\left(\lambda \right)}\left(y\right):=\lambda P_{Y_{i}}\left(y\right)+\left(1-\lambda \right)Q_{Y_{i}}\left(y\right),\;\forall \;i\in \left\{1,\ldots ,n\right\},\;y\in Y,\;\lambda \in \left[0,1\right], \end{array}$$

and $R_{Y_{i}}^{\left(\lambda \right)}$ is the probability mass function at the channel output at time instant *i*. In particular, setting λ=0 in (A38) gives

(A40) $$\begin{array}{c} Q_{Y^{n}}\left(\underline{y}\right)=\prod_{i=1}^{n}Q_{Y_{i}}\left(y_{i}\right),\;\forall \;\underline{y}\in Y^{n}. \end{array}$$

Due to the tensorization property of the $\chi^{2}$ divergence, and since $R_{X^{n}}^{\left(\lambda \right)}$, $R_{Y^{n}}^{\left(\lambda \right)}$, $Q_{X^{n}}$ and $Q_{Y^{n}}$ are product probability measures (see (39), (41), (A38) and (A40)), it follows that

(A41) $$\begin{array}{c} \chi^{2}\left(R_{X^{n}}^{\left(\lambda \right)}\;∥\;Q_{X^{n}}\right)=\prod_{i=1}^{n}\left(1+\chi^{2}(R_{X_{i}}^{\left(\lambda \right)}\;∥\;Q_{X_{i}}\right))-1, \end{array}$$

and

(A42) $$\begin{array}{c} \chi^{2}\left(R_{Y^{n}}^{\left(\lambda \right)}\;∥\;Q_{Y^{n}}\right)=\prod_{i=1}^{n}\left(1+\chi^{2}(R_{Y_{i}}^{\left(\lambda \right)}\;∥\;Q_{Y_{i}}\right))-1. \end{array}$$

Substituting (A41) and (A42) into the right side of (A37) gives that, for all λ∈[0,1],

(A43) $$\begin{array}{c} D_{f}\left(R_{X^{n}}^{\left(\lambda \right)}\;∥\;Q_{X^{n}}\right)-D_{f}\left(R_{Y^{n}}^{\left(\lambda \right)}\;∥\;Q_{Y^{n}}\right)\\ \geq c_{f}\left(\xi_{1}\left(n,\lambda \right),\xi_{2}\left(n,\lambda \right)\right)\left[\;\prod_{i=1}^{n}\left(1+\chi^{2}(R_{X_{i}}^{\left(\lambda \right)}\;∥\;Q_{X_{i}}\right))-\prod_{i=1}^{n}\left(1+\chi^{2}(R_{Y_{i}}^{\left(\lambda \right)}\;∥\;Q_{Y_{i}}\right))\right]. \end{array}$$

Due to (41) and (A39), since

(A44) $$\begin{array}{cc} \; & R_{X_{i}}^{\left(\lambda \right)}=\lambda P_{X_{i}}+\left(1-\lambda \right)Q_{X_{i}}, \end{array}$$

(A45) $$\begin{array}{cc} \; & R_{Y_{i}}^{\left(\lambda \right)}=\lambda P_{Y_{i}}+\left(1-\lambda \right)Q_{Y_{i}}, \end{array}$$

and (see ([45], Lemma 5))

(A46) $$\begin{array}{c} \chi^{2}\left(\lambda P+\left(1-\lambda \right)Q\;∥\;Q\right)=\lambda^{2}\;\chi^{2}\left(P∥Q\right),\;\forall \;\lambda \in \left[0,1\right] \end{array}$$

for every pair of probability measures (P,Q), it follows that

(A47) $$\begin{array}{cc} \; & \chi^{2}(R_{X_{i}}^{\left(\lambda \right)}\;∥\;Q_{X_{i}})=\lambda^{2}\;\chi^{2}\left(P_{X_{i}}\;∥\;Q_{X_{i}}\right), \end{array}$$

(A18) $$\begin{array}{cc} \; & \chi^{2}(R_{Y_{i}}^{\left(\lambda \right)}\;∥\;Q_{Y_{i}})=\lambda^{2}\;\chi^{2}\left(P_{Y_{i}}\;∥\;Q_{Y_{i}}\right). \end{array}$$

Substituting (A47) and (A48) into the right side of (A43) gives (45). For proving the looser bound (46) from (45), and also for later proving the result in Item (c), we rely on the following lemma.

**Lemma** **A1.**
*Let ${\left\{a_{i}\right\}}_{i=1}^{n}$ and ${\left\{b_{i}\right\}}_{i=1}^{n}$ be non-negative with $a_{i}\geq b_{i}$ for all i∈{1,…,n}. Then,*

(a) *For all u≥0,*
  (A49) $$\begin{array}{c} \prod_{i=1}^{n}\left(1+a_{i}u\right)-\prod_{i=1}^{n}\left(1+b_{i}u\right)\geq \sum_{i=1}^{n}\left(a_{i}-b_{i}\right)u. \end{array}$$
(b) *If $a_{i}>b_{i}$ for at least one index i, then*
  (A50) $$\begin{array}{c} \prod_{i=1}^{n}\left(1+a_{i}u\right)-\prod_{i=1}^{n}\left(1+b_{i}u\right)=\sum_{i=1}^{n}\left(a_{i}-b_{i}\right)u+O\left(u^{2}\right). \end{array}$$

**Proof.** Let g:[0,∞)→R be defined as

(A51) $$\begin{array}{c} g\left(u\right):=\prod_{i=1}^{n}\left(1+a_{i}u\right)-\prod_{i=1}^{n}\left(1+b_{i}u\right),\;\forall \;u\geq 0. \end{array}$$

We have g(0)=0, and the first two derivatives of *g* are given by

(A52) $$\begin{array}{c} g^{\prime }\left(u\right)=\sum_{i=1}^{n}\left\{a_{i}\prod_{j\neq i}\left(1+a_{j}u\right)-b_{i}\prod_{j\neq i}\left(1+b_{j}u\right)\right\}, \end{array}$$

and

(A53) $$\begin{array}{c} g^{\prime \prime }\left(u\right)=\sum_{i=1}^{n}\sum_{j\neq i}\left\{a_{i}a_{j}\prod_{k\neq i,j}\left(1+a_{k}u\right)-b_{i}b_{j}\prod_{k\neq i,j}\left(1+b_{k}u\right)\right\}. \end{array}$$

Since by assumption $a_{i}\geq b_{i}\geq 0$ for all *i*, it follows from (A53) that $g^{\prime \prime }\left(u\right)\geq 0$ for all u≥0, which asserts the convexity of *g* on [0,∞). Hence, for all u≥0,

(A54) $$\begin{array}{c} g\left(u\right)\geq g\left(0\right)+g^{\prime }\left(0\right)u=\sum_{i=1}^{n}\left(b_{i}-a_{i}\right)u \end{array}$$

where the right-side equality in (A54) is due to (A51) and (A52). This gives (A49). We next prove Item (b) of Lemma A1. By the Taylor series expansion of the polynomial function *g*, we get

(A55) $$\begin{array}{cc} g(u) & =g\left(0\right)+g^{\prime }\left(0\right)u+\frac{1}{2}g^{\prime \prime }\left(0\right)u^{2}+\ldots \\ & =\sum_{i=1}^{n}\left(b_{i}-a_{i}\right)u+\frac{1}{2}\sum_{i=1}^{n}\sum_{j\neq i}\left(a_{i}a_{j}-b_{i}b_{j}\right)u^{2}+\ldots \end{array}$$

for all u≥0. Since by assumption $a_{i}\geq b_{i}\geq 0$ for all *i*, and there exists an index i∈{1,…,n} such that $a_{i}>b_{i}$, it follows that the coefficient of $u^{2}$ in the right side of (A55) is positive. This yields (A50).  □

We obtain here (46) from (45) and Item (a) of Lemma A1. To that end, for i∈{1,…,n}, let

(A56) $$\begin{array}{c} a_{i}:=\chi^{2}\left(P_{X_{i}}∥Q_{X_{i}}\right),\;b_{i}:=\chi^{2}\left(P_{Y_{i}}∥Q_{Y_{i}}\right),\;u:=\lambda^{2} \end{array}$$

with u∈[0,1] for every λ∈[0,1]. Since by (39), (40), (43) and (44),

(A57) $$\begin{array}{cc} \; & P_{X_{i}}\to W_{Y_{i}|X_{i}}\to P_{Y_{i}}, \end{array}$$

(A58) $$\begin{array}{cc} \; & Q_{X_{i}}\to W_{Y_{i}|X_{i}}\to Q_{Y_{i}}, \end{array}$$

it follows from the data-processing inequality for *f*-divergences, and their non-negativity, that

(A59) $$\begin{array}{c} a_{i}\geq b_{i}\geq 0,\;\forall \;i\in \left\{1,\ldots ,n\right\}, \end{array}$$

which yields (46) from (45), (A49), (A56) and (A59).

We next prove Item (b) of Theorem 2. Similarly to the proof of (A37), we get from (32) (rather than (24)) that

(A60) $$\begin{array}{c} D_{f}\left(R_{X^{n}}^{\left(\lambda \right)}\;∥\;Q_{X^{n}}\right)-D_{f}\left(R_{Y^{n}}^{\left(\lambda \right)}\;∥\;Q_{Y^{n}}\right)\\ \leq e_{f}\left(\xi_{1}\left(n,\lambda \right),\xi_{2}\left(n,\lambda \right)\right)\left[\chi^{2}\left(R_{X^{n}}^{\left(\lambda \right)}\;∥\;Q_{X^{n}}\right)-\chi^{2}\left(R_{Y^{n}}^{\left(\lambda \right)}\;∥\;Q_{Y^{n}}\right)\right]. \end{array}$$

Combining (A41), (A42), (A47), (A48) and (A60) gives (49).

We finally prove Item (c) of Theorem 2. In view of (47) and (48), and by the assumption that $\underset{x\in X}{\sup}\frac{P_{X_{i}}\left(x\right)}{Q_{X_{i}}\left(x\right)}<\infty$ for all i∈{1,…,n}, we get

(A61) $$\begin{array}{cc} \; & \lim_{\lambda \to 0^{+}}\xi_{1}\left(n,\lambda \right)=1, \end{array}$$

(A62) $$\begin{array}{cc} \; & \lim_{\lambda \to 0^{+}}\xi_{2}\left(n,\lambda \right)=1. \end{array}$$

Since, by assumption *f* has a continuous second derivative at unity, (26), (31), (A61) and (A62) imply that

(A63) $$\begin{array}{cc} \; & \lim_{\lambda \to 0^{+}}c_{f}\left(\xi_{1}\left(n,\lambda \right),\xi_{2}\left(n,\lambda \right)\right)=\frac{1}{2}f^{\prime \prime }\left(1\right), \end{array}$$

(A64) $$\begin{array}{cc} \; & \lim_{\lambda \to 0^{+}}e_{f}\left(\xi_{1}\left(n,\lambda \right),\xi_{2}\left(n,\lambda \right)\right)=\frac{1}{2}f^{\prime \prime }\left(1\right). \end{array}$$

From (A56), (A59), and Item (b) of Lemma A1, it follows that

(A65) $$\begin{array}{c} \lim_{\lambda \to 0^{+}}\;\frac{1}{\lambda^{2}}\left[\;\prod_{i=1}^{n}\left(1+\lambda^{2}\;\chi^{2}\left(P_{X_{i}}∥Q_{X_{i}}\right)\right)-\prod_{i=1}^{n}\left(1+\lambda^{2}\;\chi^{2}\left(P_{Y_{i}}∥Q_{Y_{i}}\right)\right)\right]\\ =\sum_{i=1}^{n}\left[\chi^{2}\left(P_{X_{i}}∥Q_{X_{i}}\right)-\chi^{2}\left(P_{Y_{i}}∥Q_{Y_{i}}\right)\right]. \end{array}$$

The result in (50) finally follows from (45), (49) and (A63)–(A65). This indeed shows that the lower bounds in the right sides of (45) and (46), and the upper bound in the right side of (49) yield a tight result as we let $\lambda \to 0^{+}$, leading to the limit in the right side of (50).

## Appendix C. Proof of Theorems 3 and 4

### Appendix C.1. Proof of Theorem 3

We first obtain a lower bound on $D_{f}\left(P_{X}∥Q_{X}\right)$, and then obtain an upper bound on $D_{f}\left(P_{Y}∥Q_{Y}\right)$.

(A66) $$\begin{array}{cc} D_{f}\left(P_{X}∥Q_{X}\right) & =\sum_{x\in X}Q_{X}\left(x\right)\;f\;\left(\frac{P_{X}\left(x\right)}{Q_{X}\left(x\right)}\right) \end{array}$$

(A67) $$\begin{array}{cc} \; & =\sum_{x\in X}Q_{X}\left(x\right)\;\left[\frac{P_{X}\left(x\right)}{Q_{X}\left(x\right)}\;g\;\left(\frac{P_{X}\left(x\right)}{Q_{X}\left(x\right)}\right)+f\left(0\right)\right] \end{array}$$

(A68) $$\begin{array}{cc} \; & =f\left(0\right)+\sum_{x\in X}P_{X}\left(x\right)\;g\;\left(\frac{P_{X}\left(x\right)}{Q_{X}\left(x\right)}\right) \end{array}$$

(A69) $$\begin{array}{cc} \; & \geq f\left(0\right)+g\left(\;\sum_{x\in X}\frac{P_{X}^{2}\left(x\right)}{Q_{X}\left(x\right)}\right) \end{array}$$

(A70) $$\begin{array}{cc} \; & =f\left(0\right)+g\left(1+\chi^{2}\left(P_{X}∥Q_{X}\right)\right) \end{array}$$

(A71) $$\begin{array}{cc} \; & \geq f\left(0\right)+g\left(1\right)+g^{\prime }\left(1\right)\;\chi^{2}\left(P_{X}∥Q_{X}\right) \end{array}$$

(A72) $$\begin{array}{cc} \; & =g^{\prime }\left(1\right)\;\chi^{2}\left(P_{X}∥Q_{X}\right) \end{array}$$

(A73) $$\begin{array}{cc} \; & =\left(f^{\prime }\left(1\right)+f\left(0\right)\right)\;\chi^{2}\left(P_{X}∥Q_{X}\right), \end{array}$$

where (A67) holds by the definition of *g* in Theorem 3 with the assumption that f(0)<∞; (A69) is due to Jensen’s inequality and the convexity of *g*; (A70) holds by the definition of the $\chi^{2}$-divergence; (A71) holds due to the convexity of *g*, and its differentiability at 1 (due to the differentiability of *f* at 1); (A72) holds since f(0)+g(1)=f(1)=0; finally, (A73) holds since f(1)=0 implies that $g^{\prime }\left(1\right)=f^{\prime }\left(1\right)+f\left(0\right)$.

By ([62], Theorem 5), it follows that

(A74) $$\begin{array}{c} D_{f}\left(P_{Y}∥Q_{Y}\right)\leq \kappa \left(\xi_{1},\xi_{2}\right)\;\chi^{2}\left(P_{Y}∥Q_{Y}\right), \end{array}$$

where $\kappa (\xi_{1},\xi_{2})$ is given in (51). Combining (A66)–(A74) yields (52). Taking suprema on both sides of (52), with respect to all probability mass functions $P_{X}$ with $P_{X}\ll Q_{X}$ and $P_{X}\neq Q_{X}$, gives (53) since by the definition of $\kappa (\xi_{1},\xi_{2})$ in (51), it is monotonically decreasing in $\xi_{1}\in \left[0,1\right)$ and monotonically increasing in $\xi_{2}\in \left(1,\infty \right]$, while (18) and (19) yield

(A75) $$\begin{array}{c} \xi_{1}\geq 0,\;\xi_{2}\leq \frac{1}{\min_{x\in X}\;Q_{X}\left(x\right)}. \end{array}$$

**Remark** **A1.** *The derivation in* (A66)*–*(A73) *is conceptually similar to the proof of ([24], Lemma A.2). However, the function g here is convex, and our derivation involves the $\chi^{2}$-divergence.*

**Remark** **A2.** *The proof of ([26], Theorem 8) (see Proposition 3 in Section 1.1 here) relies on ([24], Lemma A.2), where the function g is required to be concave in [24,26]. This leads, in the proof of ([26], Theorem 8), to an upper bound on $D_{f}\left(P_{Y}∥Q_{Y}\right)$. One difference in the derivation of Theorem 3 is that our requirement on the convexity of g leads to a lower bound on $D_{f}\left(P_{X}∥Q_{X}\right)$, instead of an upper bound on $D_{f}\left(P_{Y}∥Q_{Y}\right)$. Another difference between the proofs of Theorem 3 and ([26], Theorem 8) is that we apply here the result in ([62], Theorem 5) to obtain an upper bound on $D_{f}\left(P_{Y}∥Q_{Y}\right)$, whereas the proof of ([26], Theorem 8) relies on a Pinsker-type inequality (see ([63], Theorem 3)) to obtain a lower bound on $D_{f}\left(P_{X}∥Q_{X}\right)$; the latter lower bound relies on the condition on f in* (16)*, which is not necessary for the derivation of the bound in Theorem 3.*

**Remark** **A3.** *From ([62], Theorem 1 (b)), it follows that*

(A76) $$\begin{array}{c} \underset{P\neq Q}{\sup}\frac{D_{f}\left(P∥Q\right)}{\chi^{2}\left(P∥Q\right)}=\kappa \left(\xi_{1},\xi_{2}\right), \end{array}$$

*with $\kappa (\xi_{1},\xi_{2})$ in the right side of* (A76) *as given in* (51)*, and the supremum in the left side of* (A76) *is taken over all probability measures P and Q such that P≠Q. In view of ([62], Theorem 1 (b)), the equality in* (A76) *holds since the functions $\tilde{f},\tilde{g}:\left(0,\infty \right)\to R$, defined as $\tilde{f}\left(t\right):=f\left(t\right)+f^{\prime }\left(1\right)\left(1-t\right)$ and $\tilde{g}\left(t\right):={\left(t-1\right)}^{2}$ for all t>0, satisfy $D_{\tilde{f}}\left(P∥Q\right)=D_{f}\left(P∥Q\right)$ and $D_{\tilde{g}}\left(P∥Q\right)=\chi^{2}\left(P∥Q\right)$ for all probability measures P and Q, and since ${\tilde{f}}^{\prime }\left(1\right)=\tilde{g}{\;}^{\prime }\left(1\right)=0$ while the function $\tilde{g}$ is also strictly positive on (0,1)∪(1,∞). Furthermore, from the proof of ([62], Theorem 1 (b)), restricting P and Q to be probability mass functions which are defined over a binary alphabet, the ratio $\frac{D_{f}\left(P∥Q\right)}{\chi^{2}\left(P∥Q\right)}$ can be made arbitrarily close to the supremum in the left side of* (A76)*; such probability measures can be obtained as the output distributions $P_{Y}$ and $Q_{Y}$ of an arbitrary non-degenerate stochastic transformation $W_{Y|X}:X\to Y$, with |Y|=2, by a suitable selection of probability input distributions $P_{X}$ and $Q_{X}$, respectively (see (A5) and (A6)). In the latter case where |Y|=2, this shows the optimality of the non-negative constant $\kappa (\xi_{1},\xi_{2})$ in the right side of* (A74)*.*

### Appendix C.2. Proof of Theorem 4

Combining (A66)–(A73) gives that, for all λ∈[0,1],

(A77) $$\begin{array}{c} D_{f}\left(R_{X^{n}}^{\left(\lambda \right)}\;∥\;Q_{X^{n}}^{\left(\lambda \right)}\right)\geq \left(f^{\prime }\left(1\right)+f\left(0\right)\right)\;\chi^{2}\left(R_{X^{n}}^{\left(\lambda \right)}\;∥\;Q_{X^{n}}\right), \end{array}$$

and from (A74)

(A78) $$\begin{array}{c} D_{f}\left(R_{Y^{n}}^{\left(\lambda \right)}\;∥\;Q_{Y^{n}}\right)\leq \kappa \left(\xi_{1}\left(n,\lambda \right),\xi_{2}\left(n,\lambda \right)\right)\;\chi^{2}\left(R_{Y^{n}}^{\left(\lambda \right)}\;∥\;Q_{Y^{n}}\right). \end{array}$$

From (A41) and (A47),

(A79) $$\begin{array}{c} \chi^{2}\left(R_{X^{n}}^{\left(\lambda \right)}\;∥\;Q_{X^{n}}\right)=\prod_{i=1}^{n}\left(1+\lambda^{2}\chi^{2}(P_{X_{i}}\;∥\;Q_{X_{i}}\right))-1, \end{array}$$

and similarly, from (A42) and (A48),

(A80) $$\begin{array}{c} \chi^{2}\left(R_{Y^{n}}^{\left(\lambda \right)}\;∥\;Q_{Y^{n}}\right)=\prod_{i=1}^{n}\left(1+\lambda^{2}\chi^{2}(P_{Y_{i}}\;∥\;Q_{Y_{i}}\right))-1. \end{array}$$

Combining (A77)–(A80) yields (54).

## Appendix D. Proof of Theorem 5

The function $f_{\alpha }:\left[0,\infty \right)\to R$ in (55) satisfies $f_{\alpha }\left(1\right)=0$, and for all $\alpha \geq e^{-\frac{3}{2}}$

(A81) $$\begin{array}{cc} \; & f_{\alpha }^{\prime \prime }\left(t\right)=2\log\left(\alpha +t\right)+3\log e>0,\;\forall \;t>0, \end{array}$$

which yields the convexity of $f_{\alpha }\left(\cdot \right)$ on [0,∞). This justifies the definition of the *f*-divergence

(A82) $$\begin{array}{cc} D_{f_{\alpha }}\left(P∥Q\right) & :=\sum_{x\in X}Q\left(x\right)\;f_{\alpha }\left(\frac{P(x)}{Q(x)}\right) \end{array}$$

for probability mass functions *P* and *Q*, which are defined on a finite or countably infinite set X, with *Q* supported on X. In the general alphabet setting, sums and probability mass functions are, respectively, replaced by Lebesgue integrals and Radon-Nikodym derivatives. Differentiation of both sides of (A82) with respect to α gives

(A83) $$\begin{array}{c} \frac{\partial }{\partial \alpha }\left\{D_{f_{\alpha }}\left(P∥Q\right)\right\}=\sum_{x\in X}Q\left(x\right)\;r_{\alpha }\left(\frac{P(x)}{Q(x)}\right) \end{array}$$

where

(A84) $$\begin{array}{cc} r_{\alpha }\left(t\right) & :=\frac{\partial f_{\alpha }\left(t\right)}{\partial \alpha } \end{array}$$

(A85) $$\begin{array}{cc} \; & =2(\alpha +t)\log(\alpha +t)-2(\alpha +1)\log(\alpha +1)+(t-1)\log e,\;t>0. \end{array}$$

The function $r_{\alpha }:\left(0,\infty \right)\to R$ is convex since

(A86) $$\begin{array}{c} r_{\alpha }^{\prime \prime }\left(t\right)=\frac{2\log e}{\alpha +t}>0,\;\forall \;t>0, \end{array}$$

and $r_{\alpha }\left(1\right)=0$. Hence, $D_{r_{\alpha }}\left(\cdot ∥\cdot \right)$ is an *f*-divergence, and it follows from (A83)–(A85) that

(A87) $$\begin{array}{cc} \frac{\partial }{\partial \alpha }\left\{D_{f_{\alpha }}\left(P∥Q\right)\right\}\; & =D_{r_{\alpha }}\left(P∥Q\right) \end{array}$$

(A88) $$\begin{array}{cc} \; & =2\sum_{x\in X}\left\{\left(\alpha Q(x)+P(x)\right)\;\log\left(\alpha +\frac{P(x)}{Q(x)}\right)\right\}-2\left(\alpha +1\right)\log\left(\alpha +1\right) \end{array}$$

(A89) $$\begin{array}{cc} \; & =2\left(\alpha +1\right)\sum_{x\in X}\frac{\alpha Q(x)+P(x)}{\alpha +1}\;\log\left(\frac{\alpha Q(x)+P(x)}{\left(\alpha +1)\;Q(x\right)}\right) \end{array}$$

(A90) $$\begin{array}{cc} \; & =2\left(\alpha +1\right)\;D\left(\frac{\alpha Q+P}{\alpha +1}\;∥\;Q\right)\geq 0, \end{array}$$

which gives (56), so $D_{f_{\alpha }}\left(\cdot ∥\cdot \right)$ is monotonically increasing in α. Double differentiation of both sides of (A82) with respect to α gives

(A91) $$\begin{array}{c} \frac{\partial^{2}}{\partial \alpha^{2}}\left\{D_{f_{\alpha }}\left(P∥Q\right)\right\}=\sum_{x\in X}Q\left(x\right)\;v_{\alpha }\left(\frac{P(x)}{Q(x)}\right) \end{array}$$

where

(A92) $$\begin{array}{cc} v_{\alpha }\left(t\right) & :=\frac{\partial^{2}f_{\alpha }\left(t\right)}{\partial \alpha^{2}} \end{array}$$

(A93) $$\begin{array}{cc} \; & \;=2\log(\alpha +t)-2\log(\alpha +1),\;t>0. \end{array}$$

The function $v_{\alpha }:\left(0,\infty \right)\to R$ is concave, and $v_{\alpha }\left(1\right)=0$. By referring to the *f*-divergence $D_{-v_{\alpha }}\left(\cdot ∥\cdot \right)$, it follows from (A91)–(A93) that

(A94) $$\begin{array}{cc} \frac{\partial^{2}}{\partial \alpha^{2}}\left\{D_{f_{\alpha }}\left(P∥Q\right)\right\}\; & =-D_{-v_{\alpha }}\left(P∥Q\right) \end{array}$$

(A95) $$\begin{array}{cc} \; & =-2\sum_{x\in X}Q\left(x\right)\left[\log\left(\alpha +1\right)-\log\left(\alpha +\frac{P(x)}{Q(x)}\right)\right] \end{array}$$

(A96) $$\begin{array}{cc} \; & =-2\sum_{x\in X}Q\left(x\right)\log\left(\frac{\left(\alpha +1)Q(x\right)}{\alpha Q(x)+P(x)}\right) \end{array}$$

(A97) $$\begin{array}{cc} \; & =-2\;D\left(Q\;∥\;\frac{\alpha Q+P}{\alpha +1}\right)\leq 0, \end{array}$$

which gives (57), so $D_{f_{\alpha }}\left(\cdot ∥\cdot \right)$ is concave in α for $\alpha \geq e^{-\frac{3}{2}}$. Differentiation of both sides of (A93) with respect to α gives that

(A98) $$\begin{array}{cc} \frac{\partial^{3}f_{\alpha }\left(t\right)}{\partial \alpha^{3}} & =2\left(\frac{1}{\alpha +t}-\frac{1}{\alpha +1}\right)\log e, \end{array}$$

which implies that

(A99) $$\begin{array}{cc} \frac{\partial^{3}}{\partial \alpha^{3}}\left\{D_{f_{\alpha }}\left(P∥Q\right)\right\}\; & =2\log e\;\sum_{x\in X}Q\left(x\right)\left(\frac{1}{\alpha +\frac{P(x)}{Q(x)}}-\frac{1}{\alpha +1}\right) \end{array}$$

(A100) $$\begin{array}{cc} \; & =\frac{2\log e}{\alpha +1}\left[\;\sum_{x\in X}\frac{Q^{2}\left(x\right)}{\frac{\alpha Q(x)+P(x)}{\alpha +1}}-1\right] \end{array}$$

(A101) $$\begin{array}{cc} \; & =\frac{2\log e}{\alpha +1}\cdot \chi^{2}\left(Q\;∥\;\frac{\alpha Q+P}{\alpha +1}\right)\geq 0. \end{array}$$

This gives (58), and it completes the proof of Item (a).

We next prove Item (b). From Item (a), the result in (59) holds for n=1,2,3. We provide in the following a proof of (59) for all n≥3. In view of (A98), it can be verified that for n≥3,

(A102) $$\begin{array}{c} \frac{\partial^{n}f_{\alpha }\left(t\right)}{\partial \alpha^{n}}=2{\left(-1\right)}^{n-1}\left(n-3\right)!\left[\frac{1}{{\left(\alpha +t\right)}^{n-2}}-\frac{1}{{\left(\alpha +1\right)}^{n-2}}\right]\log e, \end{array}$$

which, from (A82), implies that

(A103) $$\begin{array}{c} {\left(-1\right)}^{n-1}\frac{\partial^{n}}{\partial \alpha^{n}}\left\{D_{f_{\alpha }}\left(P∥Q\right)\right\}=\sum_{x\in X}Q\left(x\right)\;g_{\alpha ,n}\left(\frac{P(x)}{Q(x)}\right) \end{array}$$

with

(A104) $$\begin{array}{cc} g_{\alpha ,n}\left(t\right) & :={\left(-1\right)}^{n-1}\;\frac{\partial^{n}f_{\alpha }\left(t\right)}{\partial \alpha^{n}} \end{array}$$

(A105) $$\begin{array}{cc} \; & =2\left(n-3\right)!\left[\frac{1}{{\left(\alpha +t\right)}^{n-2}}-\frac{1}{{\left(\alpha +1\right)}^{n-2}}\right]\log e,\;t>0. \end{array}$$

The function $g_{\alpha ,n}:\left(0,\infty \right)\to R$ is convex for n≥3, with $g_{\alpha ,n}\left(1\right)=0$. By referring to the *f*-divergence $D_{g_{\alpha ,n}}\left(\cdot ∥\cdot \right)$, its non-negativity and (A103) imply that for all n≥3

(A106) $$\begin{array}{cc} {\left(-1\right)}^{n-1}\frac{\partial^{n}}{\partial \alpha^{n}}\left\{D_{f_{\alpha }}\left(P∥Q\right)\right\}\; & =D_{g_{\alpha ,n}}\left(P∥Q\right)\geq 0. \end{array}$$

Furthermore, we get the following explicit formula for *n*-th partial derivative of $D_{f_{\alpha }}\left(P∥Q\right)$ with respect to α for n≥3:

(A107) $$\begin{array}{cc} \frac{\partial^{n}}{\partial \alpha^{n}}\left\{D_{f_{\alpha }}\left(P∥Q\right)\right\}\; & ={\left(-1\right)}^{n-1}\;\sum_{x\in X}Q\left(x\right)\;g_{\alpha ,n}\left(\frac{P(x)}{Q(x)}\right) \end{array}$$

(A108) $$\begin{array}{cc} \; & =\frac{2{\left(-1\right)}^{n-1}\left(n-3\right)!\;\log e}{{\left(\alpha +1\right)}^{n-2}}\left[\sum_{x\in X}\left\{Q\left(x\right){\left(\frac{\alpha +1}{\alpha +\frac{P(x)}{Q(x)}}\right)}^{n-2}\right\}-1\right] \end{array}$$

(A109) $$\begin{array}{cc} \; & =\frac{2{\left(-1\right)}^{n-1}\left(n-3\right)!\;\log e}{{\left(\alpha +1\right)}^{n-2}}\left[\;\sum_{x\in X}\frac{Q^{n-1}\left(x\right)}{{\left(\frac{\alpha Q(x)+P(x)}{\alpha +1}\right)}^{n-2}}-1\right] \end{array}$$

(A110) $$\begin{array}{cc} \; & =\frac{2{\left(-1\right)}^{n-1}\left(n-3\right)!\;\log e}{{\left(\alpha +1\right)}^{n-2}}\left[\exp\left(\left(n-2\right)\;D_{n-1}\left(Q\;∥\;\frac{\alpha Q+P}{\alpha +1}\right)\right)-1\right] \end{array}$$

where (A107) holds due to (A103); (A108) follows from (A104), and (A110) is satisfied by the definition of the Rényi divergence [40] which is given by

(A111) $$\begin{array}{c} D_{\beta }\left(P∥Q\right):=\frac{1}{\beta -1}\;\log\left(\sum_{x\in X}P^{\beta }\left(x\right)\;Q^{1-\beta }\left(x\right)\right),\;\forall \;\beta \in \left(0,1\right)\cup \left(1,\infty \right) \end{array}$$

with $D_{1}\left(P∥Q\right):=D\left(P∥Q\right)$ by continuous extension of $D_{\beta }\left(\cdot ∥\cdot \right)$ at β=1. For n=3, the right side of (A110) is simplified to the right side of (58); this holds due to the identity

(A112) $$\begin{array}{c} D_{2}\left(P∥Q\right)=\log\left(1+\chi^{2}\left(P∥Q\right)\right). \end{array}$$

To prove Item (c), from (55), for all t≥0

(A113) $$\begin{array}{cc} \; & f_{\alpha }^{\prime }\left(t\right)=2\left(\alpha +t\right)\log\left(\alpha +t\right)+\left(\alpha +t\right)\log e, \end{array}$$

(A114) $$\begin{array}{cc} \; & f_{\alpha }^{\prime \prime }\left(t\right)=2\log\left(\alpha +t\right)+3\log e, \end{array}$$

(A115) $$\begin{array}{cc} \; & f_{\alpha }^{\left(3\right)}\left(t\right)=\frac{2\log e}{\alpha +t}, \end{array}$$

which implies by a Taylor series expansion of $f_{\alpha }\left(\cdot \right)$ that

(A116) $$\begin{array}{c} f_{\alpha }\left(t\right)=f_{\alpha }\left(1\right)+f_{\alpha }^{\prime }\left(1\right)\left(t-1\right)+\frac{1}{2}f_{\alpha }^{\prime \prime }\left(1\right){\left(t-1\right)}^{2}+\frac{1}{6}f_{\alpha }^{\left(3\right)}\left(\xi \right){\left(t-1\right)}^{3},\;\forall \;t\geq 0 \end{array}$$

where ξ in the right side of (A116) is an intermediate value between 1 and *t*. Hence, for t≥0,

(A117) $$\begin{array}{cc} f_{\alpha }\left(t\right) & \geq f_{\alpha }^{\prime }\left(1\right)\left(t-1\right)+\frac{1}{2}f_{\alpha }^{\prime \prime }\left(1\right){\left(t-1\right)}^{2}+\frac{1}{6}f_{\alpha }^{\left(3\right)}\left(0\right){\left(t-1\right)}^{3}\;1\left\{t\in \left[0,1\right]\right\} \end{array}$$

(A118) $$\begin{array}{cc} \; & \geq f_{\alpha }^{\prime }\left(1\right)\left(t-1\right)+\left(\frac{1}{2}f_{\alpha }^{\prime \prime }\left(1\right)-\frac{1}{6}f_{\alpha }^{\left(3\right)}\left(0\right)\right){\left(t-1\right)}^{2} \end{array}$$

(A119) $$\begin{array}{cc} \; & =f_{\alpha }^{\prime }\left(1\right)\left(t-1\right)+k\left(\alpha \right)\;{\left(t-1\right)}^{2} \end{array}$$

where (A117) follows from (A116) since $f_{\alpha }\left(1\right)=0$ and $f_{\alpha }^{\left(3\right)}\left(\cdot \right)$ is monotonically decreasing and positive (see (A115)); 1{t∈[0,1]} in the right side of (A117) denotes the indicator function which is equal to 1 if the relation t∈[0,1] holds, and it is otherwise equal to zero; (A118) holds since ${\left(t-1\right)}^{3}\;1\left\{t\in \left[0,1\right]\right\}\geq -{\left(t-1\right)}^{2}$ for all t≥0, and $f_{\alpha }^{\left(3\right)}\left(0\right)>0$; finally, (A119) follows by substituting (A114) and (A115) into the right side of (A118), which gives the equality

(A120) $$\begin{array}{c} \frac{1}{2}f_{\alpha }^{\prime \prime }\left(1\right)-\frac{1}{6}f_{\alpha }^{\left(3\right)}\left(0\right)=k\left(\alpha \right) \end{array}$$

with k(·) as defined in (63). Since the first term in the right side of (A119) does not affect an *f*-divergence (as it is equal to c(t−1) for t≥0 and some constant *c*), and for an arbitrary positive constant k>0 and $g\left(t\right):={\left(t-1\right)}^{2}$ for t≥0, we get $D_{kg}\left(P∥Q\right)=k\;\chi^{2}\left(P∥Q\right)$, inequality (61) follows from (A117) and (A119). To that end, note that k=k(α) defined in (63) is monotonically increasing in α, and therefore $k\left(\alpha \right)\geq k\left(e^{-\frac{3}{2}}\right)>0.2075$ for all $\alpha \geq e^{-\frac{3}{2}}$. Due to the inequality (see, e.g., ([64], Theorem 5), followed by refined versions in ([62], Theorem 20) and ([65], Theorem 9))

(A121) $$\begin{array}{c} D\left(P∥Q\right)\leq \log\left(1+\chi^{2}\left(P∥Q\right)\right), \end{array}$$

the looser lower bound on $D_{f_{\alpha }}\left(P∥Q\right)$ in the right side of (62), expressed as a function of the relative entropy D(P∥Q), follows from (61). Hence, if *P* and *Q* are not identical, then (64) follows from (61) since $\chi^{2}\left(P∥Q\right)>0$ and $\lim_{\alpha \to \infty }k\left(\alpha \right)=\infty$.

We next prove Item (d). The Taylor series expansion of $f_{\alpha }\left(\cdot \right)$ implies that, for all t≥0,

(A122) $$\begin{array}{c} \;f_{\alpha }\left(t\right)=f_{\alpha }\left(1\right)+f_{\alpha }^{\prime }\left(1\right)\left(t-1\right)+\frac{1}{2}f_{\alpha }^{\prime \prime }\left(1\right){\left(t-1\right)}^{2}+\frac{1}{6}f_{\alpha }^{\left(3\right)}\left(1\right){\left(t-1\right)}^{3}+\frac{1}{24}f_{\alpha }^{\left(4\right)}\left(\xi \right){\left(t-1\right)}^{4} \end{array}$$

where ξ in the right side of (A122) is an intermediate value between 1 and *t*. Consequently, since $f_{\alpha }^{\left(4\right)}\left(\xi \right)=-\frac{2\log e}{{\left(\alpha +\xi \right)}^{2}}<0$ and $f_{\alpha }\left(1\right)=0$, it follows from (A122) that, for all t≥0,

(A123) $$\begin{array}{cc} f_{\alpha }\left(t\right) & \leq f_{\alpha }^{\prime }\left(1\right)\left(t-1\right)+\frac{1}{2}f_{\alpha }^{\prime \prime }\left(1\right){\left(t-1\right)}^{2}+\frac{1}{6}f_{\alpha }^{\left(3\right)}\left(1\right){\left(t-1\right)}^{3} \end{array}$$

(A124) $$\begin{array}{cc} \; & =f_{\alpha }^{\prime }\left(1\right)\left(t-1\right)+\frac{1}{2}f_{\alpha }^{\prime \prime }\left(1\right){\left(t-1\right)}^{2}+\frac{1}{6}f_{\alpha }^{\left(3\right)}\left(1\right)[t^{3}-3{\left(t-1\right)}^{2}-3\left(t-1\right)-1] \end{array}$$

(A125) $$\begin{array}{cc} \; & =\left[f_{\alpha }^{\prime }\left(1\right)-\frac{1}{2}f_{\alpha }^{\left(3\right)}\left(1\right)\right]\left(t-1\right)+\frac{1}{2}\left[f_{\alpha }^{\prime \prime }\left(1\right)-f_{\alpha }^{\left(3\right)}\left(1\right)\right]{\left(t-1\right)}^{2}+\frac{1}{6}f_{\alpha }^{\left(3\right)}\left(1\right)\;\left(t^{3}-1\right). \end{array}$$

Based on (A123)–(A125), it follows that

$$\begin{array}{cc} D_{f_{\alpha }}\left(P∥Q\right) & \leq \frac{1}{2}\left[f_{\alpha }^{\prime \prime }\left(1\right)-f_{\alpha }^{\left(3\right)}\left(1\right)\right]\chi^{2}\left(P∥Q\right)+\frac{1}{6}f_{\alpha }^{\left(3\right)}\left(1\right)\;\sum_{x\in X}\left\{Q\left(x\right)\left[{\left(\frac{P(x)}{Q(x)}\right)}^{3}-1\right]\right\} \end{array}$$

(A126) $$\begin{array}{cc} \; & =\frac{1}{2}\left[f_{\alpha }^{\prime \prime }\left(1\right)-f_{\alpha }^{\left(3\right)}\left(1\right)\right]\chi^{2}\left(P∥Q\right)+\frac{1}{6}f_{\alpha }^{\left(3\right)}\left(1\right)\left(-1+\sum_{x\in X}\frac{P^{3}\left(x\right)}{Q^{2}\left(x\right)}\right) \end{array}$$

(A127) $$\begin{array}{cc} \; & =\frac{1}{2}\left[f_{\alpha }^{\prime \prime }\left(1\right)-f_{\alpha }^{\left(3\right)}\left(1\right)\right]\chi^{2}\left(P∥Q\right)+\frac{1}{6}f_{\alpha }^{\left(3\right)}\left(1\right)\left[\exp\left(2D_{3}\left(P∥Q\right)\right)-1\right], \end{array}$$

where (A127) holds due to (A111) (with β=3). Substituting (A114) and (A115) into the right side of (A127) gives (65).

We next prove Item (e). Let *P* and *Q* be probability mass functions such that $D_{3}\left(P∥Q\right)<\infty$, and let ε>0 be arbitrarily small. Since the Rényi divergence $D_{\alpha }\left(P∥Q\right)$ is monotonically non-decreasing in α>0 (see ([66], Theorem 3)), it follows that $D_{2}\left(P∥Q\right)<\infty$, and therefore also

(A128) $$\begin{array}{c} \chi^{2}\left(P∥Q\right)=\exp\left(D_{2}\left(P∥Q\right)\right)-1<\infty . \end{array}$$

In view of (61), there exists $\alpha_{1}:=\alpha_{1}\left(P,Q,\varepsilon \right)$ such that for all $\alpha >\alpha_{1}$

(A129) $$\begin{array}{c} D_{f_{\alpha }}\left(P∥Q\right)>\left(\log\left(\alpha +1\right)+\frac{3}{2}\log e\right)\;\chi^{2}\left(P∥Q\right)-\varepsilon , \end{array}$$

and, from (65), there exists $\alpha_{2}:=\alpha_{2}\left(P,Q,\varepsilon \right)$ such that for all $\alpha >\alpha_{2}$

(A130) $$\begin{array}{c} D_{f_{\alpha }}\left(P∥Q\right)<\left(\log\left(\alpha +1\right)+\frac{3}{2}\log e\right)\;\chi^{2}\left(P∥Q\right)+\varepsilon . \end{array}$$

Letting $\alpha^{*}:=\max\left\{\alpha_{1},\alpha_{2}\right\}$ gives the result in (66) for all $\alpha >\alpha^{*}$.

Item (f) of Theorem 5 is a direct consequence of ([45], Lemma 4), which relies on ([67], Theorem 3). Let $g\left(t\right):={\left(t-1\right)}^{2}$ for t≥0 (hence, $D_{g}\left(\cdot ∥\cdot \right)$ is the $\chi^{2}$ divergence). If a sequence $\left\{P_{n}\right\}$ converges to a probability measure *Q* in the sense that the condition in (67) is satisfied, and $P_{n}\ll Q$ for all sufficiently large *n*, then ([45], Lemma 4) yields

(A131) $$\begin{array}{c} \lim_{n\to \infty }\frac{D_{f_{\alpha }}\left(P_{n}∥Q\right)}{\chi^{2}\left(P_{n}∥Q\right)}=\frac{1}{2}f_{\alpha }^{\prime \prime }\left(1\right), \end{array}$$

which gives (68) from (A114) and (A131).

We next prove Item (g). Inequality (69) is trivial. Inequality (70) is obtained as follows:

(A132) $$\begin{array}{cc} D_{f_{\alpha }}\left(P∥Q\right)-D_{f_{\beta }}\left(P∥Q\right) & =\int_{\beta }^{\alpha }\frac{\partial }{\partial u}\left\{D_{f_{u}}\left(P∥Q\right)\right\}\;du \end{array}$$

(A133) $$\begin{array}{cc} \; & =\int_{\beta }^{\alpha }2\left(u+1\right)\;D\left(\frac{uQ+P}{u+1}\;∥\;Q\right)\;du \end{array}$$

(A134) $$\begin{array}{cc} \; & \geq \int_{\beta }^{\alpha }2\left(u+1\right)\;du\cdot D\left(\frac{\alpha Q+P}{\alpha +1}\;∥\;Q\right) \end{array}$$

(A135) $$\begin{array}{cc} \; & =\left[{\left(\alpha +1\right)}^{2}-{\left(\beta +1\right)}^{2}\right]\;D\left(\frac{\alpha Q+P}{\alpha +1}\;∥\;Q\right) \end{array}$$

(A136) $$\begin{array}{cc} \; & =\left(\alpha -\beta \right)\left(\alpha +\beta +2\right)\;D\left(\frac{\alpha Q+P}{\alpha +1}\;∥\;Q\right) \end{array}$$

where (A133) follows from (56), and (A134) holds since the function I:[0,∞)→[0,∞) given by

(A137) $$\begin{array}{c} I\left(u\right):=D\left(\frac{uQ+P}{u+1}\;∥\;Q\right),\;u\geq 0 \end{array}$$

is monotonically decreasing in *u* (note that by increasing the value of the non-negative variable *u*, the probability mass function $\frac{uQ+P}{u+1}$ gets closer to *Q*). This gives (70).

For proving inequality (71), we obtain two upper bounds on $D_{f_{\alpha }}\left(P∥Q\right)-D_{f_{\beta }}\left(P∥Q\right)$ with $\alpha >\beta \geq e^{-\frac{3}{2}}$. For the derivation of the first bound, we rely on (A83). From (A84) and (A85),

(A138) $$\begin{array}{c} r_{\alpha }\left(t\right)=2t\log t-s_{\alpha }\left(t\right),\;t\geq 0 \end{array}$$

where $s_{\alpha }:\left(0,\infty \right)\to R$ is given by

(A139) $$\begin{array}{c} s_{\alpha }\left(t\right):=2t\log t-2\left(\alpha +t\right)\log\left(\alpha +t\right)+\left(1-t\right)\log e+2\left(\alpha +1\right)\log\left(\alpha +1\right),\;t\geq 0, \end{array}$$

with the convention that 0log0=0 (by a continuous extension of tlogt at t=0). Since $s_{\alpha }\left(1\right)=0$, and

(A140) $$\begin{array}{c} s_{\alpha }^{\prime \prime }\left(t\right)=\frac{2\alpha }{t(\alpha +t)}>0,\;\forall \;t>0, \end{array}$$

which implies that $s_{\alpha }\left(\cdot \right)$ is convex on (0,∞), we get

(A141) $$\begin{array}{cc} \frac{\partial }{\partial \alpha }\left\{D_{f_{\alpha }}\left(P∥Q\right)\right\}\; & =D_{r_{\alpha }}\left(P∥Q\right) \end{array}$$

(A142) $$\begin{array}{cc} \; & =2D\left(P∥Q\right)-D_{s_{\alpha }}\left(P∥Q\right) \end{array}$$

(A143) $$\begin{array}{cc} \; & \leq 2D(P∥Q) \end{array}$$

where (A141) holds due to (A83) (recall the convexity of $r_{\alpha }:\left(0,\infty \right)\to R$ with $r_{\alpha }\left(1\right)=0$); (A142) holds due to (A138) and since r(t):=tlogt for t>0 implies that $D_{r}\left(P∥Q\right)=D\left(P∥Q\right)$; finally, (A143) follows from the non-negativity of the *f*-divergence $D_{s_{\alpha }}\left(\cdot ∥\cdot \right)$. Consequently, integration over the interval [β,α] (α>β) on the left side of (A141) and the right side of (A143) gives

(A144) $$\begin{array}{c} D_{f_{\alpha }}\left(P∥Q\right)-D_{f_{\beta }}\left(P∥Q\right)\leq 2\left(\alpha -\beta \right)\;D\left(P∥Q\right). \end{array}$$

Note that the same reasoning of (A132)–(A136) also implies that

(A145) $$\begin{array}{cc} D_{f_{\alpha }}\left(P∥Q\right)-D_{f_{\beta }}\left(P∥Q\right)\; & \leq \left(\alpha -\beta \right)\left(\alpha +\beta +2\right)\;D\left(\frac{\beta Q+P}{\beta +1}\;∥\;Q\right), \end{array}$$

which gives a second upper bound on the left side of (A145). Taking the minimal value among the two upper bounds in the right sides of (A144) and (A145) gives (71) (see Remark A4).

We finally prove Item (h). From (55) and (A81), the function $f_{\alpha }:\left[0,\infty \right)\to R$ is convex for $\alpha \geq e^{-\frac{3}{2}}$ with $f_{\alpha }\left(1\right)=0$, $f_{\alpha }\left(0\right)=\alpha^{2}\log\alpha -{\left(\alpha +1\right)}^{2}\log\left(\alpha +1\right)\in R$, and it is also differentiable at 1. It is left to prove that the function $g_{\alpha }:\left(0,\infty \right)\to R$, defined as $g_{\alpha }\left(t\right):=\frac{f_{\alpha }\left(t\right)-f_{\alpha }\left(0\right)}{t}$ for t>0, is convex. From (55), the function $g_{\alpha }$ is given explicitly by

(A146) $$\begin{array}{c} g_{\alpha }\left(t\right)=\frac{{\left(\alpha +t\right)}^{2}\log\left(\alpha +t\right)-\alpha^{2}\log\alpha }{t},\;t>0, \end{array}$$

and its second derivative is given by

(A147) $$\begin{array}{c} g_{\alpha }^{\prime \prime }\left(t\right)=\frac{w_{\alpha }\left(t\right)}{t^{3}},\;t>0, \end{array}$$

with

(A148) $$\begin{array}{c} w_{\alpha }\left(t\right):=2\alpha^{2}\log\left(1+\frac{t}{\alpha }\right)+t\left(t-2\alpha \right)\log e,\;t\geq 0. \end{array}$$

Since $w_{\alpha }\left(0\right)=0$, and

(A149) $$\begin{array}{c} w_{\alpha }^{\prime }\left(t\right)=\frac{2t^{2}\log e}{\alpha +t}>0,\;\forall \;t>0, \end{array}$$

it follows that $w_{\alpha }\left(t\right)>0$ for all t>0; hence, from (A147), $g_{\alpha }^{\prime \prime }\left(t\right)>0$ for t∈(0,∞), which yields the convexity of the function $g_{\alpha }\left(\cdot \right)$ on (0,∞) for all α≥0. This shows that, for every $\alpha \geq e^{-\frac{3}{2}}$, the function $f_{\alpha }:\left[0,\infty \right)\to R$ satisfies all the required conditions in Theorems 3 and 4. We proceed to calculate the function $\kappa_{\alpha }:\left[0,1\right)\times \left(1,\infty \right)\to R$ in (51), which corresponds to $f:=f_{\alpha }$, i.e., (see (72)),

(A150) $$\begin{array}{cc} \; & \kappa_{\alpha }\left(\xi_{1},\xi_{2}\right)=\underset{t\in \left(\xi_{1},1\right)\cup \left(1,\xi_{2}\right)}{\sup}z_{\alpha }\left(t\right), \end{array}$$

with

(A151) $$z_{\alpha }\left(t\right):=\{\begin{array}{cc} \frac{f_{\alpha }\left(t\right)+f_{\alpha }^{\prime }\left(1\right)\;\left(1-t\right)}{{\left(t-1\right)}^{2}}, & t\in [0,1)\cup (1,\infty ),\\ \frac{3}{2}\;\log e+\log\left(\alpha +1\right), & t=1, \end{array}$$

where the definition of $z_{\alpha }\left(1\right)$ is obtained by continuous extension of the function $z_{\alpha }\left(\cdot \right)$ at t=1 (recall that the function $f_{\alpha }\left(\cdot \right)$ is given in (55)). Differentiation shows that

(A152) $$\begin{array}{c} \frac{\partial \;z_{\alpha }\left(t\right)}{\partial t}=\frac{v_{\alpha }\left(t\right)}{{\left(t-1\right)}^{4}},\;t\in \left[0,1\right)\cup \left(1,\infty \right), \end{array}$$

where, for t≥0,

(A153) $$\begin{array}{cc} \; & v_{\alpha }\left(t\right):=\left(2\alpha +t+1\right){\left(t-1\right)}^{2}\log e-2\left(\alpha +1\right)\left(\alpha +t\right)\left(t-1\right)\log\frac{\alpha +t}{\alpha +1}, \end{array}$$

and

(A154) $$\begin{array}{cc} \; & v_{\alpha }^{\prime }\left(t\right)={\left(t-1\right)}^{2}\log e+2\left(\alpha +t\right)\left(t-1\right)\log e-2\left(\alpha +1\right)\left(2t+\alpha -1\right)\log\frac{\alpha +t}{\alpha +1}, \end{array}$$

(A155) $$\begin{array}{cc} \; & v_{\alpha }^{\prime \prime }\left(t\right)=6\left(t-1\right)\log e+\frac{2{\left(\alpha +1\right)}^{2}\log e}{\alpha +t}-4\left(\alpha +1\right)\log\frac{\alpha +t}{\alpha +1}, \end{array}$$

(A156) $$\begin{array}{cc} \; & v_{\alpha }^{\left(3\right)}\left(t\right)=\frac{2(t-1)(3t+4\alpha +1)}{{\left(\alpha +t\right)}^{2}}. \end{array}$$

From (A156), it follows that $v_{\alpha }^{\left(3\right)}\left(t\right)<0$ if t∈[0,1), $v_{\alpha }^{\left(3\right)}\left(1\right)=0$, and $v_{\alpha }^{\left(3\right)}\left(t\right)>0$ if t∈(1,∞). Since $v_{\alpha }^{\prime \prime }\left(\cdot \right)$ is therefore monotonically decreasing on [0,1] and it is monotonically increasing on [1,∞), (A155) implies that

(A157) $$\begin{array}{c} v_{\alpha }^{\prime \prime }\left(t\right)\geq v_{\alpha }^{\prime \prime }\left(1\right)=2\left(\alpha +1\right)\log e>0,\;\forall \;t\geq 0. \end{array}$$

Since $v_{\alpha }^{\prime }\left(1\right)=0$ (see (A154)), and $v_{\alpha }^{\prime }\left(\cdot \right)$ is monotonically increasing on [0,∞), it follows that $v_{\alpha }^{\prime }\left(t\right)<0$ for all t∈[0,1) and $v_{\alpha }^{\prime }\left(t\right)>0$ for all t>1. This implies that $v_{\alpha }\left(t\right)\geq v_{\alpha }\left(1\right)=0$ for all t≥0 (see (A153)); hence, from (A152), the function $z_{\alpha }\left(\cdot \right)$ is monotonically increasing on [0,∞), and it is continuous over this interval (see (A151)). It therefore follows from (A150) that

(A158) $$\begin{array}{c} \kappa_{\alpha }\left(\xi_{1},\xi_{2}\right)=z_{\alpha }\left(\xi_{2}\right), \end{array}$$

for every $\xi_{1}\in \left[0,1\right)$ and $\xi_{2}\in \left(1,\infty \right)$ (independently of $\xi_{1}$), which proves (73).

**Remark** **A4.** *None of the upper bounds in the right sides of* (A144) *and* (A145) *supersedes the other. For example, if P and Q correspond to Bernoulli(p) and Bernoulli(q), respectively, and $\left(\alpha ,\beta ,p,q\right)=\left(2,1,\frac{1}{5},\frac{2}{5}\right)$, then the right sides of* (A144) *and* (A145) *are, respectively, equal to 0.264loge and 0.156loge. If on the other hand $\left(\alpha ,\beta ,p,q\right)=\left(10,1,\frac{1}{5},\frac{2}{5}\right)$, then the right sides of* (A144) *and* (A145) *are, respectively, equal to 2.377loge and 3.646loge.*

## Appendix E. Proof of Theorem 6

By assumption, P≺Q where the probability mass functions *P* and *Q* are defined on the set A:={1,…,n}. The majorization relation P≺Q is equivalent to the existence of a doubly-stochastic transformation $W_{Y|X}:A\to A$ such that (see Proposition 4)

(A159) $$\begin{array}{c} Q\to W_{Y|X}\to P. \end{array}$$

(See, e.g., ([32], Theorem 2.1.10) or ([30], Theorem 2.B.2) or ([31], pp. 195–204)). Define

(A160) $$\begin{array}{c} X=Y:=A,\;P_{X}:=Q,\;Q_{X}:=U_{n}. \end{array}$$

The probability mass functions given by

(A161) $$\begin{array}{c} P_{Y}:=P,\;Q_{Y}:=U_{n} \end{array}$$

satisfy, respectively, (20) and (21). The first one is obvious from (A159)–(A161); equality (21) holds due to the fact that $W_{Y|X}:A\to A$ is a doubly stochastic transformation, which implies that for all y∈A

(A162) $$\begin{array}{cc} \sum_{x\in A}Q_{X}\left(x\right)P_{Y|X}\left(y|x\right)\; & =\frac{1}{n}\sum_{x\in A}P_{Y|X}\left(y|x\right) \end{array}$$

(A163) $$\begin{array}{cc} \; & =\frac{1}{n}=Q_{Y}\left(y\right). \end{array}$$

Since (by assumption) $P_{X}$ and $Q_{X}$ are supported on A, relations (20) and (21) hold in the setting of (A159)–(A161), and f:(0,∞)→R is (by assumption) convex and twice differentiable, it is possible to apply the bounds in Theorem 1 (b) and (d). To that end, from (18), (19), (A160) and (A161),

(A164) $$\begin{array}{cc} \; & \xi_{1}=\min_{x\in A}\frac{Q(x)}{\frac{1}{n}}=nq_{\min}, \end{array}$$

(A165) $$\begin{array}{cc} \; & \xi_{2}=\max_{x\in A}\frac{Q(x)}{\frac{1}{n}}=nq_{\max}, \end{array}$$

which, from (24), (25), (32), (A160), (A161) and (A164), give that

$$\begin{array}{cc} \; & e_{f}\left(nq_{\min},nq_{\max}\right)\left[\chi^{2}\left(Q∥U_{n}\right)-\chi^{2}\left(P∥U_{n}\right)\right] \end{array}$$

(A166) $$\begin{array}{cc} \; & \geq D_{f}\left(Q∥U_{n}\right)-D_{f}\left(P∥U_{n}\right) \end{array}$$

(A167) $$\begin{array}{cc} \; & \geq c_{f}\left(nq_{\min},nq_{\max}\right)\left[\chi^{2}\left(Q∥U_{n}\right)-\chi^{2}\left(P∥U_{n}\right)\right] \end{array}$$

(A168) $$\begin{array}{cc} \; & \geq 0. \end{array}$$

The difference of the $\chi^{2}$ divergences in the left side of (A166) and the right side of (A167) satisfies

(A169) $$\begin{array}{cc} \chi^{2}\left(Q∥U_{n}\right)-\chi^{2}\left(P∥U_{n}\right) & =\sum_{x\in A}\frac{Q^{2}\left(x\right)}{\frac{1}{n}}-\sum_{x\in A}\frac{P^{2}\left(x\right)}{\frac{1}{n}}\\ & =n\left({∥Q∥}_{2}^{2}-{∥P∥}_{2}^{2}\right), \end{array}$$

and the substitution of (A169) into the bounds in (A166) and (A167) give the result in (74) and (75).

Let $f\left(t\right)={\left(t-1\right)}^{2}$ for t>0, which yields from (26) and (31) that $c_{f}\left(\cdot ,\cdot \right)=e_{f}\left(\cdot ,\cdot \right)=1$. Since $D_{f}\left(\cdot ∥\cdot \right)=\chi^{2}\left(\cdot ∥\cdot \right)$, it follows from (A169) that the upper and lower bounds in the left side of (74) and the right side of (75), respectively, coincide for the $\chi^{2}$-divergence; this therefore yields the tightness of these bounds in this special case.

We next prove (76). The following lower bound on the second-order Rényi entropy (a.k.a. the collision entropy) holds (see ([34], (25)–(27))):

(A170) $$\begin{array}{c} H_{2}\left(Q\right):=-\log\left({∥Q∥}_{2}^{2}\right)\geq \log\frac{4n\rho }{{\left(1+\rho \right)}^{2}}, \end{array}$$

where $\frac{q_{\max}}{q_{\min}}\leq \rho$. This gives

(A171) $$\begin{array}{c} {∥Q∥}_{2}^{2}=\exp\left(-H_{2}\left(Q\right)\right)\leq \frac{{\left(1+\rho \right)}^{2}}{4n\rho }. \end{array}$$

By Cauchy-Schwartz inequality ${∥P∥}_{2}^{2}\geq \frac{1}{n}$ which, together with (A171), give

(A172) $$\begin{array}{c} {∥Q∥}_{2}^{2}-{∥P∥}_{2}^{2}\leq \frac{{\left(\rho -1\right)}^{2}}{4n\rho }. \end{array}$$

In view of the Schur-concavity of the Rényi entropy (see ([30], Theorem 13.F.3.a.)), the assumption P≺Q implies that

(A173) $$\begin{array}{c} H_{2}\left(P\right)\geq H_{2}\left(Q\right), \end{array}$$

and an exponentiation of both sides of (A173) (see the left-side equality in (A170)) gives

(A174) $$\begin{array}{c} {∥Q∥}_{2}^{2}\geq {∥P∥}_{2}^{2}. \end{array}$$

Combining (A172) and (A173) gives (76).

## Appendix F. Proof of Theorem 7

We prove Item (a), showing that the set $P_{n}\left(\rho \right)$ (with ρ≥1) is non-empty, convex and compact. Note that $P_{n}\left(1\right)=\left\{U_{n}\right\}$ is a singleton, so the claim is trivial for ρ=1.

Let ρ>1. The non-emptiness of $P_{n}\left(\rho \right)$ is trivial since $U_{n}\in P_{n}\left(\rho \right)$. To prove the convexity of $P_{n}\left(\rho \right)$, let $P_{1},P_{2}\in P_{n}\left(\rho \right)$, and let $p_{\max}^{\left(1\right)},\;p_{\max}^{\left(2\right)},\;p_{\min}^{\left(1\right)}$ and $p_{\min}^{\left(2\right)}$ be the (positive) maximal and minimal probability masses of $P_{1}$ and $P_{2}$, respectively. Then, $\frac{p_{\max}^{\left(1\right)}}{p_{\min}^{\left(1\right)}}\leq \rho$ and $\frac{p_{\max}^{\left(2\right)}}{p_{\min}^{\left(2\right)}}\leq \rho$ yield

(A175) $$\begin{array}{c} \frac{\lambda p_{\max}^{\left(1\right)}+\left(1-\lambda \right)p_{\max}^{\left(2\right)}}{\lambda p_{\min}^{\left(1\right)}+\left(1-\lambda \right)p_{\min}^{\left(2\right)}}\leq \rho ,\;\forall \;\lambda \in \left[0,1\right]. \end{array}$$

For every λ∈[0,1],

(A176) $$\begin{array}{cc} \; & \min_{1\leq i\leq n}\left\{\lambda P_{1}\left(i\right)+\left(1-\lambda \right)P_{2}\left(i\right)\right\}\geq \lambda \;p_{\min}^{\left(1\right)}+\left(1-\lambda \right)\;p_{\min}^{\left(2\right)}, \end{array}$$

(A177) $$\begin{array}{cc} \; & \max_{1\leq i\leq n}\left\{\lambda P_{1}\left(i\right)+\left(1-\lambda \right)P_{2}\left(i\right)\right\}\leq \lambda \;p_{\max}^{\left(1\right)}+\left(1-\lambda \right)\;p_{\max}^{\left(2\right)}. \end{array}$$

Combining (A175)–(A177) implies that

(A178) $$\begin{array}{c} \frac{\max_{1\leq i\leq n}\left\{\lambda P_{1}\left(i\right)+\left(1-\lambda \right)P_{2}\left(i\right)\right\}}{\min_{1\leq i\leq n}\left\{\lambda P_{1}\left(i\right)+\left(1-\lambda \right)P_{2}\left(i\right)\right\}}\leq \rho , \end{array}$$

so $\lambda P_{1}+\left(1-\lambda \right)P_{2}\in P_{n}\left(\rho \right)$ for all λ∈[0,1]. This proves the convexity of $P_{n}\left(\rho \right)$.

The set of probability mass functions $P_{n}\left(\rho \right)$ is clearly bounded; for showing its compactness, it is left to show that $P_{n}\left(\rho \right)$ is closed. Let ρ>1, and let ${\left\{P^{\left(m\right)}\right\}}_{m=1}^{\infty }$ be a sequence of probability mass functions in $P_{n}\left(\rho \right)$ which pointwise converges to *P* over the finite set $A_{n}$. It is required to show that $P\in P_{n}\left(\rho \right)\subseteq P_{n}$. As a limit of probability mass functions, $P\in P_{n}$, and since by assumption $P^{\left(m\right)}\in P_{n}\left(\rho \right)$ for all m∈N, it follows that

$$\left(n-1\right)\rho p_{\min}^{\left(m\right)}+p_{\min}^{\left(m\right)}\geq \left(n-1\right)p_{\max}^{\left(m\right)}+p_{\min}^{\left(m\right)}\geq 1,$$

which yields $p_{\min}^{\left(m\right)}\geq \frac{1}{(n-1)\rho +1}$ for all *m*. Since $p_{\max}^{\left(m\right)}\leq \rho p_{\min}^{\left(m\right)}$ for every *m*, it follows that also for the limiting probability mass function *P* we have $p_{\min}\geq \frac{1}{(n-1)\rho +1}>0$, and $p_{\max}\leq \rho p_{\min}$. This proves that $P\in P_{n}\left(\rho \right)$, and therefore $P_{n}\left(\rho \right)$ is a closed set.

An alternative proof for Item (a) relies on the observation that, for ρ≥1,

(A179) $$\begin{array}{c} P_{n}\left(\rho \right)=P_{n}⋂\left\{\underset{i\neq j}{⋂}\left\{P:\;P(i)-\rho P(j)\leq 0\right\}\right\}, \end{array}$$

which yields the convexity and compactness of the set $P_{n}\left(\rho \right)$ for all ρ≥1.

The result in Item (b) holds in view of Item (a), and due to the convexity and continuity of $D_{f}\left(P∥Q\right)$ in $\left(P,Q\right)\in P_{n}\left(\rho \right)\times P_{n}\left(\rho \right)$ (where $p_{\min},\;q_{\min}\geq \frac{1}{(n-1)\rho +1}>0$). This implication is justified by the statement that a convex and continuous function over a non-empty convex and compact set attains its supremum over this set (see, e.g., ([68], Theorem 7.42) or ([59], Theorem 10.1 and Corollary 32.3.2)).

We next prove Item (c). If $Q\in P_{n}\left(\rho \right)$, then $\frac{1}{1+(n-1)\rho }\leq q_{\min}\leq \frac{1}{n}$ where the lower bound on $q_{\min}$ is attained when *Q* is the probability mass function with n−1 masses equal to $\rho q_{\min}$ and a single smaller mass equal to $q_{\min}$, and the upper bound is attained when *Q* is the equiprobable distribution. For an arbitrary $Q\in P_{n}\left(\rho \right)$, let $q_{\min}:=\beta$ where β can get any value in the interval $\Gamma_{n}\left(\rho \right)$ defined in (79). By ([34], Lemma 1), $Q≺Q_{\beta }$ and $Q_{\beta }\in P_{n}\left(\rho \right)$ where $Q_{\beta }$ is given in (80). The Schur-convexity of $D_{f}\left(\cdot ∥U_{n}\right)$ (see ([38], Lemma 1)) and the identity $D_{f}\left(U_{n}∥\cdot \right)=D_{f^{*}}\left(\cdot ∥U_{n}\right)$ give that

(A180) $$\begin{array}{c} D_{f}\left(Q∥U_{n}\right)\leq D_{f}\left(Q_{\beta }∥U_{n}\right),\;D_{f}\left(U_{n}∥Q\right)\leq D_{f}\left(U_{n}∥Q_{\beta }\right) \end{array}$$

for all $Q\in P_{n}\left(\rho \right)$ with $q_{\min}=\beta \in \Gamma_{n}\left(\rho \right)$; equalities hold in (A180) if $Q=Q_{\beta }\in P_{n}\left(\rho \right)$. The maximization of $D_{f}\left(Q∥U_{n}\right)$ and $D_{f}\left(U_{n}∥Q\right)$ over all probability mass functions $Q\in P_{n}\left(\rho \right)$ can be therefore simplified to the maximization of $D_{f}\left(Q_{\beta }∥U_{n}\right)$ and $D_{f}\left(U_{n}∥Q_{\beta }\right)$, respectively, over the parameter β which lies in the interval $\Gamma_{n}\left(\rho \right)$ in (79). This proves (82) and (83).

We next prove Item (e), and then prove Item (d). In view of Item (c), the maximum of $D_{f}\left(Q∥U_{n}\right)$ over all the probability mass functions $Q\in P_{n}\left(\rho \right)$ is attained by $Q=Q_{\beta }$ with $\beta \in \Gamma_{n}\left(\rho \right)$ (see (79)–(81)). From (80), $Q_{\beta }$ can be expressed as the *n*-length probability vector

(A181) $$\begin{array}{c} Q_{\beta }=\left(\underset{i_{\beta }}{\underset{︸}{\rho \beta ,\ldots ,\rho \beta }},\;1-\left(n+i_{\beta }\rho -i_{\beta }-1\right)\beta ,\;\underset{n-i_{\beta }-1}{\underset{︸}{\beta ,\ldots ,\beta }}\right). \end{array}$$

The influence of the $\left(i_{\beta }+1\right)$-th entry of the probability vector in (A181) on $D_{f}\left(Q_{\beta }∥U_{n}\right)$ tends to zero as we let n→∞. This holds since the entries of the vector in (A181) are written in decreasing order, which implies that for all $\beta \in \Gamma_{n}\left(\rho \right)$ (with ρ≥1)

(A182) $$\begin{array}{c} n\left[1-(n+i_{\beta }\rho -i_{\beta }-1)\right]\in \left[n\beta ,n\rho \beta \right]\subseteq \left[\frac{n}{(n-1)\rho +1},\rho \right]\subseteq \left[\frac{1}{\rho },\rho \right]; \end{array}$$

from (A182) and the convexity of *f* on (0,∞) (so, *f* attains its finite maximum on every closed sub-interval of (0,∞)), it follows that

(A183) $$\begin{array}{c} \left|\left[1-(n+i_{\beta }\rho -i_{\beta }-1)\beta \right]\;f\left(n\left[1-(n+i_{\beta }\rho -i_{\beta }-1)\right]\right)\right|\\ \leq \left|\left[1-(n+i_{\beta }\rho -i_{\beta }-1)\beta \right]\right|\;\max_{u\in \left[\frac{1}{\rho },\rho \right]}\;\left|f(u)\right|\\ \leq \frac{\rho }{n}\;\max_{u\in \left[\frac{1}{\rho },\rho \right]}\;\left|f(u)\right|\underset{n\to \infty }{⟶}0. \end{array}$$

In view of (A181) and (A183), by letting n→∞, the maximization of $D_{f}\left(Q_{\beta }∥U_{n}\right)$ over $\beta \in \Gamma_{n}\left(\rho \right)$ can be replaced by a maximization of $D_{f}\left({\tilde{Q}}_{m}∥U_{n}\right)$ where

(A184) $$\begin{array}{c} {\tilde{Q}}_{m}:=\left(\;\underset{m}{\underset{︸}{\rho \beta ,\ldots ,\rho \beta }},\underset{n-m}{\underset{︸}{\beta ,\ldots ,\beta }}\;\right)\in P_{n}\left(\rho \right) \end{array}$$

with the free parameter m∈{0,…,n}, and with $\beta :=\frac{1}{n+(\rho -1)m}$ (the value of β is determined so that the total mass of ${\tilde{Q}}_{m}$ is 1). Hence, we get

(A185) $$\begin{array}{c} \lim_{n\to \infty }\max_{\beta \in \Gamma_{n}\left(\rho \right)}D_{f}\left(Q_{\beta }∥U_{n}\right)=\lim_{n\to \infty }\max_{m\in \{0,\ldots ,n\}}D_{f}\left({\tilde{Q}}_{m}∥U_{n}\right). \end{array}$$

The *f*-divergence in the right side of (A185) satisfies

(A186) $$\begin{array}{cc} D_{f}\left({\tilde{Q}}_{m}∥U_{n}\right)\; & =\frac{1}{n}\sum_{i=1}^{n}f\left(n\;{\tilde{Q}}_{m}\left(i\right)\right) \end{array}$$

(A187) $$\begin{array}{cc} \; & =\frac{m}{n}\;f\left(\frac{\rho n}{n+(\rho -1)m}\right)+\left(1-\frac{m}{n}\right)\;f\left(\frac{n}{n+(\rho -1)m}\right) \end{array}$$

(A188) $$\begin{array}{cc} \; & =g_{f}^{\left(\rho \right)}\left(\frac{m}{n}\right), \end{array}$$

where (A188) holds by the definition of the function $g_{f}^{\left(\rho \right)}\left(\cdot \right)$ in (84). It therefore follows that

$$\begin{array}{cc} \; & \lim_{n\to \infty }u_{f}\left(n,\rho \right) \end{array}$$

(A189) $$\begin{array}{cc} \; & =\lim_{n\to \infty }\max_{m\in \{0,\ldots ,n\}}g_{f}^{\left(\rho \right)}\left(\frac{m}{n}\right) \end{array}$$

(A190) $$\begin{array}{cc} \; & =\max_{x\in [0,1]}g_{f}^{\left(\rho \right)}\left(x\right) \end{array}$$

where (A189) holds by combining (82) and (A185)–(A188); (A190) holds by the continuity of the function $g_{f}^{\left(\rho \right)}\left(\cdot \right)$ on [0,1], which follows from (84) and the continuity of the convex function *f* on $\left[\frac{1}{\rho },\rho \right]$ for ρ≥1 (recall that a convex function is continuous on every closed sub-interval of its domain of region, and by assumption *f* is convex on (0,∞)). This proves (87), by the definition of $g_{f}^{\left(\rho \right)}\left(\cdot \right)$ in (84).

Equality (88) follows from (87) by replacing $g_{f}^{\left(\rho \right)}\left(\cdot \right)$ with $g_{f^{*}}^{\left(\rho \right)}\left(\cdot \right)$, with $f^{*}:\left(0,\infty \right)\to R$ as given in (29); this replacement is justified by the equality $D_{f}\left(U_{n}∥Q\right)=D_{f^{*}}\left(Q∥U_{n}\right)$.

Once Item (e) is proved, we return to prove Item (d). To that end, it is first shown that

(A191) $$\begin{array}{cc} \; & u_{f}\left(n,\rho \right)\leq u_{f}\left(2n,\rho \right), \end{array}$$

(A192) $$\begin{array}{cc} \; & v_{f}\left(n,\rho \right)\leq v_{f}\left(2n,\rho \right), \end{array}$$

for all ρ≥1 and integers n≥2, with the functions $u_{f}$ and $v_{f}$, respectively, defined in (77) and (78). Since $D_{f}\left(P∥Q\right)=D_{f^{*}}\left(Q∥P\right)$ for all $P,Q\in P_{n}$, (77) and (78) give that

(A193) $$\begin{array}{c} v_{f}\left(n,\rho \right)=u_{f^{*}}\left(n,\rho \right), \end{array}$$

so the monotonicity property in (A192) follows from (A191) by replacing *f* with $f^{*}$. To prove (A191), let $Q^{*}\in P_{n}\left(\rho \right)$ be a probability mass function which attains the maximum at the right side of (77), and let $P^{*}$ be the probability mass function supported on $A_{2n}=\left\{1,\ldots ,2n\right\}$, and defined as follows:

(A194) $$P^{*}\left(i\right)=\{\begin{array}{cc} \frac{1}{2}Q^{*}\left(i\right), & if\;i\in \{1,\ldots ,n\},\\ \frac{1}{2}Q^{*}\left(i-n\right), & if\;i\in \{n+1,\ldots ,2n\}. \end{array}$$

Since by assumption $Q^{*}\in P_{n}\left(\rho \right)$, (A194) implies that $P^{*}\in P_{2n}\left(\rho \right)$. It therefore follows that

(A195) $$\begin{array}{cc} u_{f}\left(2n,\rho \right) & =\max_{Q\in P_{2n}\left(\rho \right)}D_{f}\left(Q∥U_{2n}\right) \end{array}$$

(A196) $$\begin{array}{cc} \; & \geq D_{f}\left(P^{*}∥U_{2n}\right) \end{array}$$

(A197) $$\begin{array}{cc} \; & =\frac{1}{2n}\left[\;\sum_{i=1}^{n}f\left(2nP^{*}\left(i\right)\right)+\sum_{i=n+1}^{2n}f\left(2nP^{*}\left(i\right)\right)\right] \end{array}$$

(A198) $$\begin{array}{cc} \; & =\frac{1}{n}\sum_{i=1}^{n}f\left(nQ^{*}\left(i\right)\right) \end{array}$$

(A199) $$\begin{array}{cc} \; & =D_{f}\left(Q^{*}∥U_{n}\right) \end{array}$$

(A200) $$\begin{array}{cc} \; & =\max_{Q\in P_{n}\left(\rho \right)}D_{f}\left(Q∥U_{2n}\right) \end{array}$$

(A201) $$\begin{array}{cc} \; & =u_{f}\left(n,\rho \right) \end{array}$$

where (A195) and (A201) hold due to (77); (A196) holds since $P^{*}\in P_{2n}\left(\rho \right)$; finally, (A198) holds due to (A194), which implies that the two sums in the right side of (A197) are identical, and they equal to the sum in the right side of (A198). This gives (A191), and likewise also (A192) (see (A193)).

(A202) $$\begin{array}{cc} u_{f}\left(n,\rho \right) & \leq \lim_{k\to \infty }u_{f}\left(2^{k}n,\rho \right) \end{array}$$

(A203) $$\begin{array}{cc} \; & =\lim_{n^{\prime }\to \infty }u_{f}\left(n^{\prime },\rho \right) \end{array}$$

(A204) $$\begin{array}{cc} \; & =\max_{x\in [0,1]}g_{f}^{\left(\rho \right)}\left(x\right) \end{array}$$

where (A202) holds since, due to (A191), the sequence ${\left\{u_{f}\left(2^{k}n,\rho \right)\right\}}_{k=0}^{\infty }$ is monotonically increasing, which implies that the first term of this sequence is less than or equal to its limit. Equality (A203) holds since the limit in its right side exists (in view of the above proof of (87)), so its limit coincides with the limit of every subsequence; (A204) holds due to (A189) and (A190). A replacement of *f* with $f^{*}$ gives, from (A193), that

(A205) $$\begin{array}{c} v_{f}\left(n,\rho \right)\leq \max_{x\in [0,1]}g_{f^{*}}^{\left(\rho \right)}\left(x\right). \end{array}$$

Combining (A202)–(A205) gives the right-side inequalities in (85) and (86). The left-side inequality in (85) follows by combining (77), (A184) and (A186)–(A188), which gives

(A206) $$\begin{array}{cc} u_{f}\left(n,\rho \right) & =\max_{Q\in P_{n}\left(\rho \right)}D_{f}\left(Q∥U_{n}\right) \end{array}$$

(A207) $$\begin{array}{cc} \; & \geq \max_{m\in \{0,\ldots ,n\}}D_{f}\left({\tilde{Q}}_{m}∥U_{n}\right) \end{array}$$

(A208) $$\begin{array}{cc} \; & =\max_{m\in \{0,\ldots ,n\}}g_{f}^{\left(\rho \right)}\left(\frac{m}{n}\right). \end{array}$$

Likewise, in view of (A193), the left-side inequality in (86) follows from the left-side inequality in (85) by replacing *f* with $f^{*}$.

We next prove Item (f), providing an upper bound on the convergence rate of the limit in (87); an analogous result can be obtained for the convergence rate to the limit in (88) by replacing *f* with $f^{*}$ in (29). To prove (89), in view of Items (d) and (e), we get that for every integer n≥2

(A209) $$\begin{array}{cc} 0 & \leq \lim_{n^{\prime }\to \infty }\left\{u_{f}\left(n^{\prime },\rho \right)\right\}-u_{f}\left(n,\rho \right) \end{array}$$

(A210) $$\begin{array}{cc} \; & \leq \max_{x\in [0,1]}g_{f}^{\left(\rho \right)}\left(x\right)-\max_{m\in \{0,\ldots ,n\}}g_{f}^{\left(\rho \right)}\left(\frac{m}{n}\right) \end{array}$$

(A211) $$\begin{array}{cc} \; & =\max_{x\in [0,1]}g_{f}^{\left(\rho \right)}\left(x\right)-\max_{m\in \{0,\ldots ,n-1\}}g_{f}^{\left(\rho \right)}\left(\frac{m}{n}\right) \end{array}$$

(A212) $$\begin{array}{cc} \; & =\max_{m\in \{0,\ldots ,n-1\}}\left\{\max_{x\in \left[\frac{m}{n},\frac{m+1}{n}\right]}g_{f}^{\left(\rho \right)}\left(x\right)\right\}-\max_{m\in \{0,\ldots ,n-1\}}g_{f}^{\left(\rho \right)}\left(\frac{m}{n}\right) \end{array}$$

(A213) $$\begin{array}{cc} \; & \leq \max_{m\in \{0,\ldots ,n-1\}}\left\{\max_{x\in \left[\frac{m}{n},\frac{m+1}{n}\right]}\left\{g_{f}^{\left(\rho \right)}\left(x\right)-g_{f}^{\left(\rho \right)}\left(\frac{m}{n}\right)\right\}\right\} \end{array}$$

where (A209) holds due to monotonicity property in (A191), and also due to the existence of the limit of ${\left\{u_{f}\left(n^{\prime },\rho \right)\right\}}_{n^{\prime }\in N}$; (A210) holds due to (85); (A211) holds since the function $g_{f}^{\left(\rho \right)}:\left[0,1\right]\to R$ (as it is defined in (84)) satisfies $g_{f}^{\left(\rho \right)}\left(1\right)=g_{f}^{\left(\rho \right)}\left(0\right)=0$ (recall that by assumption f(1)=0); (A212) holds since $\left[0,1\right]=\overset{n-1}{\underset{m=1}{⋃}}\left[\frac{m}{n},\frac{m+1}{n}\right]$, so the maximization of $g_{f}^{\left(\rho \right)}\left(\cdot \right)$ over the interval [0,1] is the maximum over the maximal values over the sub-intervals $\left[\frac{m}{n},\frac{m+1}{n}\right]$ for m∈{0,…,n−1}; finally, (A213) holds since the maximum of a sum of functions is less than or equal to the sum of the maxima of these functions. If the function $g_{f}^{\left(\rho \right)}:\left[0,1\right]\to R$ is differentiable on (0,1), and its derivative is upper bounded by $K_{f}\left(\rho \right)\geq 0$, then by the mean value theorem of Lagrange, for every m∈{0,…,n−1},

(A214) $$\begin{array}{c} g_{f}^{\left(\rho \right)}\left(x\right)-g_{f}^{\left(\rho \right)}\left(\frac{m}{n}\right)\leq \frac{K_{f}\left(\rho \right)}{n},\;\forall \;x\in \left[\frac{m}{n},\frac{m+1}{n}\right]. \end{array}$$

Combining (A209)–(A214) gives (89).

We next prove Item (g). By definition, it readily follows that $P_{n}\left(\rho_{1}\right)\subseteq P_{n}\left(\rho_{2}\right)$ if $1\leq \rho_{1}<\rho_{2}$. By the definition in (77), for a fixed integer n≥2, it follows that the function $u_{f}\left(n,\cdot \right)$ is monotonically increasing on [1,∞). The limit in the left side of (90) therefore exists. Since $D_{f}\left(Q∥U_{n}\right)$ is convex in *Q*, its maximum over the convex set of probability mass functions $Q\in P_{n}$ is obtained at one of the vertices of the simplex $P_{n}$. Hence, a maximum of $D_{f}\left(Q∥U_{n}\right)$ over this set is attained at $Q^{*}=\left(q_{1}^{*},\ldots ,q_{n}^{*}\right)$ with $q_{i}^{*}=1$ for some i∈{1,…,n}, and $q_{j}^{*}=0$ for j≠i. In the latter case,

(A215) $$\begin{array}{c} D_{f}\left(Q^{*}∥U_{n}\right)=\frac{1}{n}\sum_{k=1}^{n}f\left(nq_{k}^{*}\right)=\frac{1}{n}\;\left[(n-1)f(0)+f(n)\right]. \end{array}$$

Note that $Q^{*}\notin \underset{\rho \geq 1}{⋃}P_{n}\left(\rho \right)$ (since the union of $\left\{P_{n}\left(\rho \right)\right\}$, for all ρ≥1, includes all the probability mass functions in $P_{n}$ which are *supported* on $A_{n}=\left\{1,\ldots ,n\right\}$, so $Q^{*}\in P_{n}$ is not an element of this union); hence, it follows that

(A216) $$\begin{array}{c} \lim_{\rho \to \infty }u_{f}\left(n,\rho \right)\leq \left(1-\frac{1}{n}\right)f\left(0\right)+\frac{f(n)}{n}. \end{array}$$

On the other hand, for every ρ≥1,

(A217) $$\begin{array}{cc} u_{f}\left(n,\rho \right) & \geq g_{f}^{\left(\rho \right)}\left(\frac{1}{n}\right) \end{array}$$

(A218) $$\begin{array}{cc} \; & =\frac{1}{n}\;f\left(\frac{\rho n}{n+\rho -1}\right)+\left(1-\frac{1}{n}\right)\;f\left(\frac{n}{n+\rho -1}\right) \end{array}$$

where (A217) holds due to the left-side inequality of (85), and (A218) is due to (84). Combining (A217) and (A218), and the continuity of *f* at zero (by the continuous extension of the convex function *f* at zero), yields (by letting ρ→∞)

(A219) $$\begin{array}{c} \lim_{\rho \to \infty }u_{f}\left(n,\rho \right)\geq \left(1-\frac{1}{n}\right)f\left(0\right)+\frac{f(n)}{n}. \end{array}$$

Combining (A216) and (A219) gives (90) for every integer n≥2. In order to get an upper bound on the convergence rate in (90), suppose that f(0)<∞, *f* is differentiable on (0,n), and $K_{n}:=\underset{t\in (0,n)}{\sup}\;\left|f^{\prime }\left(t\right)\right|<\infty$. For every ρ≥1, we get

(A220) $$\begin{array}{cc} 0 & \leq \lim_{\rho^{\prime }\to \infty }\left\{u_{f}\left(n,\rho^{\prime }\right)\right\}-u_{f}\left(n,\rho \right) \end{array}$$

(A221) $$\begin{array}{cc} \; & \leq \frac{1}{n}\left[f\left(n\right)-f\left(\frac{\rho n}{n+\rho -1}\right)\right]+\left(1-\frac{1}{n}\right)\left[f\left(0\right)-f\left(\frac{n}{n+\rho -1}\right)\right] \end{array}$$

(A222) $$\begin{array}{cc} \; & \leq \frac{K_{n}}{n}\left(n-\frac{\rho n}{n+\rho -1}\right)+\left(1-\frac{1}{n}\right)\frac{K_{n}\;n}{n+\rho -1} \end{array}$$

(A223) $$\begin{array}{cc} \; & =\frac{2K_{n}\;\left(n-1\right)}{n+\rho -1}, \end{array}$$

where (A220) holds since the sets ${\left\{P_{n}\left(\rho \right)\right\}}_{\rho \geq 1}$ are monotonically increasing in ρ; (A221) follows from (A216)–(A218); (A222) holds by the assumption that $\left|f^{\prime }\left(t\right)\right|\leq K_{n}$ for all t∈(0,n), by the mean value theorem of Lagrange, and since $0<\frac{n}{n+\rho -1}\leq \frac{\rho n}{n+\rho -1}\leq n$ for all ρ≥1 and n∈N. This proves (91).

We next prove Item (h). Setting $P:=U_{n}$ yields P≺Q for every probability mass function *Q* which is supported on {1,…,n}. Since $q_{\min}+\left(n-1\right)q_{\max}\geq 1$ and $\left(n-1\right)q_{\min}+q_{\max}\leq 1$, and since by assumption $\frac{q_{\max}}{q_{\min}}\leq \rho$, it follows that

(A224) $$\begin{array}{cc} \left[nq_{\min},\;nq_{\max}\right] & \subseteq \left[\frac{n}{1+(n-1)\rho },\;\frac{\rho n}{n-1+\rho }\right]\subseteq \left[\frac{1}{\rho },\;\rho \right]. \end{array}$$

Combining the assumption in (92) with (A224) implies that

(A225) $$\begin{array}{c} m\leq f^{\prime \prime }\left(t\right)\leq M,\;\forall \;t\in \left[nq_{\min},\;nq_{\max}\right]. \end{array}$$

Hence, (26), (31) and (A225) yield

(A226) $$\begin{array}{c} \frac{1}{2}\;m\leq c_{f}\left(nq_{\min},nq_{\max}\right)\leq e_{f}\left(nq_{\min},nq_{\max}\right)\leq \frac{1}{2}\;M. \end{array}$$

The lower bound on $D_{f}\left(Q∥U_{n}\right)$ in the left side of (94) follows from a combination of (75), the left-side inequality in (A226), and ${∥P∥}_{2}^{2}=\frac{1}{n}$. Similarly, the upper bound on $D_{f}\left(Q∥U_{n}\right)$ in the right side of (95) follows from a combination of (74), the right-side inequality in (A226), and the equality ${∥P∥}_{2}^{2}=\frac{1}{n}$. The looser upper bound on $D_{f}\left(Q∥U_{n}\right)$ in the right side of (96), expressed as a function of *M* and ρ, follows by combining (74), (76), and the right-side inequality in (A226).

The tightness of the lower bound in the left side of (94) and the upper bound in the right side of (95) for the $\chi^{2}$ divergence is clear from the fact that M=m=2 if $f\left(t\right)={\left(t-1\right)}^{2}$ for all t>0; in this case, $\chi^{2}\left(Q∥U_{n}\right)=n{∥Q∥}_{2}^{2}-1$.

To prove Item (i), suppose that the second derivative of *f* is upper bounded on (0,∞) with $f^{\prime \prime }\left(t\right)\leq M_{f}\in \left(0,\infty \right)$ for all t>0, and there is a need to assert that $D_{f}\left(Q∥U_{n}\right)\leq d$ for an arbitrary d>0. Condition (97) follows from (96) by solving the inequality $\frac{M_{f}\;{\left(\rho -1\right)}^{2}}{8\rho }\leq d$, with the variable ρ≥1, for given d>0 and $M_{f}>0$ (note that $M_{f}$ does not depend on ρ).

## Appendix G. Proof of Theorem 8

The proof of Theorem 8 relies on Theorem 6. For α∈(0,1)∪(1,∞), let $u_{\alpha }:\left(0,\infty \right)\to R$ be the non-negative and convex function given by (see, e.g., ([8], (2.1)) or ([16], (17)))

(A227) $$\begin{array}{c} u_{\alpha }\left(t\right):=\frac{t^{\alpha }-\alpha \left(t-1\right)-1}{\alpha (\alpha -1)},\;t>0, \end{array}$$

and let $u_{1}:\left(0,\infty \right)\to R$ be the convex function given by

(A228) $$\begin{array}{c} u_{1}\left(t\right):=\lim_{\alpha \to 1}u_{\alpha }\left(t\right)=t\log_{e}t+1-t,\;t>0. \end{array}$$

Let *P* and *Q* be probability mass functions which are supported on a finite set; without loss of generality, let their support be given by $A_{n}:=\left\{1,\ldots ,n\right\}$. Then, for α∈(0,1)∪(1,∞),

(A229) $$\begin{array}{c} D_{u_{\alpha }}\left(Q∥U_{n}\right)-D_{u_{\alpha }}\left(P∥U_{n}\right)\\ =\frac{1}{n}\sum_{i=1}^{n}u_{\alpha }\left(nQ(i)\right)-\frac{1}{n}\sum_{i=1}^{n}u_{\alpha }\left(nP(i)\right)\\ =\frac{n^{\alpha -1}}{\alpha (\alpha -1)}\left[\;\sum_{i=1}^{n}Q^{\alpha }\left(i\right)-\sum_{i=1}^{n}P^{\alpha }\left(i\right)\right]\\ =\frac{n^{\alpha -1}\left[S_{\alpha }\left(P\right)-S_{\alpha }\left(Q\right)\right]}{\alpha }, \end{array}$$

where

(A230) $$S_{\alpha }\left(P\right):=\{\begin{array}{cc} \frac{1}{1-\alpha }\left(\sum_{i=1}^{n}P^{\alpha }\left(i\right)-1\right), & \alpha \in (0,1)\cup (1,\infty ),\\ -\sum_{i=1}^{n}P\left(i\right)\;\log_{e}P\left(i\right), & \alpha =1. \end{array}$$

designates the order-α Tsallis entropy of a probability mass *P* defined on the set $A_{n}$. Equality (A229) also holds for α=1 by continuous extension.

In view of (26) and (31), since $u_{\alpha }^{\prime \prime }\left(t\right)=t^{\alpha -2}$ for all t>0, it follows that

(A231) $$c_{u_{\alpha }}\left(nq_{\min},\;nq_{\max}\right)=\{\begin{array}{cc} \frac{1}{2}\;n^{\alpha -2}\;q_{\max}^{\alpha -2}, & \mathrm{if}\;\alpha \in (0,2],\\ \frac{1}{2}\;n^{\alpha -2}\;q_{\min}^{\alpha -2}, & \mathrm{if}\;\alpha \in (2,\infty ), \end{array}$$

and

(A232) $$e_{u_{\alpha }}(nq_{\min},\;nq_{\max})=\{\begin{array}{cc} \frac{1}{2}\;n^{\alpha -2}\;q_{\min}^{\alpha -2}, & \mathrm{if}\;\alpha \in (0,2],\\ \frac{1}{2}\;n^{\alpha -2}\;q_{\max}^{\alpha -2}, & \mathrm{if}\;\alpha \in (2,\infty ). \end{array}$$

The combination of (74) and (75) under the assumption that *P* and *Q* are supported on $A_{n}$ and P≺Q, together with (A229), (A231) and (A232) gives (100)–(102). Furthermore, the left and right-side inequalities in (100) hold with equality if $c_{u_{\alpha }}\left(\cdot ,\cdot \right)$ in (A231) and $e_{u_{\alpha }}\left(\cdot ,\cdot \right)$ in (A232) coincide, which implies that the upper and lower bounds in (74) and (75) are tight in that case. Comparing $c_{u_{\alpha }}\left(\cdot ,\cdot \right)$ in (A231) and $e_{u_{\alpha }}\left(\cdot ,\cdot \right)$ in (A232) shows that they coincide if α=2.

To prove Item (b) of Theorem 8, let $P_{\varepsilon }$ and $Q_{\varepsilon }$ be probability mass functions supported on A={0,1} where $P_{\varepsilon }\left(0\right)=\frac{1}{2}+\varepsilon$, $Q_{\varepsilon }\left(0\right)=\frac{1}{2}+\beta \varepsilon$, and β>1 and $0<\varepsilon <\frac{1}{2\beta }$. This yields $P_{\varepsilon }≺Q_{\varepsilon }$. The result in (103) is proved by showing that, for all α>0,

(A233) $$\begin{array}{cc} \; & \lim_{\varepsilon \to 0^{+}}\frac{S_{\alpha }\left(P_{\varepsilon }\right)-S_{\alpha }\left(Q_{\varepsilon }\right)}{L(\alpha ,P_{\varepsilon },Q_{\varepsilon })}=1, \end{array}$$

(A234) $$\begin{array}{cc} \; & \lim_{\varepsilon \to 0^{+}}\frac{S_{\alpha }\left(P_{\varepsilon }\right)-S_{\alpha }\left(Q_{\varepsilon }\right)}{U(\alpha ,P_{\varepsilon },Q_{\varepsilon })}=1, \end{array}$$

which shows that the infimum and supremum in (103) can be even restricted to the binary alphabet setting. For every α∈(0,1)∪(1,∞),

(A235) $$\begin{array}{cc} S_{\alpha }\left(P_{\varepsilon }\right)-S_{\alpha }\left(Q_{\varepsilon }\right) & =\frac{1}{1-\alpha }\left(\sum_{i}P_{\varepsilon }^{\alpha }\left(i\right)-\sum_{i}Q_{\varepsilon }^{\alpha }\left(i\right)\right)\\ & =\frac{1}{1-\alpha }\left[{\left(\frac{1}{2}+\varepsilon \right)}^{\alpha }+{\left(\frac{1}{2}-\varepsilon \right)}^{\alpha }-{\left(\frac{1}{2}+\beta \varepsilon \right)}^{\alpha }-{\left(\frac{1}{2}-\beta \varepsilon \right)}^{\alpha }\right]\\ & =\alpha 2^{2-\alpha }\left(\beta^{2}-1\right)\varepsilon^{2}+O\left(\varepsilon^{4}\right), \end{array}$$

where (A235) follows from a Taylor series expansion around ε=0, and the passage in the limit where α→1 shows that (A235) also holds at α=1 (due to the continuous extension of the order-α Tsallis entropy at α=1). This implies that (A235) holds for all α>0. We now calculate the lower and upper bounds on $S_{\alpha }\left(P_{\varepsilon }\right)-S_{\alpha }\left(Q_{\varepsilon }\right)$ in (101) and (102), respectively.

- For α∈(0,2],
  (A236) $$\begin{array}{cc} L(\alpha ,P_{\varepsilon },Q_{\varepsilon }) & =\frac{1}{2}\;\alpha q_{\max}^{\alpha -2}\;\left(∥Q_{\varepsilon }{∥}_{2}^{2}-{∥P_{\varepsilon }∥}_{2}^{2}\right)\\ & =\frac{1}{2}\;\alpha {\left(\frac{1}{2}+\beta \varepsilon \right)}^{\alpha -2}\left[{\left(\frac{1}{2}+\beta \varepsilon \right)}^{2}+{\left(\frac{1}{2}-\beta \varepsilon \right)}^{2}-{\left(\frac{1}{2}+\varepsilon \right)}^{2}-{\left(\frac{1}{2}-\varepsilon \right)}^{2}\right]\\ & =\alpha 2^{2-\alpha }\left(\beta^{2}-1\right){\left(1+2\beta \varepsilon \right)}^{\alpha -2}. \end{array}$$
- For α∈(2,∞),
  (A237) $$\begin{array}{cc} L(\alpha ,P_{\varepsilon },Q_{\varepsilon })\; & =\frac{1}{2}\;\alpha q_{\min}^{\alpha -2}\;\left(∥Q_{\varepsilon }{∥}_{2}^{2}-{∥P_{\varepsilon }∥}_{2}^{2}\right)\\ & =\alpha 2^{2-\alpha }\left(\beta^{2}-1\right){\left(1-2\beta \varepsilon \right)}^{\alpha -2}. \end{array}$$
- Similarly, for α∈(0,2],
  (A238) $$\begin{array}{cc} U(\alpha ,P_{\varepsilon },Q_{\varepsilon }) & =\frac{1}{2}\;\alpha q_{\min}^{\alpha -2}\;\left(∥Q_{\varepsilon }{∥}_{2}^{2}-{∥P_{\varepsilon }∥}_{2}^{2}\right)\\ & =\alpha 2^{2-\alpha }\left(\beta^{2}-1\right){\left(1-2\beta \varepsilon \right)}^{\alpha -2}, \end{array}$$
  and, for α∈(2,∞),
  (A239) $$\begin{array}{cc} U(\alpha ,P_{\varepsilon },Q_{\varepsilon }) & =\frac{1}{2}\;\alpha q_{\max}^{\alpha -2}\;\left(∥Q_{\varepsilon }{∥}_{2}^{2}-{∥P_{\varepsilon }∥}_{2}^{2}\right)\\ & =\alpha 2^{2-\alpha }\left(\beta^{2}-1\right){\left(1+2\beta \varepsilon \right)}^{\alpha -2}. \end{array}$$

The combination of (A235)–(A237) yields (A233); similarly, the combination of (A235), (A238) and (A239) yields (A234).

## Appendix H. Proof of Theorem 9 and Corollary 1

### Appendix H.1. Proof of Theorem 9

The proof of the convexity property of Δ(·,ρ) in (149), with ρ>1, over the real line R relies on ([69], Theorem 2.1) which states that if *W* is a non-negative random variable, then

(A240) $$\lambda_{\alpha }:=\{\begin{array}{cc} \frac{\left(E\left[W^{\alpha }\right]-E^{\alpha }\left[W\right]\right)\;\log e}{\alpha (\alpha -1)}, & \alpha \neq 0,1\\ \log\left(E[W]\right)-E\left[\log W\right], & \alpha =0\\ E\left[W\log W\right]-E\left[W\right]\;\log\left(E[W]\right), & \alpha =1 \end{array}$$

is log-convex in α∈R. This property has been used to derive *f*-divergence inequalities (see, e.g., ([62], Theorem 20), [65,69]).

Let Q≪P, and let $W:=\frac{dQ}{dP}$ be the Radon-Nikodym derivative (*W* is a non-negative random variable). Let the expectations in the right side of (A240) be taken with respect to *P*. In view of the above statement from ([69], Theorem 2.1), this gives the log-convexity of $D_{A}^{\left(\alpha \right)}\left(Q∥P\right)$ in α∈R. Since log-convexity yields convexity, it follows that $D_{A}^{\left(\alpha \right)}\left(Q∥P\right)$ is convex in α over the real line. Let $P:=U_{n}$, and let $Q\in P_{n}\left(\rho \right)$; since Q≪P, it follows that $D_{A}^{\left(\alpha \right)}\left(Q∥U_{n}\right)$ is convex in α∈R. The pointwise maximum of a set of convex functions is a convex function, which implies that $\max_{Q\in P_{n}\left(\rho \right)}D_{A}^{\left(\alpha \right)}\left(Q∥U_{n}\right)$ is convex in α∈R for every integer n≥2. Since the pointwise limit of a convergent sequence of convex functions is convex, it follows that $\lim_{n\to \infty }\;\max_{Q\in P_{n}\left(\rho \right)}D_{A}^{\left(\alpha \right)}\left(Q∥U_{n}\right)$ is convex in α. This, by definition, is equal to Δ(α,ρ) (see (146)), which proves the convexity of this function in α∈R. From (149), for all ρ>1,

(A241) $$\begin{array}{cc} \Delta (1+\alpha ,\rho ) & =\frac{1}{(\alpha +1)\alpha }\left[\frac{{\left(-\alpha \right)}^{\alpha }{\left(\rho^{1+\alpha }-1\right)}^{1+\alpha }{\left(\rho -\rho^{1+\alpha }\right)}^{-\alpha }}{\left(\rho -1\right){\left(1+\alpha \right)}^{1+\alpha }}-1\right]\\ & =\frac{1}{\left(-\alpha )(-\alpha -1\right)}\left[\frac{{\left(1+\alpha \right)}^{-\alpha -1}{\left(\rho^{\alpha }\left(\rho -\rho^{-\alpha }\right)\right)}^{1+\alpha }{\left(\rho^{1+\alpha }\left(\rho^{-\alpha }-1\right)\right)}^{-\alpha }}{\left(\rho -1\right){\left(-\alpha \right)}^{-\alpha }}-1\right]\\ & =\frac{1}{\left(-\alpha )(-\alpha -1\right)}\left[\frac{{\left(1+\alpha \right)}^{-\alpha -1}{\left(\rho -\rho^{-\alpha }\right)}^{1+\alpha }{\left(\rho^{-\alpha }-1\right)}^{-\alpha }}{\left(\rho -1\right){\left(-\alpha \right)}^{-\alpha }}-1\right]\\ & =\Delta (-\alpha ,\rho ), \end{array}$$

which proves the symmetry property of Δ(α,ρ) around $\alpha =\frac{1}{2}$ for all ρ>1. The convexity in α over the real line, and the symmetry around $\alpha =\frac{1}{2}$ implies that Δ(α,ρ) gets its global minimum at $\alpha =\frac{1}{2}$, which is equal to $\frac{4{\left(\sqrt[{4}]{\rho }-1\right)}^{2}}{\sqrt{\rho }+1}$ for all ρ>1.

Inequalities (162) and (163) follow from ([8], Proposition 2.7); this proposition implies that, for every integer n≥2 and for all probability mass functions *Q* defined on $A_{n}:=\left\{1,\ldots ,n\right\}$,

(A242) $$\begin{array}{cc} \; & \alpha \;D_{A}^{\left(\alpha \right)}\left(Q∥U_{n}\right)\leq \beta \;D_{A}^{\left(\beta \right)}\left(Q∥U_{n}\right),\;0<\alpha \leq \beta <\infty , \end{array}$$

(A243) $$\begin{array}{cc} \; & \left(1-\beta \right)\;D_{A}^{\left(1-\beta \right)}\left(Q∥U_{n}\right)\leq \left(1-\alpha \right)\;D_{A}^{\left(1-\alpha \right)}\left(Q∥U_{n}\right),\;-\infty <\alpha \leq \beta <1. \end{array}$$

Inequalities (162) and (163) follow, respectively, by maximizing both sides of (A242) or (A243) over $Q\in P_{n}\left(\rho \right)$, and letting *n* tend to infinity.

For every α∈R, the function Δ(α,ρ) is monotonically increasing in ρ∈(1,∞) since (by definition) the set of probability mass functions ${\left\{P_{n}\left(\rho \right)\right\}}_{\rho \geq 1}$ is monotonically increasing (i.e., $P_{n}\left(\rho_{1}\right)\subseteq P_{n}\left(\rho_{2}\right)$ if $1\leq \rho_{1}<\rho_{2}<\infty$), and therefore the maximum of $D_{A}^{\left(\alpha \right)}\left(Q∥U_{n}\right)$ over $Q\in P_{n}\left(\rho \right)$ is a monotonically increasing function of ρ∈[1,∞); the limit of this maximum, as we let n→∞, is equal to Δ(α,ρ) in (149) for all ρ>1, which is therefore monotonically increasing in ρ over the interval (1,∞). The continuity of Δ(α,ρ) in both α and ρ is due to its expression in (149) with its continuous extension at α=0 and α=1 in (150). Since $P_{n}\left(1\right)=\left\{U_{n}\right\}$, it follows from the continuity of Δ(α,ρ) that

$$\lim_{\rho \to 1^{+}}\Delta \left(\alpha ,\rho \right)=D_{A}^{\left(\alpha \right)}\left(U_{n}∥U_{n}\right)=0.$$

### Appendix H.2. Proof of Corollary 1

For all α∈R and ρ>1,

$$\begin{array}{cc} \; & \lim_{n\to \infty }\max_{Q\in P_{n}\left(\rho \right)}D_{A}^{\left(\alpha \right)}\left(U_{n}∥Q\right) \end{array}$$

(A244) $$\begin{array}{cc} \; & =\lim_{n\to \infty }\max_{Q\in P_{n}\left(\rho \right)}D_{A}^{\left(1-\alpha \right)}\left(Q∥U_{n}\right) \end{array}$$

(A245) $$\begin{array}{cc} \; & =\Delta (1-\alpha ,\rho ) \end{array}$$

(A246) $$\begin{array}{cc} \; & =\Delta (\alpha ,\rho ), \end{array}$$

where (A244) holds due to the symmetry property in ([8], p. 36), which states that

(A247) $$\begin{array}{c} D_{A}^{\left(\alpha \right)}\left(P∥Q\right)=D_{A}^{\left(1-\alpha \right)}\left(Q∥P\right), \end{array}$$

for every α∈R and probability mass functions *P* and *Q*; (A245) is due to (146); finally, (A246) holds due to the symmetry property of Δ(·,ρ) around $\frac{1}{2}$ in Theorem 9 (a).

## Appendix I. Proof of (171)

In view of (154) and (155), it follows that the condition in (170) is satisfied if and only if $\rho \leq \rho^{*}$ where $\rho^{*}\in \left(1,\infty \right)$ is the solution of the equation

(A248) $$\begin{array}{c} \frac{\rho^{*}\log\rho^{*}}{\rho^{*}-1}-\log\left(\frac{e\rho^{*}\log_{e}\rho^{*}}{\rho^{*}-1}\right)=d\log e. \end{array}$$

with a fixed d>0. The substitution

(A249) $$\begin{array}{c} x:=\frac{\rho^{*}\log_{e}\rho^{*}}{\rho^{*}-1} \end{array}$$

leads to the equation

(A250) $$\begin{array}{c} x-\log_{e}x=d+1. \end{array}$$

Negation and exponentiation of both sides of (A250) gives

(A251) $$\begin{array}{c} \left(-x\right)e^{-x}=-e^{-d-1}. \end{array}$$

Since $\rho^{*}>1$ implies by (A249) that x>1, the proper solution for *x* is given by

(A252) $$\begin{array}{c} x=-W_{-1}\left(-e^{-d-1}\right),\;d>0, \end{array}$$

where $W_{-1}$ denotes the secondary real branch of the Lambert *W* function [37]; otherwise, the replacement of $W_{-1}$ in the right side of (A252) with the principal real branch $W_{0}$ yields x∈(0,1).

We next proceed to solve $\rho^{*}$ as a function of *x*. From (A249), letting $u:=\frac{1}{\rho^{*}}$ gives the equation $u=e^{(u-1)x}$, which is equivalent to

(A253) $$\begin{array}{cc} \left(-ux\right)e^{-ux} & =-xe^{-x} \end{array}$$

(A254) $$\begin{array}{cc} \; & =-e^{-d-1}, \end{array}$$

where (A254) follows from (A252) and by the definition of the Lambert *W* function (i.e., t=W(u) if and only if $te^{t}=u$). The solutions of (A253) are given by

(A255) $$\begin{array}{c} -ux=W_{-1}\left(-e^{-d-1}\right), \end{array}$$

and

(A256) $$\begin{array}{c} -ux=W_{0}\left(-e^{-d-1}\right), \end{array}$$

which (from (A252)) correspond, respectively, to u=1 and

(A257) $$\begin{array}{c} u=\frac{W_{0}\left(-e^{-d-1}\right)}{W_{-1}\left(-e^{-d-1}\right)}\in \left(0,1\right). \end{array}$$

Since $\rho^{*}\in \left(1,\infty \right)$ is equal to $\frac{1}{u}$, the reciprocal of the right side of (A257) gives the proper solution for $\rho^{*}$ (denoted by $\rho_{\max}^{\left(1\right)}\left(d\right)$ in (171)).

## Appendix J. Proof of (176), (177) and (180)

We first derive the upper bound on Φ(α,ρ) in (176) for $\alpha \geq e^{-\frac{3}{2}}$ and ρ≥1. For every $Q\in P_{n}\left(\rho \right)$, with an integer n≥2,

$$\begin{array}{cc} D_{f_{\alpha }}\left(Q∥U_{n}\right) & \leq \left[\log\left(\alpha +1\right)+\frac{3}{2}\log e-\frac{\log e}{\alpha +1}\right]\;\chi^{2}\left(Q∥U_{n}\right) \end{array}$$

(A258) $$\begin{array}{cc} & \;+\frac{\log e}{3(\alpha +1)}\left[\exp\left(2D_{3}\left(Q∥U_{n}\right)\right)-1\right]\\ \; & \leq \left[\log\left(\alpha +1\right)+\frac{3}{2}\log e-\frac{\log e}{\alpha +1}\right]\;\frac{{\left(\rho -1\right)}^{2}}{4\rho } \end{array}$$

(A259) $$\begin{array}{cc} \; & \;+\frac{\log e}{3(\alpha +1)}\left[\exp\left(2D_{3}\left(Q∥U_{n}\right)\right)-1\right] \end{array}$$

where (A258) follows from (65), and (A258) holds due to (159). By upper bounding the second term in the right side of (A259), for all $Q\in P_{n}\left(\rho \right)$,

(A260) $$\begin{array}{cc} D_{3}\left(Q∥U_{n}\right) & =\frac{1}{2}\log\left(1+6D_{A}^{\left(3\right)}\left(Q∥U_{n}\right)\right) \end{array}$$

(A261) $$\begin{array}{cc} \; & \leq \frac{1}{2}\log\left(1+6\Delta (3,\rho )\right) \end{array}$$

(A262) $$\begin{array}{cc} \; & =\frac{1}{2}\log\left(\frac{4{\left(\rho^{3}-1\right)}^{3}}{27\left(\rho -1\right){\left(\rho -\rho^{3}\right)}^{2}}\right) \end{array}$$

(A263) $$\begin{array}{cc} \; & =\frac{1}{2}\log\left(\frac{4{\left(\rho^{2}+\rho +1\right)}^{3}}{27\rho^{2}{\left(\rho +1\right)}^{2}}\right) \end{array}$$

where (A260) holds by setting α=3 in (156); (A261) follows from (135), (138) and (145); (A262) holds by setting α=3 in (149); finally, (A263) follows from the factorizations

$${\left(\rho^{3}-1\right)}^{3}={\left(\rho -1\right)}^{3}{\left(\rho^{2}+\rho +1\right)}^{3},\;\left(\rho -1\right){\left(\rho -\rho^{3}\right)}^{2}={\left(\rho -1\right)}^{3}\rho^{2}{\left(\rho +1\right)}^{2}.$$

Substituting the bound in the right side of (A263) into the second term of the bound on the right side of (A259) implies that, for all $Q\in P_{n}\left(\rho \right)$,

$$\begin{array}{cc} D_{f_{\alpha }}\left(Q∥U_{n}\right) & \leq \left[\log\left(\alpha +1\right)+\frac{3}{2}\log e-\frac{\log e}{\alpha +1}\right]\;\frac{{\left(\rho -1\right)}^{2}}{4\rho } \end{array}$$

(A264) $$\begin{array}{cc} \; & \;+\frac{\log e}{3(\alpha +1)}\left[\frac{4{\left(\rho^{2}+\rho +1\right)}^{3}}{27\rho^{2}{\left(\rho +1\right)}^{2}}-1\right]\\ \; & =\left[\log\left(\alpha +1\right)+\frac{3}{2}\log e-\frac{\log e}{\alpha +1}\right]\frac{{\left(\rho -1\right)}^{2}}{4\rho } \end{array}$$

(A265) $$\begin{array}{cc} \; & \;+\frac{\log e}{81(\alpha +1)}{\left(\frac{\left(\rho -1)(2\rho +1)(\rho +2\right)}{\rho (\rho +1)}\right)}^{2}, \end{array}$$

which therefore gives (176) by maximizing the left side of (A264) over $Q\in P_{n}\left(\rho \right)$, and letting *n* tend to infinity (see (174)).

We next derive the upper bound in (177). The second derivative of the convex function $f_{\alpha }:\left(0,\infty \right)\to R$ in (55) is upper bounded over the interval $\left[\frac{1}{\rho },\rho \right]$ by the positive constant M=2log(α+ρ)+3loge. From (96), it follows that for all $Q\in P_{n}\left(\rho \right)$ (with ρ≥1 and an integer n≥2) and $\alpha \geq e^{-\frac{3}{2}}$,

(A266) $$\begin{array}{c} D_{f_{\alpha }}\left(Q∥U_{n}\right)\leq \left[\log\left(\alpha +\rho \right)+\frac{3}{2}\log e\right]\;\frac{{\left(\rho -1\right)}^{2}}{4\rho }, \end{array}$$

which, from (174), yields (177).

We finally derive the upper bound in (180) by loosening the bound in (176). The upper bound in the right side of (176) can be rewritten as

(A267) $$\begin{array}{cc} \Phi (\alpha ,\rho ) & \leq \left[\frac{1}{4}\;\log\left(\alpha +1\right)+\frac{3}{8}\;\log e\right]\;\frac{{\left(\rho -1\right)}^{2}}{\rho }\\ & \;+\frac{\log e}{\alpha +1}\left[\frac{1}{81}{\left(2+\frac{2}{\rho }+\frac{1}{1+\rho }\right)}^{2}-\frac{1}{4\rho }\right]{\left(\rho -1\right)}^{2}. \end{array}$$

For all ρ≥1,

(A268) $$\begin{array}{c} \frac{1}{81}{\left(2+\frac{2}{\rho }+\frac{1}{1+\rho }\right)}^{2}-\frac{1}{4\rho }\leq \frac{4}{81}, \end{array}$$

which can be verified by showing that the left side of (A268) is monotonically increasing in ρ over the interval [1,∞), and it tends to $\frac{4}{81}$ as we let ρ→∞. Furthermore, for all ρ≥1,

(A269) $$\begin{array}{c} \frac{{\left(\rho -1\right)}^{2}}{\rho }\leq \min\left\{\rho -1,{\left(\rho -1\right)}^{2}\right\}. \end{array}$$

In view of inequalities (A268) and (A269), one gets (180) from (A267) (where the latter is an equivalent form of (176)).

## Appendix K. Proof of Theorem 10

We start by proving Item (a). In view of the variational representation of *f*-divergences (see ([70], Theorem 2.1), and ([71], Lemma 1)), if f:(0,∞)→R is convex with f(1)=0, and *P* and *Q* are probability measures defined on a set A, then

(A270) $$\begin{array}{c} D_{f}\left(P∥Q\right)=\underset{g:A\to R}{\sup}\left(E\left[g(X)\right]-E[\;\bar{f}\left(g(Y)\right)\right]), \end{array}$$

where X∼P and Y∼Q, and the supremum is taken over all measurable functions *g* under which the expectations are finite.

Let $P\in P_{n}\left(\rho \right)$, with ρ>1, and let $Q:=U_{n}$; these probability mass functions are defined on the set $A_{n}:=\left\{1,\ldots ,n\right\}$, and it follows that

(A271) $$\begin{array}{cc} u_{f}\left(n,\rho \right) & \geq D_{f}\left(P∥U_{n}\right) \end{array}$$

(A272) $$\begin{array}{cc} \; & \geq E\left[g(X)\right]-\frac{1}{n}\sum_{i=1}^{n}\bar{f}\left(g(i)\right), \end{array}$$

where (A271) holds by the definition in (77); (A272) holds due to (A270) with X∼P, and *Y* being an equiprobable random variable over $A_{n}$. This gives (187).

We next prove Item (b). As above, let f:(0,∞)→R be a convex function with f(1)=0. Let $\beta^{*}\in \Gamma_{n}\left(\rho \right)$ be a maximizer of the right side of (82). Then,

(A273) $$\begin{array}{cc} u_{f}\left(n,\rho \right) & =D_{f}\left(Q_{\beta^{*}}∥U_{n}\right) \end{array}$$

(A274) $$\begin{array}{cc} \; & =\frac{1}{n}\sum_{i=1}^{n}f\left(nQ_{\beta^{*}}\left(i\right)\right). \end{array}$$

Let ε>0 be selected arbitrarily. We have $\bar{\left(\bar{f}\right)}\equiv f$ (i.e., repeating twice the convex conjugate operation (see (186)) on a convex function *f*, returns *f* itself). From the convexity of *f*, it therefore follows that, for all t>0, there exists x∈R such that

(A275) $$\begin{array}{c} f\left(t\right)\leq tx-\bar{f}\left(x\right)+\varepsilon . \end{array}$$

Let

(A276) $$\begin{array}{c} t_{i}:=nQ_{\beta^{*}}\left(i\right),\;\forall \;i\in A_{n}, \end{array}$$

let $x:=x_{i}\left(\varepsilon \right)\in R$ be selected to satisfy (A275) with $t:=t_{i}$, and let the function $g_{\varepsilon }:A_{n}\to R$ be defined as

(A277) $$\begin{array}{c} g_{\varepsilon }\left(i\right)=x_{i}\left(\varepsilon \right),\;\forall \;i\in A_{n}. \end{array}$$

Consequently, it follows from (A275)–(A277) that for all such *i*

(A278) $$\begin{array}{c} f\left(nQ_{\beta^{*}}\left(i\right)\right)\leq nQ_{\beta^{*}}\left(i\right)\;g_{\varepsilon }\left(i\right)-\bar{f}\left(g_{\varepsilon }\left(i\right)\right)+\varepsilon . \end{array}$$

Let $P:=Q_{\beta^{*}}\in P_{n}\left(\rho \right)$ (see (80)), and X∼P. Then,

(A279) $$\begin{array}{cc} u_{f}\left(n,\rho \right) & =\frac{1}{n}\sum_{i=1}^{n}f\left(nQ_{\beta^{*}}\left(i\right)\right) \end{array}$$

(A280) $$\begin{array}{cc} \; & \leq \sum_{i=1}^{n}Q_{\beta^{*}}\left(i\right)\;g_{\varepsilon }\left(i\right)-\frac{1}{n}\sum_{i=1}^{n}\bar{f}\left(g_{\varepsilon }\left(i\right)\right)+\varepsilon \end{array}$$

(A281) $$\begin{array}{cc} \; & =E\left[g_{\varepsilon }\left(X\right)\right]-\frac{1}{n}\sum_{i=1}^{n}\bar{f}\left(g_{\varepsilon }\left(i\right)\right)+\varepsilon \end{array}$$

where (A279) holds due to (A273) and (A274); (A280) follows from (A278); (A281) holds since by assumption $P_{X}=Q_{\beta^{*}}$. This gives (188).

## Appendix L. Proof of Theorem 11

For y∈Y, let the *L*-size list of the decoder be given by $L\left(y\right)=\{x_{1}\left(y\right),\ldots ,x_{L}\left(y\right)\}$ with L<M. Then, the (average) list decoding error probability is given by

(A282) $$\begin{array}{c} P_{L}=E\left[P_{L}\left(Y\right)\right] \end{array}$$

where the conditional list decoding error probability, given that Y=y∈Y, is equal to

(A283) $$\begin{array}{c} P_{L}\left(y\right)=1-\sum_{\ell =1}^{L}P_{X|Y}\left(x_{\ell }\left(y\right)\;|\;y\right). \end{array}$$

For every y∈Y,

$$\begin{array}{cc} \; & D_{f}\left(P_{X|Y}\left(\cdot |y\right)\;∥\;U_{M}\right) \end{array}$$

(A284) $$\begin{array}{cc} \; & \geq D_{f}\left(\left[\;\sum_{\ell =1}^{L}P_{X|Y}\left(x_{\ell }\left(y\right)\;|\;y\right),\;1-\sum_{\ell =1}^{L}P_{X|Y}(x_{\ell }\left(y\right)\;\left|\;y\right)\right]\;∥\;\left[\frac{L}{M},\;1-\frac{L}{M}\right]\right) \end{array}$$

(A285) $$\begin{array}{cc} \; & =D_{f}\left(\left[1-P_{L}\left(y\right),\;P_{L}\left(y\right)\right]\;∥\;\left[\frac{L}{M},\;1-\frac{L}{M}\right]\right), \end{array}$$

where (A284) holds by the data-processing inequality for *f*-divergences, and since for every y∈Y

(A286) $$\begin{array}{c} \sum_{\ell =1}^{L}U_{M}\left(x_{\ell }\left(y\right)\right)=\sum_{\ell =1}^{L}\frac{1}{M}=\frac{L}{M}; \end{array}$$

(A285) is due to (A283). Hence, it follows that

$$\begin{array}{cc} \; & E\left[D_{f}\left(P_{X|Y}\left(\cdot |Y\right)\;∥\;U_{M}\right)\right] \end{array}$$

(A287) $$\begin{array}{cc} \; & \geq E\left[D_{f}\left(\left[1-P_{L}\left(Y\right),\;P_{L}\left(Y\right)\right]\;∥\;\left[\frac{L}{M},\;1-\frac{L}{M}\right]\right)\right] \end{array}$$

(A278) $$\begin{array}{cc} \; & =\frac{L}{M}\;E\left[f\left(\frac{M(1-P_{L}\left(Y\right))}{L}\right)\right]+\left(1-\frac{L}{M}\right)\;E\left[f\left(\frac{MP_{L}\left(Y\right)}{M-L}\right)\right] \end{array}$$

(A279) $$\begin{array}{cc} \; & \geq \frac{L}{M}\;f\left(\frac{M\;E[1-P_{L}\left(Y\right)]}{L}\right)+\left(1-\frac{L}{M}\right)\;f\left(\frac{M\;E[P_{L}\left(Y\right)]}{M-L}\right) \end{array}$$

(A290) $$\begin{array}{cc} \; & =\frac{L}{M}\;f\left(\frac{M\left(1-P_{L}\right)}{L}\right)+\left(1-\frac{L}{M}\right)\;f\left(\frac{MP_{L}}{M-L}\right), \end{array}$$

where (A287) holds by taking expectations in (A284) and (285) with respect to *Y*; (A288) holds by the definition of *f*-divergence, and the linearity of expectation operator; (A289) follows from the convexity of *f* and Jensen’s inequality; finally, (A290) holds by (A282).

## Appendix M. Proof of Corollary 3

Let α∈(0,1)∪(1,∞), and let y∈Y. The proof starts by applying Theorem 11 in the setting where Y=y is deterministic, and the convex function f:(0,∞)→R is given by $f:=u_{\alpha }$ in (139), i.e.,

(A291) $$\begin{array}{c} f\left(t\right)=\frac{t^{\alpha }-\alpha \left(t-1\right)-1}{\alpha (\alpha -1)},\;t\geq 0. \end{array}$$

In this setting, (192) is specialized to

(A292) $$\begin{array}{c} D_{f}\left(P_{X|Y}\left(\cdot |y\right)\;∥\;U_{M}\right)\geq \frac{L}{M}\;f\left(\frac{M\;(1-P_{L}\left(y\right))}{L}\right)+\left(1-\frac{L}{M}\right)\;f\left(\frac{MP_{L}\left(y\right)}{M-L}\right), \end{array}$$

where $P_{L}\left(y\right)$ is the conditional list decoding error probability given that Y=y. Substituting (A291) into the right side of (A292) gives

$$\begin{array}{cc} \; & \frac{L}{M}\;f\left(\frac{M\;(1-P_{L}\left(y\right))}{L}\right)+\left(1-\frac{L}{M}\right)\;f\left(\frac{MP_{L}\left(y\right)}{M-L}\right) \end{array}$$

(A293) $$\begin{array}{cc} \; & =\frac{1}{\alpha (\alpha -1)}\left[P_{L}^{\alpha }\left(y\right)\;{\left(1-\frac{L}{M}\right)}^{1-\alpha }+{\left(1-P_{L}\left(y\right)\right)}^{\alpha }\;{\left(\frac{L}{M}\right)}^{1-\alpha }-1\right] \end{array}$$

(A294) $$\begin{array}{cc} \; & =\frac{1}{\alpha (\alpha -1)}\left[\exp\left(\left(\alpha -1\right)\;d_{\alpha }\left(P_{L}\left(y\right)\;∥\;1-\frac{L}{M}\right)\right)-1\right], \end{array}$$

where (A294) follows from (203). Substituting (A291) into the left side of (A292) gives

$$\begin{array}{cc} \; & D_{f}\left(P_{X|Y}\left(\cdot |y\right)\;∥\;U_{M}\right) \end{array}$$

(A295) $$\begin{array}{cc} \; & =\frac{1}{M\alpha (\alpha -1)}\sum_{x\in X}\left[{\left(MP_{X|Y}\left(x|y\right)\right)}^{\alpha }-\alpha \left(MP_{X|Y}\left(x|y\right)-1\right)-1\right] \end{array}$$

(A296) $$\begin{array}{cc} \; & =\frac{1}{M\alpha (\alpha -1)}\left[M^{\alpha }\sum_{x\in X}P_{X|Y}^{\alpha }\left(x|y\right)-\alpha \underset{=0\;\;(|X|=M)}{\underset{︸}{\sum_{x\in X}\left(MP_{X|Y}\left(x|y\right)-1\right)}}-M\right] \end{array}$$

(A297) $$\begin{array}{cc} \; & =\frac{1}{\alpha (\alpha -1)}\left[M^{\alpha -1}\sum_{x\in X}P_{X|Y}^{\alpha }\left(x|y\right)-1\right] \end{array}$$

(A298) $$\begin{array}{cc} \; & =\frac{1}{\alpha (\alpha -1)}\left[\exp\left(\left(\alpha -1\right)\;\left[\log M-H_{\alpha }\left(X|Y=y\right)\right]\right)-1\right]. \end{array}$$

Substituting (A294) and (A298) into the right and left sides of (A292), and rearranging terms while relying on the monotonicity property of an exponential function gives

(A299) $$\begin{array}{c} H_{\alpha }\left(X|Y=y\right)\leq \log M-d_{\alpha }\left(P_{L}\left(y\right)\;∥\;1-\frac{L}{M}\right). \end{array}$$

We next obtain an upper bound on the Arimoto-Rényi conditional entropy.

$$\begin{array}{cc} \; & H_{\alpha }\left(X|Y\right) \end{array}$$

(A300) $$\begin{array}{cc} \; & =\frac{\alpha }{1-\alpha }\;\log\;\int_{Y}dP_{Y}\left(y\right)\;\exp\left(\frac{1-\alpha }{\alpha }\;H_{\alpha }\left(X|Y=y\right)\right) \end{array}$$

(A301) $$\begin{array}{cc} \; & \leq \frac{\alpha }{1-\alpha }\;\log\;\int_{Y}dP_{Y}\left(y\right)\;\exp\left(\frac{1-\alpha }{\alpha }\;\left[\log M-d_{\alpha }\left(P_{L}\left(y\right)\;∥\;1-\frac{L}{M}\right)\right]\right) \end{array}$$

(A302) $$\begin{array}{cc} \; & =\log M+\frac{\alpha }{1-\alpha }\;\log\int_{Y}dP_{Y}\left(y\right){\left[P_{L}^{\alpha }\left(y\right){\left(1-\frac{L}{M}\right)}^{1-\alpha }+{\left(1-P_{L}\left(y\right)\right)}^{\alpha }{\left(\frac{L}{M}\right)}^{1-\alpha }\right]}^{\frac{1}{\alpha }} \end{array}$$

where (A300) holds due to (202); (A301) follows from (A299), and (A302) follows from (203). By ([42], Lemma 1), it follows that the integrand in the right side of (A302) is convex in $P_{L}\left(y\right)$ if α>1; furthermore, it is concave in $P_{L}\left(y\right)$ if α∈(0,1). Invoking Jensen’s inequality therefore yields (see (A282))

(A303) $$\begin{array}{cc} H_{\alpha }\left(X|Y\right) & \leq \log M+\frac{\alpha }{1-\alpha }\;\log\left({\left[P_{L}^{\alpha }{\left(1-\frac{L}{M}\right)}^{1-\alpha }+{\left(1-P_{L}\right)}^{\alpha }{\left(\frac{L}{M}\right)}^{1-\alpha }\right]}^{\frac{1}{\alpha }}\right) \end{array}$$

(A304) $$\begin{array}{cc} \; & =\log M-\frac{1}{\alpha -1}\;\log\left(P_{L}^{\alpha }{\left(1-\frac{L}{M}\right)}^{1-\alpha }+{\left(1-P_{L}\right)}^{\alpha }{\left(\frac{L}{M}\right)}^{1-\alpha }\right) \end{array}$$

(A305) $$\begin{array}{cc} \; & =\log M-d_{\alpha }\left(P_{L}\;∥\;1-\frac{L}{M}\right), \end{array}$$

where (A303) follows from Jensen’s inequality, and (A305) follows from (203). This proves (205) and (206) for all α∈(0,1)∪(1,∞). The necessary and sufficient condition for (205) to hold with equality, as given in (207), follows from the proof of (A292) (see (A284)–(A286)), and from the use of Jensen’s inequality in (A303).

## Appendix N. Proof of Theorem 12

The proof of Theorem 12 relies on Theorem 1, and the proof of Theorem 11.

Let Z={0,1} and, without any loss of generality, let X={1,…,M}. For every y∈Y, define a deterministic transformation from X to Z such that every x∈L(y) is mapped to z=0, and every x∉L(y) is mapped to z=1. This corresponds to a conditional probability mass function, for every y∈Y, where $W_{Z|X}^{\left(y\right)}\left(z|x\right)=1$ if x∈L(y) and z=0, or if x∉L(y) and z=1; otherwise, $W_{Z|X}^{\left(y\right)}\left(z|x\right)=0$. Let $L\left(y\right):=\{x_{1}\left(y\right),\ldots ,x_{L}\left(y\right)\}$ with L<M. Then, for every y∈Y, a conditional probability mass function $P_{X|Y}\left(\cdot |y\right)$ implies that

(A306) $$\begin{array}{c} P_{Z}^{\left(y\right)}\left(z\right):=\sum_{x\in X}P_{X|Y}\left(x|y\right)\;W_{Z|X}^{\left(y\right)}\left(z|x\right),\;\forall \;z\in \left\{0,1\right\}, \end{array}$$

satisfies (see (A283))

(A307) $$\begin{array}{cc} \; & P_{Z}^{\left(y\right)}\left(0\right)=\sum_{\ell =1}^{L}P_{X|Y}\left(x_{\ell }\left(y\right)|y\right)=1-P_{L}\left(y\right), \end{array}$$

(A308) $$\begin{array}{cc} \; & P_{Z}^{\left(y\right)}\left(1\right)=P_{L}\left(y\right). \end{array}$$

Under the deterministic transformation $W_{Z|X}^{\left(y\right)}$ as above, the equiprobable distribution $Q_{X}^{\left(y\right)}=U_{M}$ (independently of y∈Y) is mapped to a Bernoulli distribution over the two-elements set Z where

(A309) $$\begin{array}{c} Q_{Z}^{\left(y\right)}=\left[\frac{L}{M},\;1-\frac{L}{M}\right],\;\forall \;y\in Y. \end{array}$$

Given Y=y∈Y, applying Theorem 1 with the transformation $W_{Z|X}^{\left(y\right)}$ as above gives that

(A310) $$\begin{array}{c} D_{f}\left(P_{X|Y}\left(\cdot |y\right)\;∥\;U_{M}\right)\\ \geq D_{f}\left(P_{Z}^{\left(y\right)}∥Q_{Z}^{\left(y\right)}\right)+c_{f}\left(\xi_{1}\left(y\right),\xi_{2}\left(y\right)\right)\left[\chi^{2}\left(P_{X|Y}\left(\cdot |y\right)\;∥\;U_{M}\right)-\chi^{2}\left(P_{Z}^{\left(y\right)}∥Q_{Z}^{\left(y\right)}\right)\right] \end{array}$$

where, from (18) and (19),

(A311) $$\begin{array}{cc} \; & \xi_{1}\left(y\right)=\min_{x\in X}\frac{P_{X|Y}\left(x|y\right)}{U_{M}\left(x\right)}=M\min_{x\in X}P_{X|Y}\left(x|y\right), \end{array}$$

(A312) $$\begin{array}{cc} \; & \xi_{2}\left(y\right)=\max_{x\in X}\frac{P_{X|Y}\left(x|y\right)}{U_{M}\left(x\right)}=M\max_{x\in X}P_{X|Y}\left(x|y\right). \end{array}$$

Since, from (212), (213), (A311) and (A312),

(A313) $$\begin{array}{c} \underset{y\in Y}{\inf}\xi_{1}\left(y\right)=M\underset{(x,y)\in X\times Y}{\inf}P_{X|Y}\left(x|y\right)=\xi_{1}^{*}, \end{array}$$

(A314) $$\begin{array}{c} \underset{y\in Y}{\sup}\xi_{2}\left(y\right)=M\underset{(x,y)\in X\times Y}{\sup}P_{X|Y}\left(x|y\right)=\xi_{2}^{*}, \end{array}$$

it follows from the definition of $c_{f}\left(\cdot ,\cdot \right)$ in (26) that for every y∈Y

(A315) $$\begin{array}{cc} c_{f}\left(\xi_{1}\left(y\right),\xi_{2}\left(y\right)\right) & \geq c_{f}\left(\xi_{1}^{*},\xi_{2}^{*}\right) \end{array}$$

(A316) $$\begin{array}{cc} \; & =\frac{1}{2}\underset{t\in I(\xi_{1}^{*},\xi_{2}^{*})}{\inf}f^{\prime \prime }\left(t\right) \end{array}$$

(A317) $$\begin{array}{cc} \; & \geq \frac{1}{2}\;m_{f} \end{array}$$

where the last inequality holds by the assumption in (211). Combining (A310) and (A315)–(A317) yields

(A318) $$\begin{array}{c} \;D_{f}\left(P_{X|Y}\left(\cdot |y\right)\;∥\;U_{M}\right)\geq D_{f}\left(P_{Z}^{\left(y\right)}∥Q_{Z}^{\left(y\right)}\right)+\frac{1}{2}m_{f}\left[\chi^{2}\left(P_{X|Y}\left(\cdot |y\right)\;∥\;U_{M}\right)-\chi^{2}\left(P_{Z}^{\left(y\right)}∥Q_{Z}^{\left(y\right)}\right)\right], \end{array}$$

for every y∈Y. Hence,

(A319) $$\;E\left[D_{f}\left(P_{X|Y}\left(\cdot |Y\right)\;∥\;U_{M}\right)\right]\geq E\left[D_{f}\left(P_{Z}^{\left(Y\right)}∥Q_{Z}^{\left(Y\right)}\right)\right]+\frac{1}{2}m_{f}\;E\left[\chi^{2}\left(P_{X|Y}\left(\cdot |Y\right)\;∥\;U_{M}\right)-\chi^{2}\left(P_{Z}^{\left(Y\right)}∥Q_{Z}^{\left(Y\right)}\right)\right]$$

where (A319) holds by taking expectations with respect to *Y* on both sides of (A318).

Referring to the first term in the right side of (A319) gives

(A320) $$\begin{array}{cc} E\left[D_{f}\left(P_{Z}^{\left(Y\right)}∥Q_{Z}^{\left(Y\right)}\right)\right]\; & =E\left[D_{f}\left(\left[1-P_{L}\left(Y\right),\;P_{L}\left(Y\right)\right]\;∥\;\left[\frac{L}{M},\;1-\frac{L}{M}\right]\right)\right] \end{array}$$

(A321) $$\begin{array}{cc} \; & \geq \frac{L}{M}\;f\left(\frac{M\left(1-P_{L}\right)}{L}\right)+\left(1-\frac{L}{M}\right)\;f\left(\frac{MP_{L}}{M-L}\right), \end{array}$$

where (A320) follows from (A307)–(A309), and (A321) holds due to (A288)–(A290).

Referring to the second term in the right side of (A319) gives

$$\begin{array}{cc} \; & E\left[\chi^{2}\left(P_{X|Y}\left(\cdot |Y\right)\;∥\;U_{M}\right)-\chi^{2}\left(P_{Z}^{\left(Y\right)}∥Q_{Z}^{\left(Y\right)}\right)\right] \end{array}$$

(A322) $$\begin{array}{cc} \; & =E\left[\chi^{2}\left(P_{X|Y}\left(\cdot |Y\right)\;∥\;U_{M}\right)-\chi^{2}\left(\left[1-P_{L}\left(Y\right),\;P_{L}\left(Y\right)\right]\;∥\;\left[\frac{L}{M},\;1-\frac{L}{M}\right]\right)\right] \end{array}$$

(A323) $$\begin{array}{cc} \; & =E\left[M\sum_{x\in X}P_{X|Y}^{2}\left(x|Y\right)-\frac{M{\left(1-P_{L}\left(Y\right)\right)}^{2}}{L}-\frac{MP_{L}^{2}\left(Y\right)}{M-L}\right] \end{array}$$

(A324) $$\begin{array}{cc} \; & =M\;E\left[\;\sum_{x\in X}P_{X|Y}^{2}\left(x|Y\right)\right]-\frac{M}{L}+\frac{2M}{L}\cdot E\left[P_{L}\left(Y\right)\right]-\left(\frac{M}{L}+\frac{M}{M-L}\right)E\left[P_{L}^{2}\left(Y\right)\right] \end{array}$$

(A325) $$\begin{array}{cc} \; & =M\;E\left[\;\sum_{x\in X}P_{X|Y}^{2}\left(x|Y\right)\right]-\frac{M\left(1-2P_{L}\right)}{L}-\frac{M^{2}\;E\left[P_{L}^{2}\left(Y\right)\right]}{L(M-L)}, \end{array}$$

where (A322) follows from (A306)–(A309); (A323) follows from (A16)–(A18); (A325) is due to (A282). Furthermore, we get (since $P_{L}\left(Y\right)\in \left[0,1\right]$)

(A326) $$\begin{array}{cc} \; & E\left[P_{L}^{2}\left(Y\right)\right]\leq E\left[P_{L}\left(Y\right)\right]=P_{L}, \end{array}$$

(A327) $$\begin{array}{cc} \; & E\left[P_{L}^{2}\left(Y\right)\right]\geq E^{2}\left[P_{L}\left(Y\right)\right]=P_{L}^{2}, \end{array}$$

and

(A328) $$\begin{array}{cc} E\left[\;\sum_{x\in X}P_{X|Y}^{2}\left(x|Y\right)\right]\; & =\int_{Y}dP_{Y}\left(y\right)\;\sum_{x\in X}P_{X|Y}^{2}\left(x|y\right) \end{array}$$

(A329) $$\begin{array}{cc} \; & =\int_{X\times Y}dP_{XY}\left(x,y\right)\;P\left(x|y\right) \end{array}$$

(A330) $$\begin{array}{cc} \; & =E\left[P_{X|Y}\left(X|Y\right)\right]. \end{array}$$

Combining (A322)–(A330) gives

$$\begin{array}{cc} \; & M{\left(E\left[P_{X|Y}\left(X|Y\right)\right]-\frac{1-P_{L}}{L}-\frac{P_{L}}{M-L}\right)}^{+} \end{array}$$

(A331) $$\begin{array}{cc} \; & \leq E\left[\chi^{2}\left(P_{X|Y}\left(\cdot |Y\right)\;∥\;U_{M}\right)-\chi^{2}\left(P_{Z}^{\left(Y\right)}∥Q_{Z}^{\left(Y\right)}\right)\right] \end{array}$$

(A332) $$\begin{array}{cc} \; & \leq M\left(E\left[P_{X|Y}\left(X|Y\right)\right]-\frac{{\left(1-P_{L}\right)}^{2}}{L}-\frac{P_{L}^{2}}{M-L}\right), \end{array}$$

which provides tight upper and lower bounds on $E\left[\chi^{2}\left(P_{X|Y}\left(\cdot |Y\right)\;∥\;U_{M}\right)-\chi^{2}\left(P_{Z}^{\left(Y\right)}∥Q_{Z}^{\left(Y\right)}\right)\right]$ if $P_{L}$ is small. Note that the lower bound on the left side of (A331) is non-negative since, by the data-processing inequality for the $\chi^{2}$ divergence, the right side of (A331) should be non-negative (see (A306)–(A309)). Finally, combining (A319)–(A332) yields (214), which proves Item (a).

For proving Item (b), the upper bound on the left side of (A326) is tightened. If the list decoder selects the *L* most probable elements from X given the value of Y∈Y, then $P_{L}\left(y\right)\leq 1-\frac{L}{M}$ for every y∈Y. Hence, the bound in (A326) is replaced by the tighter bound

(A333) $$\begin{array}{cc} \; & E\left[P_{L}^{2}\left(Y\right)\right]\leq \left(1-\frac{L}{M}\right)P_{L}. \end{array}$$

Combining (A322)–(A325), (A328)–(A330) and (A333) gives the following improved lower bound in the left side of (A331):

(A334) $$\begin{array}{c} M{\left(E\left[P_{X|Y}\left(X|Y\right)\right]-\frac{1-P_{L}}{L}\right)}^{+}\leq E\left[\chi^{2}\left(P_{X|Y}\left(\cdot |Y\right)\;∥\;U_{M}\right)-\chi^{2}\left(P_{Z}^{\left(Y\right)}∥Q_{Z}^{\left(Y\right)}\right)\right]. \end{array}$$

It is next shown that the operation ${\left(\cdot \right)}^{+}$ in the left side of (A334) is redundant. From (A282) and (A283),

(A335) $$\begin{array}{cc} P_{L} & =1-\sum_{\ell =1}^{L}E\left[P_{X|Y}\left(x_{\ell }\left(Y\right)\;|\;Y\right)\right] \end{array}$$

(A336) $$\begin{array}{cc} \; & =1-\sum_{\ell =1}^{L}\int_{Y}dP_{Y}\left(y\right)\;P_{X|Y}\left(x_{\ell }\left(y\right)\;|\;y\right) \end{array}$$

(A337) $$\begin{array}{cc} \; & =1-\int_{Y}dP_{Y}\left(y\right)\;\sum_{\ell =1}^{L}P_{X|Y}\left(x_{\ell }\left(y\right)\;|\;y\right), \end{array}$$

which then implies that

(A338) $$\begin{array}{cc} P_{L} & \geq 1-L\int_{Y}dP_{Y}\left(y\right)\;\sum_{\ell =1}^{L}P_{X|Y}^{2}\left(x_{\ell }\left(y\right)\;|\;y\right) \end{array}$$

(A339) $$\begin{array}{cc} \; & \geq 1-L\int_{Y}dP_{Y}\left(y\right)\;\sum_{x\in X}P_{X|Y}^{2}\left(x|y\right) \end{array}$$

(A340) $$\begin{array}{cc} \; & \geq 1-L\int_{X\times Y}dP_{XY}\left(x,y\right)\;P_{X|Y}\left(x|y\right) \end{array}$$

(A341) $$\begin{array}{cc} \; & =1-L\;E\left[P_{X|Y}\left(X|Y\right)\right], \end{array}$$

where (A338) is due the Cauchy-Schwarz inequality applied to the right side of (A337), and (A339) holds since L(y)⊆X for all y∈Y. From (A335)–(A341), $E\left[P_{X|Y}\left(X|Y\right)\right]\geq \frac{1-P_{L}}{L}$, which implies that the operation ${\left(\cdot \right)}^{+}$ in the left side of (A334) is indeed redundant. Similarly to the proof of (214) (see (A319)–(A321)), (A334) yields (215) while ignoring the operation ${\left(\cdot \right)}^{+}$ in the left side of (A334).

## Appendix O. Proof of Theorem 13

For every y∈Y, let the *M* elements of X be sorted in decreasing order according to the conditional probabilities $P_{X|Y}\left(\cdot |y\right)$. Let $x_{\ell }\left(y\right)$ be the *ℓ*-th most probable element in X given Y=y, i.e.,

(A342) $$\begin{array}{c} P_{X|Y}\left(x_{1}\left(y\right)\;|y\right)\geq P_{X|Y}\left(x_{2}\left(y\right)\;|y\right)\geq \ldots \geq P_{X|Y}\left(x_{M}\left(y\right)\;|y\right). \end{array}$$

The conditional list decoding error probability, given Y=y, satisfies

(A343) $$\begin{array}{cc} P_{L}\left(y\right) & \geq 1-\sum_{\ell =1}^{\left|L(y)\right|}P_{X|Y}\left(x_{\ell }\left(y\right)\;|y\right) \end{array}$$

(A344) $$\begin{array}{cc} \; & :=P_{L}^{\left(\mathrm{opt}\right)}\left(y\right), \end{array}$$

and the (average) list decoding error probability satisfies $P_{L}\geq P_{L}^{\left(\mathrm{opt}\right)}$. Let $U_{M}$ denote the equiprobable distribution on X, and let $g_{\gamma }:\left[0,\infty \right)\to R$ be given by $g_{\gamma }\left(t\right):={\left(t-\gamma \right)}^{+}$ with γ≥1, where $u^{+}:=\max\left\{u,0\right\}$ for u∈R. The function $g_{\gamma }\left(\cdot \right)$ is convex, and $g_{\gamma }\left(1\right)=0$ for γ≥1; the *f*-divergence $D_{g_{\gamma }}\left(\cdot ∥\cdot \right)$ is named as the $E_{\gamma }$ divergence (see, e.g., [54]), i.e.,

(A345) $$\begin{array}{c} E_{\gamma }\left(P∥Q\right):=D_{g_{\gamma }}\left(P∥Q\right),\;\forall \;\gamma \geq 1, \end{array}$$

for all probability measures *P* and *Q*. For every y∈Y,

(A346) $$\begin{array}{cc} E_{\gamma }\left(P_{X|Y}\left(\cdot |y\right)\;∥\;U_{M}\right)\; & \geq E_{\gamma }\left(\left[1-P_{L}^{\left(\mathrm{opt}\right)}\left(y\right),\;P_{L}^{\left(\mathrm{opt}\right)}\left(y\right)\right]\;∥\;\left[\frac{\left|L(y)\right|}{M},\;1-\frac{\left|L(y)\right|}{M}\right]\right) \end{array}$$

(A347) $$\begin{array}{cc} \; & =\frac{\left|L(y)\right|}{M}\cdot g_{\gamma }\left(\frac{M\left(1-P_{L}^{\left(\mathrm{opt}\right)}\left(y\right)\right)}{\left|L(y)\right|}\;\right)+\left(1-\frac{\left|L(y)\right|}{M}\right)\;g_{\gamma }\left(\frac{M\;P_{L}^{\left(\mathrm{opt}\right)}\left(y\right)}{M-|L(y)|}\right), \end{array}$$

where (A346) holds due to the data-processing inequality for *f*-divergences, and because of (A344); (A347) holds due to (A345). Furthermore, in view of (A342) and (A344), it follows that $\frac{M\;P_{L}^{\left(\mathrm{opt}\right)}\left(y\right)}{M-|L(y)|}\leq 1$ for all y∈Y; by the definition of $g_{\gamma }$, it follows that

(A348) $$\begin{array}{c} g_{\gamma }\left(\frac{M\;P_{L}^{\left(\mathrm{opt}\right)}\left(y\right)}{M-|L(y)|}\right)=0,\;\forall \;\gamma \geq 1. \end{array}$$

Substituting (A348) into the right side of (A347) gives that, for all y∈Y,

(A349) $$\begin{array}{cc} E_{\gamma }\left(P_{X|Y}\left(\cdot |y\right)\;∥\;U_{M}\right)\; & \geq \frac{\left|L(y)\right|}{M}\cdot g_{\gamma }\left(\frac{M\left(1-P_{L}^{\left(\mathrm{opt}\right)}\left(y\right)\right)}{\left|L(y)\right|}\;\right) \end{array}$$

(A350) $$\begin{array}{cc} \; & ={\left(1-P_{L}^{\left(\mathrm{opt}\right)}\left(y\right)-\frac{\gamma \;|L(y)|}{M}\right)}^{+}. \end{array}$$

Taking expectations with respect to *Y* in (A349) and (A350), and applying Jensen’s inequality to the convex function $f\left(u\right):={\left(u\right)}^{+}$, for u∈R, gives

(A351) $$\begin{array}{cc} E\left[E_{\gamma }\left(P_{X|Y}\left(\cdot |Y\right)\;∥\;U_{M}\right)\right]\; & \geq E\left[{\left(1-P_{L}^{\left(\mathrm{opt}\right)}\left(Y\right)-\frac{\gamma \;|L(Y)|}{M}\right)}^{+}\right] \end{array}$$

(A352) $$\begin{array}{cc} \; & \geq {\left(1-E\left[P_{L}^{\left(\mathrm{opt}\right)}\left(Y\right)\right]-\frac{\gamma \;E\left[|L(Y)|\right]}{M}\right)}^{+} \end{array}$$

(A353) $$\begin{array}{cc} \; & ={\left(1-P_{L}^{\left(\mathrm{opt}\right)}-\frac{\gamma \;E\left[|L(Y)|\right]}{M}\right)}^{+} \end{array}$$

(A354) $$\begin{array}{cc} \; & \geq 1-P_{L}^{\left(\mathrm{opt}\right)}-\frac{\gamma \;E\left[|L(Y)|\right]}{M}. \end{array}$$

On the other hand, the left side of (A351) is equal to

$$\begin{array}{cc} \; & E\left[E_{\gamma }\left(P_{X|Y}\left(\cdot |Y\right)\;∥\;U_{M}\right)\right] \end{array}$$

(A355) $$\begin{array}{cc} \; & =E\left[\frac{1}{M}\sum_{x\in X}{\left(MP_{X|Y}\left(x|Y\right)-\gamma \right)}^{+}\right] \end{array}$$

(A356) $$\begin{array}{cc} \; & =E\left[\;\sum_{x\in X}{\left(P_{X|Y}\left(x|Y\right)-\frac{\gamma }{M}\right)}^{+}\right] \end{array}$$

(A357) $$\begin{array}{cc} \; & =\frac{1}{2}\;E\left[\;\sum_{x\in X}\left\{\left|P_{X|Y}\left(x|Y\right)-\frac{\gamma }{M}\right|+P_{X|Y}\left(x|Y\right)-\frac{\gamma }{M}\right\}\right] \end{array}$$

(A358) $$\begin{array}{cc} \; & =\frac{1}{2}\;E\left[\;\sum_{x\in X}\left|P_{X|Y}\left(x|Y\right)-\frac{\gamma }{M}\right|\right]+\frac{1}{2}\left(1-\gamma \right), \end{array}$$

where (A355) is due to (A345), and since $U_{M}\left(x\right)=\frac{1}{M}$ for all x∈X; (A356) and (A357) hold, respectively, by the simple identities ${\left(cu\right)}^{+}=c\;u^{+}$, and $u^{+}=\frac{1}{2}\left(|u|+u\right)$ for c≥0 and u∈R; finally, (A358) holds since

$$\sum_{x\in X}\left(P_{X|Y}\left(x|y\right)-\frac{\gamma }{M}\right)=-\gamma +\sum_{x\in X}P_{X|Y}\left(x|y\right)=1-\gamma ,$$

for all y∈Y. Substituting (A355)–(A358) and rearranging terms gives that

(A359) $$\begin{array}{c} P_{L}\geq P_{L}^{\left(\mathrm{opt}\right)}\geq \frac{1+\gamma }{2}-\frac{\gamma \;E\left[|L(Y)|\right]}{M}-\frac{1}{2}\;E\left[\;\sum_{x\in X}\left|P_{X|Y}\left(x|Y\right)-\frac{\gamma }{M}\right|\right], \end{array}$$

which is the lower bound on the list decoding error probability in (222).

We next proceed to prove the sufficient conditions for equality in (222). First, if for all y∈Y, the list decoder selects the |L(y)| most probable elements in X given that Y=y, then equality holds in (A359). In this case, for all y∈Y, $L\left(y\right):=\{x_{1}\left(y\right),\ldots ,x_{\left|L(y)\right|}\}$ where $x_{\ell }\left(y\right)$ denotes the *ℓ*-th most probable element in X, given Y=y, with ties in probabilities which are resolved arbitrarily (see (A342)). Let γ≥1. If, for every y∈Y, $P_{X|Y}(x_{\ell }\left(y\right)\;\left|y\right)$ is fixed for all ℓ∈{1,…,|L(y)|} and $P_{X|Y}(x_{\ell }\left(y\right)\;\left|y\right)$ is fixed for all ℓ∈{|L(y)|+1,…,M}, then equality holds in (A346) (and therefore equalities also hold in (A349) and (A351)). For all y∈Y, let the common values of the conditional probabilities $P_{X|Y}\left(\cdot |y\right)$ over each of these two sets, respectively, be equal to α(y) and β(y). Then,

(A360) $$\begin{array}{c} \alpha \left(y\right)\;|L\left(y\right)|+\beta \left(y\right)\;(M-\left|L\left(y\right)|\right)=\sum_{x\in X}P_{X|Y}\left(x|y\right)=1, \end{array}$$

which gives the condition in (223). Furthermore, if for all y∈Y, $1-P_{L}^{\left(\mathrm{opt}\right)}\left(y\right)-\frac{\gamma \;|L(y)|}{M}\geq 0$, then the operation ${\left(\cdot \right)}^{+}$ in the right side of (A351) is redundant, which causes (A352) to hold with equality as an expectation of a linear function; furthermore, also (A354) holds with equality in this case (since an expectation of a non-negative and bounded function is non-negative and finite). By (223) and (A344), it follows that $P_{L}^{\left(\mathrm{opt}\right)}\left(y\right)=1-\alpha \left(y\right)\;\left|L\left(y\right)\right|$ for all y∈Y, and therefore the satisfiability of (224) implies that equalities hold in (A352) and (A354). Overall, under the above condition, it therefore follows that (222) holds with equality. To verify it explicitly, under conditions (223) and (224) which have been derived as above, the right side of (222) satisfies

(A361) $$\begin{array}{c} \frac{1+\gamma }{2}-\frac{\gamma E[|L(Y)|]}{M}-\frac{1}{2}\;E\left[\;\sum_{x\in X}\;\left|P_{X|Y}\left(x|Y\right)-\frac{\gamma }{M}\right|\right]\\ =\frac{1+\gamma }{2}-\frac{\gamma E[|L(Y)|]}{M}\\ \;-\frac{1}{2}\;E\left[\left(\alpha \left(Y\right)-\frac{\gamma }{M}\right)\;\left|L\left(Y\right)\right|+\left(\frac{\gamma }{M}-\frac{1-\alpha (Y)\;|L(Y)|}{M-|L(Y)|}\right)\left(M-|L(Y)|\right)\right] \end{array}$$

(A362) $$\begin{array}{cc} \; & =1-E\left[\alpha (Y)\;|L(Y)|\right] \end{array}$$

(A363) $$\begin{array}{cc} \; & =E\left[1-\sum_{\ell =1}^{\left|L(Y)\right|}P_{X|Y}\left(x_{\ell }\left(Y\right)\;|Y\right)\right] \end{array}$$

(A364) $$\begin{array}{cc} \; & =P_{L}, \end{array}$$

where (A361) holds since, under (224), it follows that $0\leq \frac{1-\alpha (Y)\;|L(Y)|}{M-|L(Y)|}\leq \frac{1}{M}\leq \frac{\gamma }{M}$ for all γ≥1; (A362) holds by straightforward algebra, where γ is canceled out; (A363) holds by the condition in (223); finally, (A364) holds by (A282), (A283) and (A342). This indeed explicitly verifies that the conditions in Theorem 13 yield an equality in (222).

## Appendix P. Proofs of Theorems Related to Tunstall Trees

### Appendix P.1. Proof of Theorem 14

Theorem 14 (a) follows from (226) (see ([38], Corollary 1)).

By ([72], Lemma 6), the ratio of the maximal to minimal positive masses of $P_{\ell }$ is upper bounded by the reciprocal of the minimal probability mass of the source symbols. Theorem 14 (b) is therefore obtained from Theorem 7 (c). Theorem 14 (c) consequently holds due to Theorem 7 (d); the bound in the right side of (233), which holds for every number of leaves *n* in the Tunstall tree, is equal to the limit of the upper bound in the right side of (232) when we let n→∞.

Theorem 14 (d) relies on ([16], Theorem 11) and the definition in (231), providing an integral representation of an *f*-divergence in (234) under the conditions in Item (d).

### Appendix P.2. Proof of Theorem 15

In view of ([33], Theorem 4), if the fixed length of the codewords of the Tunstall code is equal to *m*, then the compression rate *R* of the code satisfies

(A365) $$\begin{array}{c} R\leq \frac{\left\lceil \log_{\left|X\right|}n\right\rceil \;H\left(P\right)}{\log_{\left|X\right|}n-\left[\frac{\rho \log\rho }{\rho -1}-\log\left(\frac{e\rho \log_{e}\rho }{\rho -1}\right)\right]\frac{1}{\log|X|}}, \end{array}$$

where H(P) denotes the Shannon entropy of the memoryless and stationary discrete source, $\rho :=\frac{1}{p_{\min}}$, *n* is the number of leaves in Tunstall tree, and the logarithms with an unspecified base can be taken on an arbitrary base in the right side of (A365). By the setting in Theorem 15, the construction of the Tunstall tree satisfies $n\leq {\left|X\right|}^{m}<n+\left(D-1\right)$. Hence, if D=2, then $\log_{\left|X\right|}n=m$; if D>2, then $\lceil \log_{\left|X\right|}n\rceil =m$ (since the length of the codewords is *m*), and $\log_{\left|X\right|}n>m+\log_{\left|X\right|}\left(1-\frac{D-1}{{\left|X\right|}^{m}}\right)$. Combining this with (A365) yields

(A366) $$R\leq \{\begin{array}{cc} \frac{mH(P)}{m+\left\{\log\left(1-\frac{D-1}{{\left|X\right|}^{m}}\right)-\left[\frac{\rho \log\rho }{\rho -1}-\log\left(\frac{e\rho \log_{e}\rho }{\rho -1}\right)\right]\right\}\frac{1}{\log|X|}}, & \mathrm{if}\;D>2,\\ \frac{mH(P)}{m-\left[\frac{\rho \log\rho }{\rho -1}-\log\left(\frac{e\rho \log_{e}\rho }{\rho -1}\right)\right]\frac{1}{\log|X|}}, & \mathrm{if}\;D=2. \end{array}$$

In order to assert that R≤(1+ε)H(P), it is requested that the right side of (A366) does not exceed (1+ε)H(P). This gives

(A367) $$\begin{array}{c} \frac{\rho \log\rho }{\rho -1}-\log\left(\frac{e\rho \log_{e}\rho }{\rho -1}\right)\leq d\log e, \end{array}$$

where *d* is given in (235). In view of the part in Section 3.3.2 with respect to the exemplification of Theorem 7 for the relative entropy, and the related analysis in Appendix I, the condition in (A367) is equivalent to $\rho \leq \rho_{\max}^{\left(1\right)}\left(d\right)$ where $\rho_{\max}^{\left(1\right)}\left(d\right)$ is defined in (171). Since $p_{\min}=\frac{1}{\rho }$, it leads to the sufficient condition in (236) for the requested compression rate *R* of the Tunstall code.
